# Genetic rescue in a plant polyploid complex: Case study on the importance of genetic and trait data for conservation management

**DOI:** 10.1002/ece3.4039

**Published:** 2018-04-25

**Authors:** Alexander N. Schmidt‐Lebuhn, David J. Marshall, Brad Dreis, Andrew G. Young

**Affiliations:** ^1^ CSIRO, Centre for Australian National Biodiversity Research Canberra ACT Australia; ^2^ E2M Consulting West End QLD Australia

**Keywords:** polyploidy, *Rutidosis lanata*, self‐incompatibility

## Abstract

Knowledge of the biology of rare plant species is indispensable to aid their survival and to inform efficient conservation actions, but in many cases relevant data are lacking. In addition, while studies of conservation genetics have provided a wealth of information on the considerations arising from inbreeding, mate limitation, or local adaptation, the impact of intraspecific polyploidy remains understudied. In this study, we examined the breeding system of the rare Australian daisy *Rutidosis lanata* (Asteraceae) and screened ten of its populations for their ploidy level to develop recommendations for management actions, in particular, with regard to seed sourcing and genetic rescue. We found *R. lanata* to represent a polyploid complex, with tetraploid, pentaploid and hexaploid individuals coexisting in the same species. Crossing experiments confirmed *R. lanata* to be self‐incompatible. Mate availability varied from c. 49% to c. 76% across populations. Most populations showed mate availability of c. 50%–70%, suggesting that mate limitation resulting from a lack of local genetic diversity may cause or at least contribute to reduced seed set. Crossing between populations resulted in significantly higher reproductive success for all populations except one, suggesting the possibility of genetic rescue through population mixing. However, the crossing experiments also showed that pentaploids suffer from a severely reduced paternal reproductive fitness. Any additional hybrids between tetraploids and pentaploids, as would be created by mixing populations with different genome copy numbers during conservation work, would consequently exacerbate mate limitation and thus reduce population viability. We conclude that seed set and thus population viability can be maximized by mixing populations with the same number of genome copies, but that populations with different numbers should be kept spatially separated. The case of *Rutidosis lanata* provides an example and a potential template for examining the conservation genetics of other species that may constitute polyploid complexes.

## 1 INTRODUCTION

Habitat loss, fragmentation, competition from invasive species and other pressures have reduced the population sizes of many plant species to the point where management is required to assist with their survival (Rossi et al., 2016). Management options include ex situ conservation, the creation of new wild populations through translocation or revegetation, and augmenting existing populations. To ensure the establishment of long‐term viable wild or ex situ populations and to guide efficient work practices, knowledge of the biology of a species is indispensable (Morris & Doak, 2002). Relevant information is, however, unavailable for many rare species, especially in species‐rich but understudied parts of the world (Young & Clarke, 2000).

Genetic structure has long been recognized as a crucial factor in conservation management. Genetic diversity is the basis of adaptive potential and resilience to change, increasing the long‐term viability of populations (Reed & Frankham, 2003). An increasing number of studies has argued for “climate ready” planning to anticipate future changes, especially in long‐lived plants (Broadhurst, Jones, Smith, North, & Guja, 2016). More generally, inbreeding depression is a concern in small and fragmented populations (Hedrick & Kalinowski, 2000). It is compounded in self‐incompatible plants (see below), as low diversity in self‐incompatibility alleles causes mate limitation (Young & Pickup, 2010).

On the other hand, organisms may be adapted to local conditions, potentially limiting how far they can be translocated or the degree to which populations can be mixed to increase genetic diversity (Edmands, 2007). If populations have differentiated to a sufficient degree, crossing them may result in outbreeding depression (Frankham et al., 2011). A special case is cytological races such as different ploidy levels in a polyploid complex, where mixed offspring may be sterile (see below).

Taken together, these insights provide a framework to guide decisions on seed sourcing and whether or not to mix existing populations. The problem of polyploid complexes, however, remains an understudied aspect of conservation (Severns, Bradford, & Liston, 2013; Wallace et al., 2017), and in particular, its interaction with self‐incompatibility. This study examined the breeding system and genetics of a rare species suspected to constitute a polyploid complex to explore how management guidelines have to be shaped to accommodate polyploidy.

### 1.1 Polyploidy

Polyploidy is the phenomenon of genome duplication. Most animals and vascular plants are diploid, that is, they have two genome copies. In many plant groups, however, polyploids can arise spontaneously through genome duplication, leading to the existence of populations or species with higher numbers of genome copies (Leitch & Bennett, 1997).

From the perspective of conservation management, the greatest concern around polyploidy is hybridization between populations with different numbers of genome copies. The offspring of a diploid and a tetraploid plant, for example, is triploid. Individuals with uneven numbers of genome copies are often partially or fully sterile due to failure of meiosis (Ramsey, Vaughton, Ascough, & Johnson, 2011).

Consequently, the formation of additional hybrids between plants with different (even) numbers of genome copies should be avoided to ensure that populations are genetically healthy and reproductively successful (Severns & Liston, 2008). In species whose populations have different ploidy levels, they or their seeds should not be mixed in revegetation and seed orcharding.

### 1.2 Self‐incompatibility

Many flowering plants exhibit self‐incompatibility (SI), a mechanism by which flowers reject the pollen grains of potential donors that exhibit the same genetic marker (S‐allele) as that possessed by the prospective pollen recipient (Hiscock & Tabah, 2003). There are two main forms of SI. In gametophytic self‐incompatibility (GSI) only the S‐allele carried by the pollen genome is recognized, allowing plants to mate as long as they do not share all S‐alleles (Newbigin, Anderson, & Clarke, 1993). Sporophytic self‐incompatibility (SSI) is more restrictive, as all S‐alleles carried by the pollen donor are potentially recognized (Heizmann, 1992), but complex dominance effects between S‐alleles can occur (Hiscock & McInnis, 2003).

The purpose of SI is to avoid inbreeding and the resultant risk of genetic disorders arising in offspring (Heizmann, 1992). In large and well‐connected populations, large numbers of S‐alleles are present and guarantee that most plants will have genetically compatible mates available. In small and isolated populations, however, little diversity in S‐alleles can be maintained, until most plants are surrounded by incompatible mates with which they share at least one S‐allele. This situation, which is characterized by low seed set and consequently low population viability, is called mate limitation (Young & Pickup, 2010).

Mate limitation can be managed by adding individuals or seeds from other, genetically distinct populations (Pickup & Young, 2007). This form of genetic rescue injects new S‐alleles into a genetically impoverished population, making new mates available and restoring seed set.

### 1.3 Study organism

**Figure 1:**
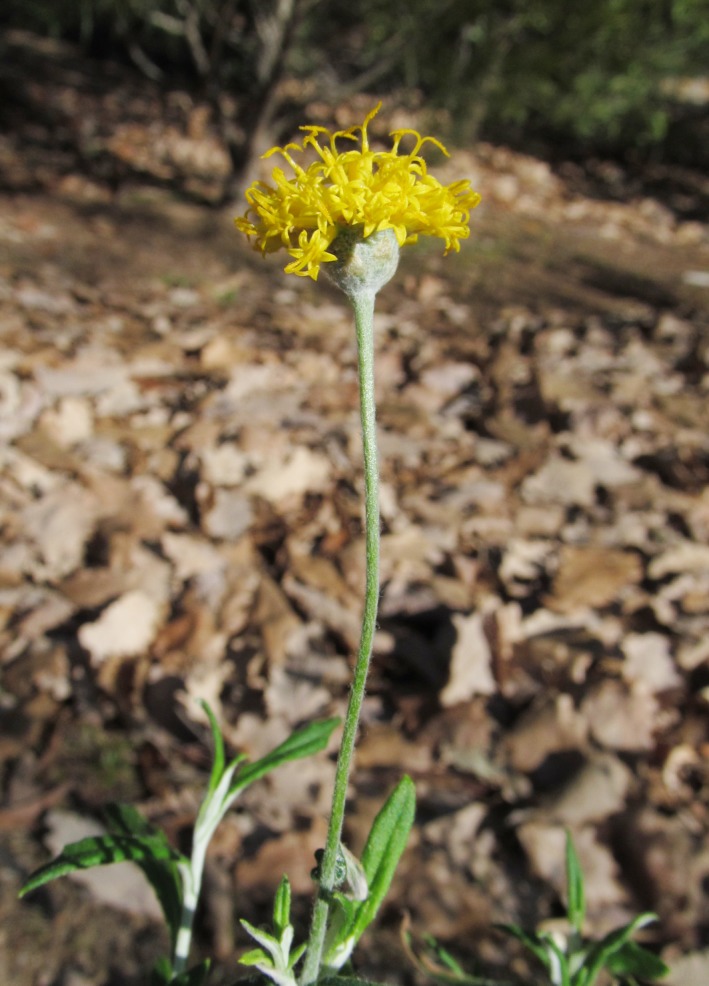
*Rutidosis lanata* (Asteraceae) is a rare herbaceous perennial endemic to southeastern Queensland, Australia

**Figure 2:**
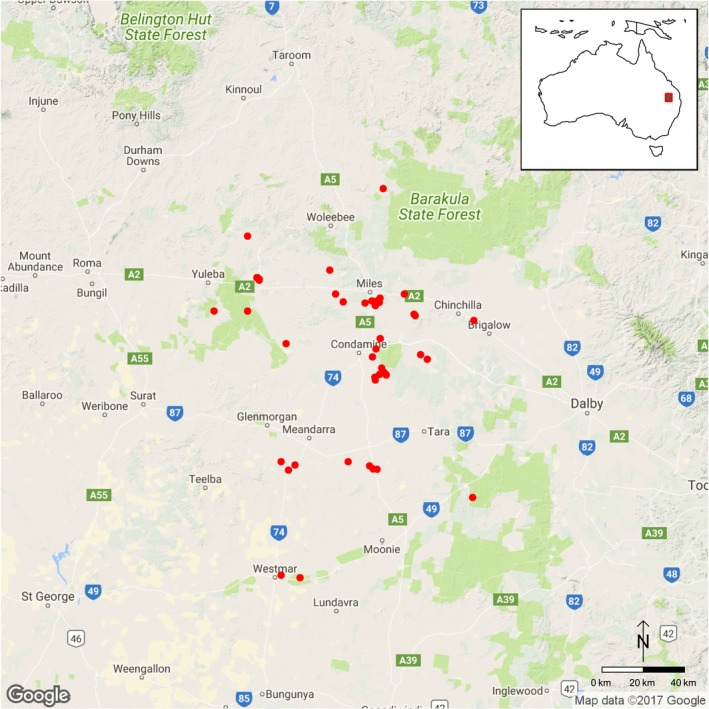
Approximate area of distribution of *Rutidosis lanata* indicated by records in the Atlas of Living Australia (ala.org.au). Open circles indicate towns, and red circles indicate specimen records

*Rutidosis lanata* A.E.Holland (Asteraceae: Gnaphalieae; Figure 1) is a perennial herbaceous daisy endemic to southeastern Queensland, Australia (Holland, 1994). Its range is restricted to an area of approximately 160 × 190 km from around Yuleba and Barakula State Forest in the north to around Westmar and Moonie in the south (Figure 2). Populations have been fragmented by land use changes, and recent resource development had the potential to impact on the species. *Rutidosis lanata* is listed as Near Threatened under the Queensland Nature Conservation Act 1992.

Very little, however, is known about its reproductive biology, ecology, and genetics. Individuals can expand clonally with underground rhizomes, at least over moderate distances (A.N.Schmidt‐Lebuhn, pers. obs.). Although pollination has not been studied, floral morphology (Figure 1) and opportunistic field observations of unidentified bees, flies, and beetles suggest generalist insect pollination. The fruits are large compared to those of related species, and the pappus, the parachuting organ of most Asteraceae, is vestigial, suggesting that dispersal distances may be limited.

Only one chromosome count of *R. lanata* has been published (Kokubugata & Holland, 2002). At 72 chromosomes, comparison with the chromosome numbers of diploid relatives suggests hexaploidy, that is, six genome copies. It remains unknown whether there is variation between populations, but close relatives of *R. lanata* are known to constitute polyploid complexes (Murray & Young, 2001).

Close relatives of *Rutidosis lanata* are known to be self‐incompatible (Young, Miller, Gregory, & Langston, 2000). Anecdotal observations suggest that the species may suffer from reduced seed set in the field and in the nursery (B. Dreis, pers. obs.). It is possible that mate limitation due to a scarcity of S‐alleles in the self‐incompatibility system is at least partly responsible, but the mating system of *R. lanata* remains unknown.

### 1.4 Aims of the study

The aim of this study was to generate basic information about the genetic makeup and reproductive biology of *Rutidosis lanata* that could be used to maximize long‐term viability of existing or new populations, as an example case for how polyploidy can affect conservation management, in particular, through its interaction with the breeding system. Specific aims were as follows. First, to determine whether *Rutidosis lanata* is a polyploid complex. If that was the case, to provide an understanding of the geographic distribution of ploidy levels. Second, to determine whether the species is self‐incompatible. If that was the case, to assess if there is evidence for mate limitation in selected populations and if that was the case, to test if mate availability can be increased by crossing between populations. Third, to develop concrete management recommendations for *Rutidosis lanata*, in particular, with regard to seed sourcing and whether populations should be mixed during translocation work.

## 2 METHODS

### 2.1 Study plants

One hundred and fifty three plants were collected by Brad Dreis, Liz Fisher, and Chays Ogston from ten populations southwest (Gilmore) and southeast (Chaplin 1 & 2, Little 1 & 2) of Miles and southeast of Condamine (Campbell 1‐5) (Table 1). This area is close to the center of the known range of the species.

**Table 1:** Study populations, ploidy levels, and crossing results

Population	Location	Tetraploids	Pentaploids	Hexaploids	S‐allele estimates	Mate availability in population	Mate availability when crossed with other populations	Improvement
Campbell 1	26.99188°S 150.237821°E	7	0	0	13–17	75.7%	71.9% (*n* = 32)	None
Campbell 2	27.019334°S 150.260247°E	10	0	0	15–19	53.9%	82.6% (*n* = 23)	28.7%
Campbell 3	27.022234°S 150.268114°E	12	0	0	17–21	56.3%	80.8% (*n* = 26)	24.5%
Campbell 4	27.007933°S 150.246333°E	9	4	1	11–14	48.6%	82.8% (*n* = 29)	34.2%
Campbell 5	27.008933°S 150.254492°E	2	7	1	N/A	N/A	N/A	N/A
Chaplin 1	26.708953°S 150.231738°E	0	0	9	30–32	76.4%	95.0% (*n* = 40)	18.6%
Chaplin 2	26.709605°S 150.239118°E	0	0	9	23–26	70.5%	81.2% (*n *= 33)	10.7%
Gilmore	26.72405°S 150.116542°E	7	1	4	9–14	56.1%	80.8% (*n* = 22)	34.7%
Little 1	26.715191°S 150.215878°E	4	0	5	17–21	70.0%	89.7% (*n* = 29)	19.7%
Little 2	26.701363°S 150.21888°E	0	0	11	24–29	64.4%	80.5% (*n* = 41)	16.1%

Crossing results, S‐allele numbers and mate availability refer only to the dominant ploidy level in each population, and to crosses at the same ploidy level for interpopulation crosses.

No analyses were conducted on Campbell 5 because most study plants from that population were pentaploids.

The plants were transferred to research greenhouses in Canberra, ACT, in autumn 2015, where they initially experienced some mortality, reducing the number of study plants to 106 at seven to twelve individuals per population. Plants were cultivated in 20 cm pots on commercial Australian native plant potting mix, watered as necessary, and on a diurnal temperature cycle.

### 2.2 Genome sizes

Genome sizes were measured in all study plants using flow cytometry (Doležel, Greilhuber, & Suda, 2007). Fresh leaf material was collected from living specimens and kept on ice. A single triploid clone of *Bellis perennis* L. (2C = 3.41 pg DNA) was used as a standard (Castelli, Miller, & Schmidt‐Lebuhn, 2017). Approximately 50 mg of *Rutidosis lanata* and 10 mg of standard leaf material were each placed in 1,200 μl of ice‐cold Modified Galbraith's buffer (Galbraith et al., 1983; Price, 2010) [4.58 g MgCl2, 2.1 g MOPSO, 4.44 g Citric Acid, 15.0 g PVP‐10, 0.5 ml Triton X‐100, 2.5 ml Tween 20, made up to 500 ml with distilled water. The pH was adjusted to 7.0–7.1 with 10 mol/L NaOH and filtered through a 0.22 μm filter, transferred to 15 ml tubes, and stored at −20°C] in a Petri dish and were manually chopped with a razor blade for approximately 30 s each to slice the leaf apart in parallel lines perpendicular to the main leaf vein. The chopped leaf material and buffer were gently mixed to release intact nuclei. The liquid was filtered through a 40‐μm cell strainer and then centrifuged at 2,000 rpm for 1 min. Supernatant was removed until approximately 400 μl were left, and cells were gently resuspended. The sample was mixed with 20 μl of 1 mg/ml propidium iodide, then loaded into a Beckman Coulter Cell Lab Quanta MPL flow cytometer equipped with a 488 nm laser at 22 mW and run at 22 μl/min. Preliminary experiments showed that addition of RNAse did not improve results. Histogram data were collected for both the internal standard and sample using the FL2 detector, and data analysis was performed with Beckman Coulter Cell Lab Quanta SC MPL analysis Software.

### 2.3 Chromosome counts

To verify that observed genome size differences were due to different ploidy levels, we counted chromosome numbers of selected specimens using an approach adapted from Murray and Young (2001). Several actively growing root tips were collected from selected study plants and were immediately placed in vials containing 0.5% colchicine solution. After refrigeration for approximately 4 hr, the root tips were transferred into freshly made fixative (3 parts 95% ethanol to 1 part glacial acetic acid [GAA]). They were refrigerated overnight, rinsed with water and transferred into 70% ethanol for long‐term storage at −20°C. To prepare squashes, two or three root tips were transferred to a new vial, rinsed to remove dirt, and softened for 10 min at 60°C in 1 mol/L hydrochloric acid. Subsequently, the root tips were rinsed with water and transferred into 45% GAA.

One or two root tips were placed on a glass slide, and the first c. 1 mm separated out while discarding the rest. Excess liquid was removed and replaced with a drop of FLP Orcein stain. Softened root tips were spread out by tapping with a brass rod for 30 s to 2 min. A coverslip was added and the sample heated over an open flame alcohol burner for 2–4 s. It was then squashed firmly under soft tissue paper. Approximate chromosome counts were made from digital photographs from a Zeiss Axio imager compound microscope at the 63× water immersion level.

### 2.4 Crossing experiments

To assess whether *Rutidosis lanata* was self‐incompatible or self‐compatible and to measure S‐allele numbers and mate limitation in populations, diallel crosses were conducted in every population. Flower heads were bagged with mesh bags at the bud stage to prevent uncontrolled pollination by insects entering the glasshouses. Cross‐pollination between any two individual heads chosen for an experiment was repeated three times over the course of five to 6 days to cover all individual, successively blooming flowers of both flower heads.

All plants were crossed with themselves by reciprocally pollinating two flower heads of the same plant, to test for self‐incompatibility. All plants of a population were crossed with each other, except when individual plants produced an insufficient number of flowers, to estimate mate availability and number of S‐alleles within populations. Finally, a representative number of interpopulation crosses was conducted to explore the improvement of mate availability through population mixing.

Approximately three to 5 weeks after pollination, when a seed head was shedding either ripe or abortive seeds, the entire seed head was collected and fertile seed (easily recognized by their large size and dark color) was counted.

R 3.3.2 was used for statistical evaluation as well as for creating maps using the ggmap library (Kahle & Wickham, 2013). As even self‐pollinated flowers may sometimes set very few fertile seeds (generally 0‐2), either because of pollen contamination or because of a failure of the self‐incompatibility mechanism, seed heads were scored as successfully pollinated only if the number of fertile seeds was larger than that produced by 95% of the heads subjected to selfing (including all replicates), that is, seven or more. Custom R scripts were used to remove replicate crosses of the same mother and father and to populate crossing diallels of each population. If replicates were inconsistent, a cross representing the majority was selected. If equal numbers of replicates succeeded and failed, the cross was scored as missing data.

Mate availability in a given group of plants was calculated by dividing the number of successfully pollinated seed heads by the number of all seed heads harvested from that group, excluding self‐pollinations. S‐allele numbers were inferred from the crossing diallels using a custom‐written Python script, assuming the existence of dominance effects in the female function and polyploidy of the S‐locus. Welch two‐sample t tests were used to test for differences in seed numbers between ploidy levels and sexes.

## 3 RESULTS

### 3.1 Polyploidy

**Figure 3:**
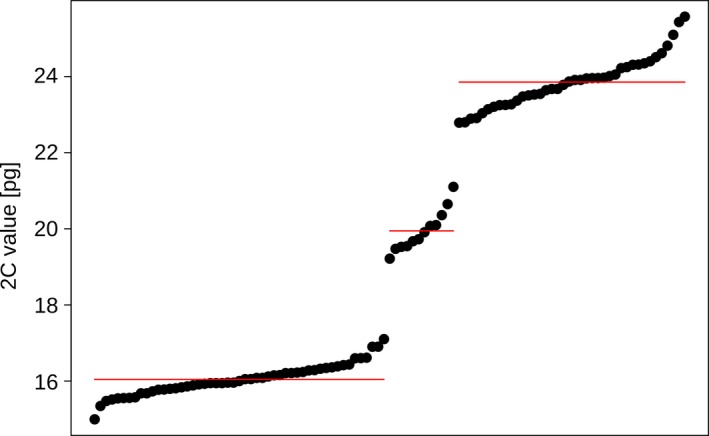
Flow cytometric genome size measurements of individuals of *Rutidosis lanata* showing three clearly distinct size classes. Lines indicate averages for each size class

**Figure 4:**
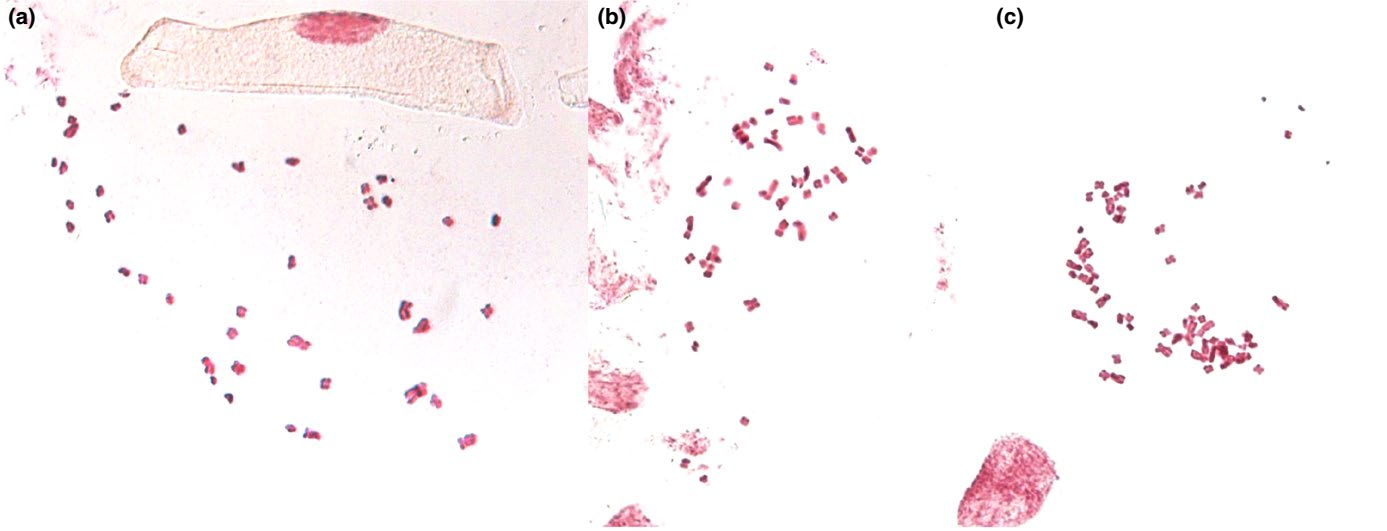
Stained chromosomes of representative (a) tetraploid, (b) pentaploid, and (c) hexaploid individuals of *Rutidosis lanata* (study plants 33 of population Little 1, 74 of Campbell 5, and 33 of Little 1, respectively)

Study plants showed three clearly distinct genome size classes of c. 15.0–17.1 pg, 19.2–21.1 pg, and 22.8–25.6 pg (Figure 3; Data S1). The averages of the size classes (16.0 pg, 19.9 pg, 23.9 pg) showed a ratio of 1.00: 1.24: 1.49, suggesting the existence of four, five, and six genome copies (tetraploidy, pentaploidy, and hexaploidy). This observation was confirmed by mitotic chromosome counts of c. 48 for representative plants in the smallest genome size class (#72, Little 1, #94, Campbell 4), c. 60 for plants in the intermediate class (#74, Campbell 5; #80, Campbell 4), and c. 72 for plants in the largest class (#16, Chaplin 2; #33, Little 1) (Figure 4).

**Figure 5:**
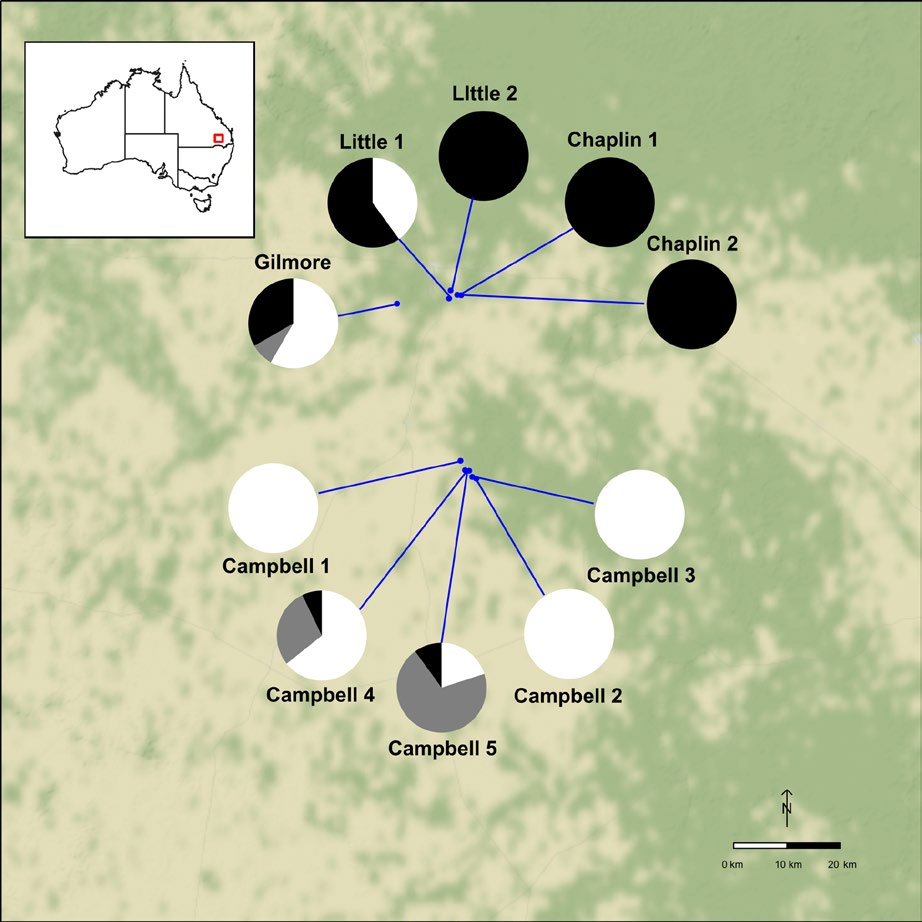
Geographic distribution of ploidy levels found in *Rutidosis lanata* in the study area. Black indicates hexaploids, gray pentaploids, and white tetraploids

Six of the ten study populations exhibited a single ploidy level, and four were mixed (Data S1). Of the latter, three contained at least one pentaploid. In one (Campbell 5), pentaploids were in the majority among the sampled plants. The distribution of ploidy levels in *Rutidosis lanata* showed a clear geographic structure across the sampled area, with hexaploid populations predominantly in the north, and tetraploid populations predominantly in the south (Figure 5).

### 3.2 Crossing experiments

Results of crossing experiments are summarized in Data S2. All plants except one (#105, Gilmore) were unable to set any significant amount of seed after being self‐pollinated, confirming the existence of a self‐incompatibility mechanism in *Rutidosis lanata*. Diallels summarizing pair‐wise crossing success were asymmetric (Data S3), suggesting that the self‐incompatibility mechanism is sporophytic, as in many other Asteraceae (Hiscock & Tabah, 2003).

Mate availability varied across populations with a maximum of 76.4% in Chaplin 1 and a minimum of 48.6% among the tetraploids of Campbell 4 (Table 1). Most populations showed values in the range of 50%–70%, suggesting that mate limitation may be a frequent phenomenon in wild populations of *Rutidosis lanata*.

**Figure 6:**
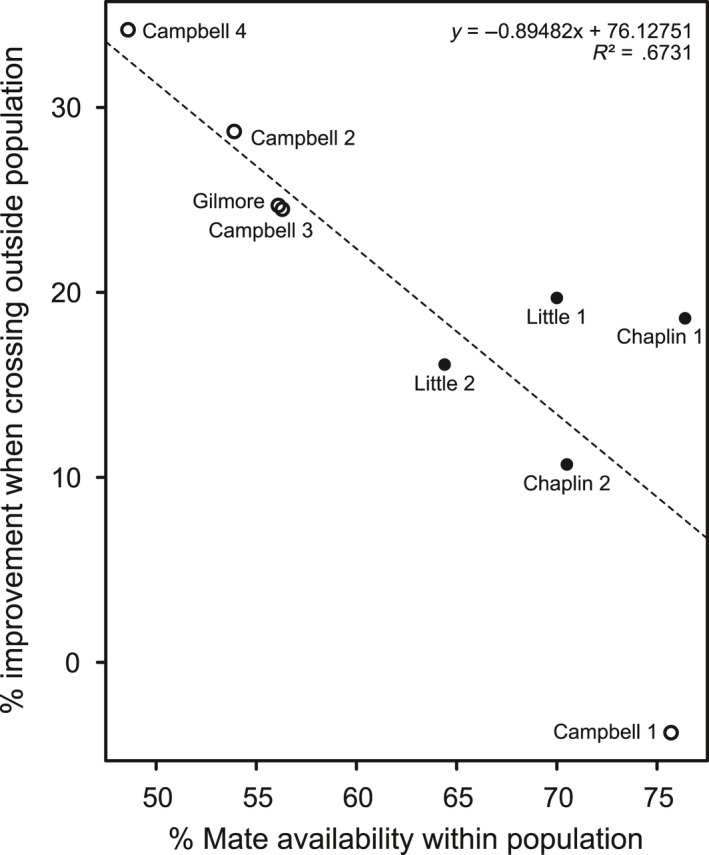
Scatter plot of experimental mating success within nine study populations and improvement of mating success when crossing the population with others. Empty circles indicate tetraploids, black circles hexaploids

Crossing plants at the same ploidy level between populations improved mate availability significantly for most populations (Table 1). Only one population, Campbell 1, did not show any improvement. The benefits of cross‐population pollination were greatest for the most mate limited populations (Figure 6).

**Figure 7:**
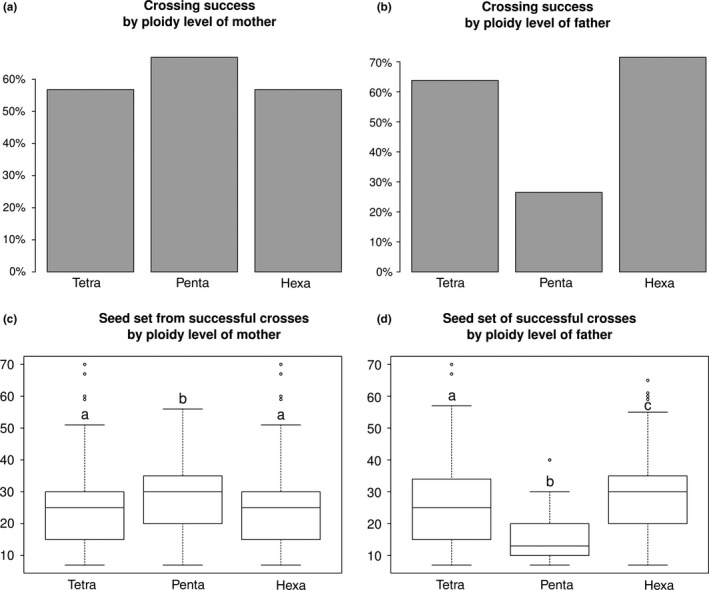
Reproductive fitness of different ploidy levels in *Rutidosis lanata*. (a) success of crosses by ploidy level of mother, (b) by ploidy level of father; (c) seed set of successful crosses by ploidy level of mother, (d) by ploidy level of father. Different lower‐case letters indicate significant difference in Welch two‐sample t tests

Crosses between tetraploid and hexaploid plants were highly successful (83.5%, with mean seed set of successful crosses of 28.5, *n* = 236), indicating that pentaploids could be produced readily in the wild where the two even ploidy levels come into contact. The availability of twelve naturally occurring pentaploids among our study plants allowed us to explore their reproductive fitness by including them in the crossing experiments. Pentaploids did not suffer in their role as pollen recipients (mothers), experiencing slightly higher pollination success (Figure 7a) and producing similar numbers of seeds in successfully pollinated seed heads as tetraploids and hexaploids (Figure 7c). In their role as pollen donors (fathers), however, they were able to pollinate only less than half as many flower heads as the even‐numbered ploidy levels (Figure 7b), and even the heads counted as successfully pollinated by pentaploids set only c. 40%–50% as many fertile seeds as those pollinated by other plants (Figure 7d).

## 4 DISCUSSION

### 4.1 *Rutidosis lanata* is self‐incompatible

Our results indicate that *Rutidosis lanata* has a sporophytic self‐incompatibility system; plants generally cannot self‐pollinate and in addition reject pollen of closely related plants. This means that populations have to be genetically diverse if long‐term reproductive success is to be ensured (Young & Pickup, 2010). Because even purely stochastic processes will lead to a loss of diversity in S‐alleles in small and fragmented populations, populations need to be large enough to maintain the necessary diversity to be sustainable.

Several study populations showed evidence of significant mate limitation, that is, individual plants would reject a large percentage of the pollen they receive from other members of their population. This suggests that a lack of S‐allele diversity may explain or at least contribute toward low set seed in natural populations and harvested plants (Young & Pickup, 2010).

Increased fertility in crosses between plants from different populations suggests that there is among‐population diversity in S alleles, and mixing plants from different sources (but with the same chromosome number, see below) should improve seed set and thus the long‐term viability of translocated or restored populations (Pickup & Young, 2007).

### 4.2 *Rutidosis lanata* is a polyploid complex

Our study has demonstrated that *Rutidosis lanata* constitutes a polyploid complex. Tetraploid, pentaploid, and hexaploid individuals coexist within the species. It can be assumed that the rare pentaploids are hybrids of the more frequent tetraploids and hexaploids. Six of the ten study populations appeared to be either purely tetraploid or purely hexaploid, while four populations showed some naturally occurring admixture, with three of them containing at least one pentaploid hybrid.

Despite this, the distribution of cytotypes showed a clear geographic structure, with predominantly hexaploid populations in the north of the study area and predominantly tetraploid populations in the south. This suggests that mixing of populations or seeds during translocation or genetic rescue has the potential to produce even more pentaploid intermediates than exist naturally, especially when managing populations whose genome copy numbers are unknown (Severns & Liston, 2008).

No diploids (two genome copies) were observed in the study, but they must have existed at least historically, as the hexaploid cytotype cannot arise from genome duplication alone; it would have come about through hybridization either of tetraploids with octoploids or of diploids with tetraploids followed by genome duplication. Study populations covered only part of the range of *Rutidosis lanata*, and it is consequently possible that additional undiscovered diversity in cytotypes exists in other areas.

### 4.3 Pentaploids show reduced reproductive fitness

Results of the crossing experiment demonstrated a significantly reduced reproductive fitness in the male function of pentaploid hybrids compared to the parental, even‐numbered ploidy levels. They pollinated successfully at less than half the normal rate, and even pollination events that were counted as successful produced less than half as many seeds as when the father was even‐ploid.

As crossing success and seed set are subsequent hurdles to be overcome, their effects are multiplicative. This means that any additionally produced pentaploid in a mixed population would on average represent an at least 75% loss to reproductive success compared to a nonpentaploid plant. Existence of pentaploids thus can be expected to compound the effect of mate limitation through reduced genetic diversity, further contributing to reduced seed set and lower population viability.

Interestingly, there was no negative effect of pentaploidy on the female function, a result that was unexpected because pentaploids in other plant species have been observed to be sterile (Bringhurst & Khan, 1963). In rare cases, hybrid odd‐ploids have been found that were fertile, but they were described as having comparable levels of female and male fertility (Avers, 1954; Pirrie & Power, 1986), or else they constituted entire species that have transitioned to asexual reproduction (Harvey & Braggins, 1985). Examination of the chromosome behavior of pentaploid *R. lanata* during meiosis would be required to understand the basis of their female fertility despite male near‐sterility.

### 4.4 Management recommendations

Several of the study populations benefited from interpopulation crossing leading to higher mate availability and thus higher seed set. Conversely, crosses between populations with different genome copy numbers should be avoided because the resulting intermediates are at least partly male sterile and thus exacerbate the problems of mate limitation and lower seed set. Given the relatively small geographic range of the species, it is not expected that population mixing would produce adverse effects due to outbreeding depression resulting from local adaptation.

Accordingly, to maximize seed set and population viability, existing populations could be augmented with plants from other, especially larger populations with the same ploidy level. When creating new populations, seed provenances with the same ploidy level should be mixed, but populations with different ploidy levels should be kept spatially separated.

### 4.5 General applicability

Polyploidy is a widespread phenomenon among vascular plants (Leitch & Bennett, 1997). It has been suggested that in flowering plants infraspecific polyploidisation may be more common than allopolyploidy (Ramsey & Schemske, 1998). This means that in the absence of genetic data conservation actions such as seed orcharding, translocation, or genetic rescue carry the risk of mixing populations with different ploidy levels, potentially producing numerous reproductively unfit hybrids and reducing population viability (Severns & Liston, 2008).

Of particular, concern are species from groups known for frequent polyploidisation such as Asteraceae (daisies), Poaceae (grasses), or ferns. In cases where genetic rescue seems indicated, be it due to mate limitation or inbreeding depression, it would be beneficial to screen managed populations for cytological variability (Severns et al., 2013). In many cases, ploidy levels can be inferred efficiently with flow cytometry, as long as fresh leaf material is available (Doležel et al., 2007).

### 4.6 Population viability analysis of polyploids

The present study has examined mate availability and inferred approximate S‐allele numbers for nine study populations. Ecological models are available that allow predictions to be made about the number of individuals and the number of S‐alleles needed to establish a sustainable population of self‐incompatible plants (often defined as genetically viable for at least 100 years even in the absence of immigration) (Thrall, Encinas‐Viso, Hoebee, & Young, 2014). However, even the most appropriate models were developed for diploid species, not for polyploids such as *Rutidosis lanata*, and most commonly used models do not consider genetics at all.

Assuming that the S‐locus is functionally polyploid, polyploids are faced with a trade‐off: due to the higher number of genome copies per plant they can maintain higher numbers of S‐alleles in a population of the same size than diploids, but on the other hand, they also need higher numbers because a pollen recipient will test pollen for a larger number of S‐alleles, making it less likely that pollen is accepted. Some empirical studies have suggested that tetraploids may be more mate‐limited than diploids of the same species (Brown & Young, 2000; Pickup & Young, 2007), but in *R. lanata* the higher ploidy level populations generally showed higher mate availability (64.4%–76.4% vs. 48.6%–75.7%, Table 1). With limited data available and in the absence of an explicit model‐based study, it is unclear which effect will be stronger, and if polyploids need larger or smaller population sizes than diploids to achieve long‐term viability. This also means that even researchers used to intuiting the genetic health of a population sample from the estimated number of S‐alleles will find it hard to transfer their experience to polyploids.

Expanding the relevant population models to apply to polyploid species would be beneficial beyond the present study species, as polyploidy is common across the land plants (Leitch & Bennett, 1997). Once developed, these models could inform management decisions for many different species given knowledge of their ploidy level and breeding system.

## CONFLICTS OF INTEREST

None declared.

## AUTHOR CONTRIBUTIONS

ANSL conducted experiments, analyzed data, and prepared manuscript; DJM conducted experiments and provided comments on manuscript; BD conducted field work and provided study plants and comments on manuscript; AGY conceived project and contributed to analysis and manuscript preparation.

## DATA ACCESSIBILITY

All underlying data are available as supplementary data.
