# Supplementary material for: Genetic rescue in a plant polyploid complex: Case study on the importance of genetic and trait data for conservation management
Source: Ecol Evol. 2018 Apr 25;8(10):5153–63. doi: 10.1002/ece3.4039 (PMC5980434; doi:10.1002/ece3.4039)
Supplement: Supplementary file 1 [file ECE3-8-5153-s001.docx]

**Supplementary Data 1.** Genome size measurements for individuals of *Rutidosis lanata* from ten populations.

| **Population** | **Individual** | **2C value [pg]** | **Ploidy level** |
| --- | --- | --- | --- |
| Campbell1 | 10 | 15.84 | tetraploid |
| Campbell1 | 25 | 16.39 | tetraploid |
| Campbell1 | 57 | 16.60 | tetraploid |
| Campbell1 | 77 | 17.10 | tetraploid |
| Campbell1 | 78 | 16.22 | tetraploid |
| Campbell1 | 86 | 16.15 | tetraploid |
| Campbell1 | 119 | 15.55 | tetraploid |
| Campbell2 | 4 | 15.48 | tetraploid |
| Campbell2 | 9 | 15.95 | tetraploid |
| Campbell2 | 21 | 15.95 | tetraploid |
| Campbell2 | 26 | 15.80 | tetraploid |
| Campbell2 | 30 | 16.60 | tetraploid |
| Campbell2 | 36 | 15.78 | tetraploid |
| Campbell2 | 93 | 16.05 | tetraploid |
| Campbell2 | 96 | 16.42 | tetraploid |
| Campbell2 | 99 | 15.57 | tetraploid |
| Campbell2 | 124 | 16.21 | tetraploid |
| Campbell3 | 27 | 15.86 | tetraploid |
| Campbell3 | 48 | 16.24 | tetraploid |
| Campbell3 | 56 | 16.05 | tetraploid |
| Campbell3 | 58 | 15.96 | tetraploid |
| Campbell3 | 81 | 15.91 | tetraploid |
| Campbell3 | 87 | 15.68 | tetraploid |
| Campbell3 | 88 | 16.08 | tetraploid |
| Campbell3 | 92 | 16.21 | tetraploid |
| Campbell3 | 98 | 15.52 | tetraploid |
| Campbell3 | 108 | 16.00 | tetraploid |
| Campbell3 | 109 | 15.81 | tetraploid |
| Campbell3 | 121 | 15.77 | tetraploid |
| Campbell4 | 13 | 14.99 | tetraploid |
| Campbell4 | 14 | 15.93 | tetraploid |
| Campbell4 | 20 | 19.55 | pentaploid |
| Campbell4 | 28 | 15.35 | tetraploid |
| Campbell4 | 31 | 15.56 | tetraploid |
| Campbell4 | 40 | 19.73 | pentaploid |
| Campbell4 | 42 | 20.36 | pentaploid |
| Campbell4 | 50 | 15.95 | tetraploid |
| Campbell4 | 61 | 16.90 | tetraploid |
| Campbell4 | 73 | 16.28 | tetraploid |
| Campbell4 | 79 | 16.44 | tetraploid |
| Campbell4 | 80 | 21.10 | pentaploid |
| Campbell4 | 94 | 16.32 | tetraploid |
| Campbell4 | 95 | 24.51 | hexaploid |
| Campbell5 | 6 | 20.65 | pentaploid |
| Campbell5 | 11 | 19.67 | pentaploid |
| Campbell5 | 18 | 20.10 | pentaploid |
| Campbell5 | 38 | 19.53 | pentaploid |
| Campbell5 | 62 | 16.34 | tetraploid |
| Campbell5 | 64 | 16.90 | tetraploid |
| Campbell5 | 71 | 20.08 | pentaploid |
| Campbell5 | 74 | 19.22 | pentaploid |
| Campbell5 | 100 | 19.47 | pentaploid |
| Campbell5 | 106 | 23.95 | hexaploid |
| Chaplin1 | 1 | 24.61 | hexaploid |
| Chaplin1 | 5 | 24.32 | hexaploid |
| Chaplin1 | 41 | 24.81 | hexaploid |
| Chaplin1 | 54 | 23.91 | hexaploid |
| Chaplin1 | 67 | 24.25 | hexaploid |
| Chaplin1 | 89 | 24.40 | hexaploid |
| Chaplin1 | 91 | 23.91 | hexaploid |
| Chaplin1 | 107 | 23.87 | hexaploid |
| Chaplin1 | 122 | 22.79 | hexaploid |
| Chaplin2 | 16 | 23.68 | hexaploid |
| Chaplin2 | 19 | 23.26 | hexaploid |
| Chaplin2 | 32 | 23.68 | hexaploid |
| Chaplin2 | 49 | 22.80 | hexaploid |
| Chaplin2 | 66 | 22.91 | hexaploid |
| Chaplin2 | 69 | 24.01 | hexaploid |
| Chaplin2 | 104 | 23.51 | hexaploid |
| Chaplin2 | 113 | 25.58 | hexaploid |
| Chaplin2 | 117 | 25.43 | hexaploid |
| Gilmore | 2 | 19.91 | pentaploid |
| Gilmore | 29 | 16.61 | tetraploid |
| Gilmore | 39 | 16.36 | tetraploid |
| Gilmore | 45 | 16.16 | tetraploid |
| Gilmore | 46 | 16.29 | tetraploid |
| Gilmore | 51 | 23.48 | hexaploid |
| Gilmore | 52 | 24.05 | hexaploid |
| Gilmore | 83 | 23.96 | hexaploid |
| Gilmore | 97 | 16.12 | tetraploid |
| Gilmore | 103 | 23.78 | hexaploid |
| Gilmore | 105 | 15.68 | tetraploid |
| Gilmore | 114 | 16.08 | tetraploid |
| Little1 | 17 | 15.73 | tetraploid |
| Little1 | 33 | 25.10 | hexaploid |
| Little1 | 37 | 23.04 | hexaploid |
| Little1 | 47 | 15.55 | tetraploid |
| Little1 | 53 | 23.21 | hexaploid |
| Little1 | 72 | 15.96 | tetraploid |
| Little1 | 110 | 23.96 | hexaploid |
| Little1 | 116 | 23.97 | hexaploid |
| Little1 | 120 | 15.89 | tetraploid |
| Little2 | 7 | 24.35 | hexaploid |
| Little2 | 12 | 23.25 | hexaploid |
| Little2 | 22 | 24.31 | hexaploid |
| Little2 | 34 | 23.14 | hexaploid |
| Little2 | 63 | 22.90 | hexaploid |
| Little2 | 65 | 23.53 | hexaploid |
| Little2 | 90 | 23.37 | hexaploid |
| Little2 | 102 | 23.27 | hexaploid |
| Little2 | 111 | 23.55 | hexaploid |
| Little2 | 115 | 23.64 | hexaploid |
| Little2 | 118 | 24.22 | hexaploid |
