# Supplementary material for: Genetic rescue in a plant polyploid complex: Case study on the importance of genetic and trait data for conservation management
Source: Ecol Evol. 2018 Apr 25;8(10):5153–63. doi: 10.1002/ece3.4039 (PMC5980434; doi:10.1002/ece3.4039)
Supplement: Supplementary file 2 [file ECE3-8-5153-s002.docx]

**Supplementary data 2.** Crossing results for individuals of the rare Australian daisy Rutidosis lanata.

| **Mother plant** | **Father plant** | **Fertile seeds** | **Population mother** | **Population father** | **Ploidy mother** | **Ploidy father** |
| --- | --- | --- | --- | --- | --- | --- |
| 1 | 1 | 0 | Chaplin1 | Chaplin1 | Hexa | Hexa |
| 1 | 5 | 20 | Chaplin1 | Chaplin1 | Hexa | Hexa |
| 1 | 41 | 1 | Chaplin1 | Chaplin1 | Hexa | Hexa |
| 1 | 54 | 1 | Chaplin1 | Chaplin1 | Hexa | Hexa |
| 1 | 67 | 25 | Chaplin1 | Chaplin1 | Hexa | Hexa |
| 1 | 89 | 0 | Chaplin1 | Chaplin1 | Hexa | Hexa |
| 1 | 91 | 20 | Chaplin1 | Chaplin1 | Hexa | Hexa |
| 1 | 107 | 30 | Chaplin1 | Chaplin1 | Hexa | Hexa |
| 1 | 122 | 5 | Chaplin1 | Chaplin1 | Hexa | Hexa |
| 2 | 2 | 0 | Gilmore | Gilmore | Penta | Penta |
| 2 | 29 | 35 | Gilmore | Gilmore | Penta | Tetra |
| 2 | 39 | 40 | Gilmore | Gilmore | Penta | Tetra |
| 2 | 41 | 35 | Gilmore | Chaplin1 | Penta | Hexa |
| 2 | 45 | 20 | Gilmore | Gilmore | Penta | Tetra |
| 2 | 45 | 35 | Gilmore | Gilmore | Penta | Tetra |
| 2 | 46 | 30 | Gilmore | Gilmore | Penta | Tetra |
| 2 | 46 | 30 | Gilmore | Gilmore | Penta | Tetra |
| 2 | 50 | 25 | Gilmore | Campbell4 | Penta | Tetra |
| 2 | 62 | 12 | Gilmore | Campbell5 | Penta | Tetra |
| 2 | 63 | 25 | Gilmore | Little2 | Penta | Hexa |
| 2 | 66 | 21 | Gilmore | Chaplin2 | Penta | Hexa |
| 2 | 83 | 10 | Gilmore | Gilmore | Penta | Hexa |
| 2 | 91 | 40 | Gilmore | Chaplin1 | Penta | Hexa |
| 2 | 97 | 20 | Gilmore | Gilmore | Penta | Tetra |
| 2 | 103 | 25 | Gilmore | Gilmore | Penta | Hexa |
| 2 | 105 | 30 | Gilmore | Gilmore | Penta | Tetra |
| 2 | 109 | 15 | Gilmore | Campbell3 | Penta | Tetra |
| 2 | 114 | 40 | Gilmore | Gilmore | Penta | Tetra |
| 2 | 124 | 25 | Gilmore | Campbell2 | Penta | Tetra |
| 4 | 4 | 0 | Campbell2 | Campbell2 | Tetra | Tetra |
| 4 | 4 | 0 | Campbell2 | Campbell2 | Tetra | Tetra |
| 4 | 9 | 12 | Campbell2 | Campbell2 | Tetra | Tetra |
| 4 | 21 | 20 | Campbell2 | Campbell2 | Tetra | Tetra |
| 4 | 26 | 20 | Campbell2 | Campbell2 | Tetra | Tetra |
| 4 | 30 | 8 | Campbell2 | Campbell2 | Tetra | Tetra |
| 4 | 36 | 5 | Campbell2 | Campbell2 | Tetra | Tetra |
| 4 | 46 | 30 | Campbell2 | Gilmore | Tetra | Tetra |
| 4 | 93 | 6 | Campbell2 | Campbell2 | Tetra | Tetra |
| 4 | 96 | 30 | Campbell2 | Campbell2 | Tetra | Tetra |
| 4 | 96 | 30 | Campbell2 | Campbell2 | Tetra | Tetra |
| 5 | 1 | 15 | Chaplin1 | Chaplin1 | Hexa | Hexa |
| 5 | 17 | 0 | Chaplin1 | Little1 | Hexa | Tetra |
| 5 | 41 | 34 | Chaplin1 | Chaplin1 | Hexa | Hexa |
| 5 | 54 | 30 | Chaplin1 | Chaplin1 | Hexa | Hexa |
| 5 | 67 | 20 | Chaplin1 | Chaplin1 | Hexa | Hexa |
| 5 | 89 | 0 | Chaplin1 | Chaplin1 | Hexa | Hexa |
| 5 | 91 | 10 | Chaplin1 | Chaplin1 | Hexa | Hexa |
| 5 | 107 | 15 | Chaplin1 | Chaplin1 | Hexa | Hexa |
| 5 | 122 | 17 | Chaplin1 | Chaplin1 | Hexa | Hexa |
| 6 | 6 | 0 | Campbell5 | Campbell5 | Penta | Penta |
| 6 | 6 | 0 | Campbell5 | Campbell5 | Penta | Penta |
| 6 | 11 | 0 | Campbell5 | Campbell5 | Penta | Penta |
| 6 | 11 | 8 | Campbell5 | Campbell5 | Penta | Penta |
| 6 | 18 | 0 | Campbell5 | Campbell5 | Penta | Penta |
| 6 | 18 | 0 | Campbell5 | Campbell5 | Penta | Penta |
| 6 | 35 | 54 | Campbell5 | Campbell5 | Penta | ? |
| 6 | 38 | 0 | Campbell5 | Campbell5 | Penta | Penta |
| 6 | 51 | 30 | Campbell5 | Gilmore | Penta | Hexa |
| 6 | 62 | 30 | Campbell5 | Campbell5 | Penta | Tetra |
| 6 | 64 | 25 | Campbell5 | Campbell5 | Penta | Tetra |
| 6 | 71 | 0 | Campbell5 | Campbell5 | Penta | Penta |
| 6 | 71 | 0 | Campbell5 | Campbell5 | Penta | Penta |
| 6 | 74 | 0 | Campbell5 | Campbell5 | Penta | Penta |
| 6 | 100 | 0 | Campbell5 | Campbell5 | Penta | Penta |
| 6 | 101 | 0 | Campbell5 | Campbell5 | Penta | ? |
| 6 | 101 | 20 | Campbell5 | Campbell5 | Penta | ? |
| 6 | 105 | 35 | Campbell5 | Gilmore | Penta | Tetra |
| 7 | 7 | 0 | Little2 | Little2 | Hexa | Hexa |
| 7 | 7 | 1 | Little2 | Little2 | Hexa | Hexa |
| 7 | 10 | 30 | Little2 | Campbell1 | Hexa | Tetra |
| 7 | 11 | 0 | Little2 | Campbell5 | Hexa | Penta |
| 7 | 12 | 30 | Little2 | Little2 | Hexa | Hexa |
| 7 | 17 | 7 | Little2 | Little1 | Hexa | Tetra |
| 7 | 22 | 1 | Little2 | Little2 | Hexa | Hexa |
| 7 | 33 | 0 | Little2 | Little1 | Hexa | Hexa |
| 7 | 34 | 29 | Little2 | Little2 | Hexa | Hexa |
| 7 | 63 | 21 | Little2 | Little2 | Hexa | Hexa |
| 7 | 63 | 0 | Little2 | Little2 | Hexa | Hexa |
| 7 | 65 | 30 | Little2 | Little2 | Hexa | Hexa |
| 7 | 74 | 0 | Little2 | Campbell5 | Hexa | Penta |
| 7 | 88 | 10 | Little2 | Campbell3 | Hexa | Tetra |
| 7 | 88 | 10 | Little2 | Campbell3 | Hexa | Tetra |
| 7 | 90 | 35 | Little2 | Little2 | Hexa | Hexa |
| 7 | 102 | 30 | Little2 | Little2 | Hexa | Hexa |
| 7 | 107 | 40 | Little2 | Chaplin1 | Hexa | Hexa |
| 7 | 110 | 25 | Little2 | Little1 | Hexa | Hexa |
| 7 | 111 | 35 | Little2 | Little2 | Hexa | Hexa |
| 7 | 113 | 15 | Little2 | Chaplin2 | Hexa | Hexa |
| 7 | 115 | 1 | Little2 | Little2 | Hexa | Hexa |
| 7 | 118 | 0 | Little2 | Little2 | Hexa | Hexa |
| 7 | 120 | 57 | Little2 | Little1 | Hexa | Tetra |
| 7 | 123 | 30 | Little2 | Little1 | Hexa | NA |
| 9 | 4 | 10 | Campbell2 | Campbell2 | Tetra | Tetra |
| 9 | 9 | 0 | Campbell2 | Campbell2 | Tetra | Tetra |
| 9 | 9 | 0 | Campbell2 | Campbell2 | Tetra | Tetra |
| 9 | 21 | 10 | Campbell2 | Campbell2 | Tetra | Tetra |
| 9 | 25 | 1 | Campbell2 | Campbell1 | Tetra | Tetra |
| 9 | 26 | 13 | Campbell2 | Campbell2 | Tetra | Tetra |
| 9 | 30 | 40 | Campbell2 | Campbell2 | Tetra | Tetra |
| 9 | 36 | 2 | Campbell2 | Campbell2 | Tetra | Tetra |
| 9 | 73 | 30 | Campbell2 | Campbell4 | Tetra | Tetra |
| 9 | 82 | 0 | Campbell2 | Campbell2 | Tetra | NA |
| 9 | 93 | 15 | Campbell2 | Campbell2 | Tetra | Tetra |
| 9 | 96 | 30 | Campbell2 | Campbell2 | Tetra | Tetra |
| 9 | 99 | 7 | Campbell2 | Campbell2 | Tetra | Tetra |
| 9 | 122 | 30 | Campbell2 | Chaplin1 | Tetra | Hexa |
| 9 | 124 | 14 | Campbell2 | Campbell2 | Tetra | Tetra |
| 10 | 7 | 7 | Campbell1 | Little2 | Tetra | Hexa |
| 10 | 10 | 0 | Campbell1 | Campbell1 | Tetra | Tetra |
| 10 | 10 | 0 | Campbell1 | Campbell1 | Tetra | Tetra |
| 10 | 25 | 1 | Campbell1 | Campbell1 | Tetra | Tetra |
| 10 | 45 | 20 | Campbell1 | Gilmore | Tetra | Tetra |
| 10 | 57 | 20 | Campbell1 | Campbell1 | Tetra | Tetra |
| 10 | 77 | 4 | Campbell1 | Campbell1 | Tetra | Tetra |
| 10 | 78 | 35 | Campbell1 | Campbell1 | Tetra | Tetra |
| 10 | 86 | 0 | Campbell1 | Campbell1 | Tetra | Tetra |
| 10 | 88 | 25 | Campbell1 | Campbell3 | Tetra | Tetra |
| 10 | 119 | 15 | Campbell1 | Campbell1 | Tetra | Tetra |
| 11 | 6 | 8 | Campbell5 | Campbell5 | Penta | Penta |
| 11 | 6 | 30 | Campbell5 | Campbell5 | Penta | Penta |
| 11 | 7 | 35 | Campbell5 | Little2 | Penta | Hexa |
| 11 | 11 | 1 | Campbell5 | Campbell5 | Penta | Penta |
| 11 | 11 | 0 | Campbell5 | Campbell5 | Penta | Penta |
| 11 | 11 | 0 | Campbell5 | Campbell5 | Penta | Penta |
| 11 | 11 | 0 | Campbell5 | Campbell5 | Penta | Penta |
| 11 | 11 | 0 | Campbell5 | Campbell5 | Penta | Penta |
| 11 | 11 | 0 | Campbell5 | Campbell5 | Penta | Penta |
| 11 | 18 | 0 | Campbell5 | Campbell5 | Penta | Penta |
| 11 | 18 | 0 | Campbell5 | Campbell5 | Penta | Penta |
| 11 | 18 | 0 | Campbell5 | Campbell5 | Penta | Penta |
| 11 | 35 | 50 | Campbell5 | Campbell5 | Penta | ? |
| 11 | 38 | 12 | Campbell5 | Campbell5 | Penta | Penta |
| 11 | 41 | 13 | Campbell5 | Chaplin1 | Penta | Hexa |
| 11 | 51 | 30 | Campbell5 | Gilmore | Penta | Hexa |
| 11 | 62 | 30 | Campbell5 | Campbell5 | Penta | Tetra |
| 11 | 64 | 25 | Campbell5 | Campbell5 | Penta | Tetra |
| 11 | 71 | 0 | Campbell5 | Campbell5 | Penta | Penta |
| 11 | 71 | 0 | Campbell5 | Campbell5 | Penta | Penta |
| 11 | 74 | 0 | Campbell5 | Campbell5 | Penta | Penta |
| 11 | 74 | 0 | Campbell5 | Campbell5 | Penta | Penta |
| 11 | 91 | 25 | Campbell5 | Chaplin1 | Penta | Hexa |
| 11 | 96 | 56 | Campbell5 | Campbell2 | Penta | Tetra |
| 11 | 100 | 0 | Campbell5 | Campbell5 | Penta | Penta |
| 11 | 101 | 40 | Campbell5 | Campbell5 | Penta | ? |
| 11 | 101 | 10 | Campbell5 | Campbell5 | Penta | ? |
| 11 | 106 | 35 | Campbell5 | Campbell5 | Penta | Hexa |
| 11 | 116 | 38 | Campbell5 | Little1 | Penta | Hexa |
| 11 | 118 | 55 | Campbell5 | Little2 | Penta | Hexa |
| 12 | 7 | 30 | Little2 | Little2 | Hexa | Hexa |
| 12 | 12 | 0 | Little2 | Little2 | Hexa | Hexa |
| 12 | 12 | 0 | Little2 | Little2 | Hexa | Hexa |
| 12 | 22 | 35 | Little2 | Little2 | Hexa | Hexa |
| 12 | 34 | 20 | Little2 | Little2 | Hexa | Hexa |
| 12 | 63 | 20 | Little2 | Little2 | Hexa | Hexa |
| 12 | 65 | 20 | Little2 | Little2 | Hexa | Hexa |
| 12 | 65 | 2 | Little2 | Little2 | Hexa | Hexa |
| 12 | 90 | 25 | Little2 | Little2 | Hexa | Hexa |
| 12 | 102 | 3 | Little2 | Little2 | Hexa | Hexa |
| 12 | 111 | 35 | Little2 | Little2 | Hexa | Hexa |
| 12 | 115 | 0 | Little2 | Little2 | Hexa | Hexa |
| 12 | 118 | 13 | Little2 | Little2 | Hexa | Hexa |
| 13 | 13 | 15 | Campbell4 | Campbell4 | Tetra | Tetra |
| 13 | 13 | 0 | Campbell4 | Campbell4 | Tetra | Tetra |
| 13 | 13 | 0 | Campbell4 | Campbell4 | Tetra | Tetra |
| 13 | 13 | 8 | Campbell4 | Campbell4 | Tetra | Tetra |
| 13 | 14 | 0 | Campbell4 | Campbell4 | Tetra | Tetra |
| 13 | 14 | 0 | Campbell4 | Campbell4 | Tetra | Tetra |
| 13 | 20 | 20 | Campbell4 | Campbell4 | Tetra | Penta |
| 13 | 28 | 0 | Campbell4 | Campbell4 | Tetra | Tetra |
| 13 | 31 | 3 | Campbell4 | Campbell4 | Tetra | Tetra |
| 13 | 31 | 0 | Campbell4 | Campbell4 | Tetra | Tetra |
| 13 | 40 | 30 | Campbell4 | Campbell4 | Tetra | Penta |
| 13 | 40 | 2 | Campbell4 | Campbell4 | Tetra | Penta |
| 13 | 42 | 3 | Campbell4 | Campbell4 | Tetra | Penta |
| 13 | 50 | 0 | Campbell4 | Campbell4 | Tetra | Tetra |
| 13 | 61 | 3 | Campbell4 | Campbell4 | Tetra | Tetra |
| 13 | 67 | 30 | Campbell4 | Chaplin1 | Tetra | Hexa |
| 13 | 73 | 40 | Campbell4 | Campbell4 | Tetra | Tetra |
| 13 | 79 | 10 | Campbell4 | Campbell4 | Tetra | Tetra |
| 13 | 80 | 2 | Campbell4 | Campbell4 | Tetra | Penta |
| 13 | 94 | 25 | Campbell4 | Campbell4 | Tetra | Tetra |
| 13 | 95 | 30 | Campbell4 | Campbell4 | Tetra | Hexa |
| 13 | 95 | 20 | Campbell4 | Campbell4 | Tetra | Hexa |
| 14 | 2 | 16 | Campbell4 | Gilmore | Tetra | Penta |
| 14 | 13 | 4 | Campbell4 | Campbell4 | Tetra | Tetra |
| 14 | 13 | 0 | Campbell4 | Campbell4 | Tetra | Tetra |
| 14 | 14 | 10 | Campbell4 | Campbell4 | Tetra | Tetra |
| 14 | 14 | 0 | Campbell4 | Campbell4 | Tetra | Tetra |
| 14 | 14 | 0 | Campbell4 | Campbell4 | Tetra | Tetra |
| 14 | 14 | 0 | Campbell4 | Campbell4 | Tetra | Tetra |
| 14 | 14 | 0 | Campbell4 | Campbell4 | Tetra | Tetra |
| 14 | 14 | 0 | Campbell4 | Campbell4 | Tetra | Tetra |
| 14 | 14 | 0 | Campbell4 | Campbell4 | Tetra | Tetra |
| 14 | 20 | 5 | Campbell4 | Campbell4 | Tetra | Penta |
| 14 | 28 | 0 | Campbell4 | Campbell4 | Tetra | Tetra |
| 14 | 31 | 2 | Campbell4 | Campbell4 | Tetra | Tetra |
| 14 | 40 | 40 | Campbell4 | Campbell4 | Tetra | Penta |
| 14 | 41 | 47 | Campbell4 | Chaplin1 | Tetra | Hexa |
| 14 | 42 | 1 | Campbell4 | Campbell4 | Tetra | Penta |
| 14 | 42 | 2 | Campbell4 | Campbell4 | Tetra | Penta |
| 14 | 42 | 0 | Campbell4 | Campbell4 | Tetra | Penta |
| 14 | 42 | 0 | Campbell4 | Campbell4 | Tetra | Penta |
| 14 | 42 | 0 | Campbell4 | Campbell4 | Tetra | Penta |
| 14 | 45 | 40 | Campbell4 | Gilmore | Tetra | Tetra |
| 14 | 50 | 50 | Campbell4 | Campbell4 | Tetra | Tetra |
| 14 | 50 | 40 | Campbell4 | Campbell4 | Tetra | Tetra |
| 14 | 50 | 15 | Campbell4 | Campbell4 | Tetra | Tetra |
| 14 | 50 | 20 | Campbell4 | Campbell4 | Tetra | Tetra |
| 14 | 57 | 3 | Campbell4 | Campbell1 | Tetra | Tetra |
| 14 | 57 | 35 | Campbell4 | Campbell1 | Tetra | Tetra |
| 14 | 61 | 0 | Campbell4 | Campbell4 | Tetra | Tetra |
| 14 | 73 | 35 | Campbell4 | Campbell4 | Tetra | Tetra |
| 14 | 79 | 0 | Campbell4 | Campbell4 | Tetra | Tetra |
| 14 | 80 | 0 | Campbell4 | Campbell4 | Tetra | Penta |
| 14 | 88 | 15 | Campbell4 | Campbell3 | Tetra | Tetra |
| 14 | 94 | 50 | Campbell4 | Campbell4 | Tetra | Tetra |
| 14 | 95 | 20 | Campbell4 | Campbell4 | Tetra | Hexa |
| 14 | 104 | 0 | Campbell4 | Chaplin2 | Tetra | Hexa |
| 14 | 108 | 35 | Campbell4 | Campbell3 | Tetra | Tetra |
| 16 | 16 | 0 | Chaplin2 | Chaplin2 | Hexa | Hexa |
| 16 | 16 | 0 | Chaplin2 | Chaplin2 | Hexa | Hexa |
| 16 | 19 | 0 | Chaplin2 | Chaplin2 | Hexa | Hexa |
| 16 | 32 | 0 | Chaplin2 | Chaplin2 | Hexa | Hexa |
| 16 | 49 | 0 | Chaplin2 | Chaplin2 | Hexa | Hexa |
| 16 | 66 | 15 | Chaplin2 | Chaplin2 | Hexa | Hexa |
| 16 | 69 | 6 | Chaplin2 | Chaplin2 | Hexa | Hexa |
| 16 | 113 | 0 | Chaplin2 | Chaplin2 | Hexa | Hexa |
| 16 | 117 | 13 | Chaplin2 | Chaplin2 | Hexa | Hexa |
| 17 | 5 | 0 | Little1 | Chaplin1 | Tetra | Hexa |
| 17 | 17 | 4 | Little1 | Little1 | Tetra | Tetra |
| 17 | 17 | 0 | Little1 | Little1 | Tetra | Tetra |
| 17 | 22 | 32 | Little1 | Little2 | Tetra | Hexa |
| 17 | 22 | 35 | Little1 | Little2 | Tetra | Hexa |
| 17 | 33 | 25 | Little1 | Little1 | Tetra | Hexa |
| 17 | 35 | 40 | Little1 | Campbell5 | Tetra | ? |
| 17 | 37 | 10 | Little1 | Little1 | Tetra | Hexa |
| 17 | 47 | 0 | Little1 | Little1 | Tetra | Tetra |
| 17 | 52 | 30 | Little1 | Gilmore | Tetra | Hexa |
| 17 | 53 | 40 | Little1 | Little1 | Tetra | Hexa |
| 17 | 72 | 17 | Little1 | Little1 | Tetra | Tetra |
| 17 | 82 | 30 | Little1 | Campbell2 | Tetra | NA |
| 17 | 102 | 10 | Little1 | Little2 | Tetra | Hexa |
| 17 | 102 | 30 | Little1 | Little2 | Tetra | Hexa |
| 17 | 102 | 25 | Little1 | Little2 | Tetra | Hexa |
| 17 | 108 | 40 | Little1 | Campbell3 | Tetra | Tetra |
| 17 | 110 | 50 | Little1 | Little1 | Tetra | Hexa |
| 17 | 116 | 35 | Little1 | Little1 | Tetra | Hexa |
| 17 | 119 | 35 | Little1 | Campbell1 | Tetra | Tetra |
| 17 | 120 | 3 | Little1 | Little1 | Tetra | Tetra |
| 17 | 123 | 8 | Little1 | Little1 | Tetra | NA |
| 18 | 6 | 20 | Campbell5 | Campbell5 | Penta | Penta |
| 18 | 6 | 10 | Campbell5 | Campbell5 | Penta | Penta |
| 18 | 11 | 0 | Campbell5 | Campbell5 | Penta | Penta |
| 18 | 11 | 0 | Campbell5 | Campbell5 | Penta | Penta |
| 18 | 18 | 0 | Campbell5 | Campbell5 | Penta | Penta |
| 18 | 18 | 0 | Campbell5 | Campbell5 | Penta | Penta |
| 18 | 18 | 16 | Campbell5 | Campbell5 | Penta | Penta |
| 18 | 18 | 0 | Campbell5 | Campbell5 | Penta | Penta |
| 18 | 18 | 0 | Campbell5 | Campbell5 | Penta | Penta |
| 18 | 18 | 0 | Campbell5 | Campbell5 | Penta | Penta |
| 18 | 18 | 2 | Campbell5 | Campbell5 | Penta | Penta |
| 18 | 29 | 35 | Campbell5 | Gilmore | Penta | Tetra |
| 18 | 35 | 50 | Campbell5 | Campbell5 | Penta | ? |
| 18 | 35 | 30 | Campbell5 | Campbell5 | Penta | ? |
| 18 | 38 | 10 | Campbell5 | Campbell5 | Penta | Penta |
| 18 | 38 | 10 | Campbell5 | Campbell5 | Penta | Penta |
| 18 | 40 | 20 | Campbell5 | Campbell4 | Penta | Penta |
| 18 | 46 | 30 | Campbell5 | Gilmore | Penta | Tetra |
| 18 | 50 | 25 | Campbell5 | Campbell4 | Penta | Tetra |
| 18 | 62 | 40 | Campbell5 | Campbell5 | Penta | Tetra |
| 18 | 64 | 35 | Campbell5 | Campbell5 | Penta | Tetra |
| 18 | 71 | 0 | Campbell5 | Campbell5 | Penta | Penta |
| 18 | 74 | 0 | Campbell5 | Campbell5 | Penta | Penta |
| 18 | 74 | 0 | Campbell5 | Campbell5 | Penta | Penta |
| 18 | 100 | 0 | Campbell5 | Campbell5 | Penta | Penta |
| 18 | 101 | 25 | Campbell5 | Campbell5 | Penta | ? |
| 18 | 105 | 25 | Campbell5 | Gilmore | Penta | Tetra |
| 18 | 110 | 35 | Campbell5 | Little1 | Penta | Hexa |
| 19 | 16 | 15 | Chaplin2 | Chaplin2 | Hexa | Hexa |
| 19 | 19 | 4 | Chaplin2 | Chaplin2 | Hexa | Hexa |
| 19 | 19 | 1 | Chaplin2 | Chaplin2 | Hexa | Hexa |
| 19 | 32 | 45 | Chaplin2 | Chaplin2 | Hexa | Hexa |
| 19 | 49 | 25 | Chaplin2 | Chaplin2 | Hexa | Hexa |
| 19 | 66 | 35 | Chaplin2 | Chaplin2 | Hexa | Hexa |
| 19 | 69 | 9 | Chaplin2 | Chaplin2 | Hexa | Hexa |
| 19 | 95 | 35 | Chaplin2 | Campbell4 | Hexa | Hexa |
| 19 | 104 | 40 | Chaplin2 | Chaplin2 | Hexa | Hexa |
| 19 | 113 | 0 | Chaplin2 | Chaplin2 | Hexa | Hexa |
| 19 | 117 | 12 | Chaplin2 | Chaplin2 | Hexa | Hexa |
| 20 | 13 | 35 | Campbell4 | Campbell4 | Penta | Tetra |
| 20 | 14 | 12 | Campbell4 | Campbell4 | Penta | Tetra |
| 20 | 20 | 0 | Campbell4 | Campbell4 | Penta | Penta |
| 20 | 20 | 1 | Campbell4 | Campbell4 | Penta | Penta |
| 20 | 20 | 0 | Campbell4 | Campbell4 | Penta | Penta |
| 20 | 28 | 25 | Campbell4 | Campbell4 | Penta | Tetra |
| 20 | 31 | 40 | Campbell4 | Campbell4 | Penta | Tetra |
| 20 | 40 | 15 | Campbell4 | Campbell4 | Penta | Penta |
| 20 | 42 | 0 | Campbell4 | Campbell4 | Penta | Penta |
| 20 | 50 | 30 | Campbell4 | Campbell4 | Penta | Tetra |
| 20 | 61 | 30 | Campbell4 | Campbell4 | Penta | Tetra |
| 20 | 73 | 20 | Campbell4 | Campbell4 | Penta | Tetra |
| 20 | 79 | 20 | Campbell4 | Campbell4 | Penta | Tetra |
| 20 | 80 | 0 | Campbell4 | Campbell4 | Penta | Penta |
| 20 | 94 | 0 | Campbell4 | Campbell4 | Penta | Tetra |
| 20 | 95 | 15 | Campbell4 | Campbell4 | Penta | Hexa |
| 21 | 4 | 15 | Campbell2 | Campbell2 | Tetra | Tetra |
| 21 | 9 | 20 | Campbell2 | Campbell2 | Tetra | Tetra |
| 21 | 21 | 0 | Campbell2 | Campbell2 | Tetra | Tetra |
| 21 | 26 | 3 | Campbell2 | Campbell2 | Tetra | Tetra |
| 21 | 36 | 25 | Campbell2 | Campbell2 | Tetra | Tetra |
| 21 | 51 | 30 | Campbell2 | Gilmore | Tetra | Hexa |
| 21 | 63 | 20 | Campbell2 | Little2 | Tetra | Hexa |
| 21 | 82 | 0 | Campbell2 | Campbell2 | Tetra | NA |
| 21 | 93 | 0 | Campbell2 | Campbell2 | Tetra | Tetra |
| 21 | 96 | 50 | Campbell2 | Campbell2 | Tetra | Tetra |
| 21 | 99 | 20 | Campbell2 | Campbell2 | Tetra | Tetra |
| 21 | 105 | 10 | Campbell2 | Gilmore | Tetra | Tetra |
| 21 | 124 | 5 | Campbell2 | Campbell2 | Tetra | Tetra |
| 22 | 7 | 0 | Little2 | Little2 | Hexa | Hexa |
| 22 | 12 | 35 | Little2 | Little2 | Hexa | Hexa |
| 22 | 17 | 26 | Little2 | Little1 | Hexa | Tetra |
| 22 | 17 | 24 | Little2 | Little1 | Hexa | Tetra |
| 22 | 22 | 2 | Little2 | Little2 | Hexa | Hexa |
| 22 | 22 | 0 | Little2 | Little2 | Hexa | Hexa |
| 22 | 32 | 19 | Little2 | Chaplin2 | Hexa | Hexa |
| 22 | 32 | 20 | Little2 | Chaplin2 | Hexa | Hexa |
| 22 | 34 | 35 | Little2 | Little2 | Hexa | Hexa |
| 22 | 46 | 10 | Little2 | Gilmore | Hexa | Tetra |
| 22 | 46 | 8 | Little2 | Gilmore | Hexa | Tetra |
| 22 | 53 | 26 | Little2 | Little1 | Hexa | Hexa |
| 22 | 63 | 0 | Little2 | Little2 | Hexa | Hexa |
| 22 | 65 | 35 | Little2 | Little2 | Hexa | Hexa |
| 22 | 67 | 30 | Little2 | Chaplin1 | Hexa | Hexa |
| 22 | 74 | 0 | Little2 | Campbell5 | Hexa | Penta |
| 22 | 90 | 0 | Little2 | Little2 | Hexa | Hexa |
| 22 | 91 | 40 | Little2 | Chaplin1 | Hexa | Hexa |
| 22 | 99 | 25 | Little2 | Campbell2 | Hexa | Tetra |
| 22 | 102 | 46 | Little2 | Little2 | Hexa | Hexa |
| 22 | 111 | 22 | Little2 | Little2 | Hexa | Hexa |
| 22 | 115 | 1 | Little2 | Little2 | Hexa | Hexa |
| 22 | 115 | 0 | Little2 | Little2 | Hexa | Hexa |
| 22 | 116 | 50 | Little2 | Little1 | Hexa | Hexa |
| 22 | 118 | 1 | Little2 | Little2 | Hexa | Hexa |
| 22 | 119 | 5 | Little2 | Campbell1 | Hexa | Tetra |
| 22 | ? | 33 | Little2 | NA | Hexa | NA |
| 25 | 9 | 20 | Campbell1 | Campbell2 | Tetra | Tetra |
| 25 | 10 | 1 | Campbell1 | Campbell1 | Tetra | Tetra |
| 25 | 25 | 0 | Campbell1 | Campbell1 | Tetra | Tetra |
| 25 | 25 | 0 | Campbell1 | Campbell1 | Tetra | Tetra |
| 25 | 25 | 0 | Campbell1 | Campbell1 | Tetra | Tetra |
| 25 | 25 | 0 | Campbell1 | Campbell1 | Tetra | Tetra |
| 25 | 25 | 0 | Campbell1 | Campbell1 | Tetra | Tetra |
| 25 | 25 | 0 | Campbell1 | Campbell1 | Tetra | Tetra |
| 25 | 25 | 0 | Campbell1 | Campbell1 | Tetra | Tetra |
| 25 | 34 | 35 | Campbell1 | Little2 | Tetra | Hexa |
| 25 | 41 | 40 | Campbell1 | Chaplin1 | Tetra | Hexa |
| 25 | 41 | 30 | Campbell1 | Chaplin1 | Tetra | Hexa |
| 25 | 51 | 7 | Campbell1 | Gilmore | Tetra | Hexa |
| 25 | 57 | 50 | Campbell1 | Campbell1 | Tetra | Tetra |
| 25 | 77 | 1 | Campbell1 | Campbell1 | Tetra | Tetra |
| 25 | 78 | 30 | Campbell1 | Campbell1 | Tetra | Tetra |
| 25 | 86 | 0 | Campbell1 | Campbell1 | Tetra | Tetra |
| 25 | 88 | 0 | Campbell1 | Campbell3 | Tetra | Tetra |
| 25 | 91 | 11 | Campbell1 | Chaplin1 | Tetra | Hexa |
| 25 | 92 | 30 | Campbell1 | Campbell3 | Tetra | Tetra |
| 25 | 92 | 35 | Campbell1 | Campbell3 | Tetra | Tetra |
| 25 | 94 | 35 | Campbell1 | Campbell4 | Tetra | Tetra |
| 25 | 96 | 0 | Campbell1 | Campbell2 | Tetra | Tetra |
| 25 | 99 | 1 | Campbell1 | Campbell2 | Tetra | Tetra |
| 25 | 119 | 40 | Campbell1 | Campbell1 | Tetra | Tetra |
| 26 | 4 | 6 | Campbell2 | Campbell2 | Tetra | Tetra |
| 26 | 9 | 0 | Campbell2 | Campbell2 | Tetra | Tetra |
| 26 | 21 | 16 | Campbell2 | Campbell2 | Tetra | Tetra |
| 26 | 26 | 0 | Campbell2 | Campbell2 | Tetra | Tetra |
| 26 | 30 | 0 | Campbell2 | Campbell2 | Tetra | Tetra |
| 26 | 36 | 4 | Campbell2 | Campbell2 | Tetra | Tetra |
| 26 | 36 | 0 | Campbell2 | Campbell2 | Tetra | Tetra |
| 26 | 93 | 0 | Campbell2 | Campbell2 | Tetra | Tetra |
| 26 | 99 | 40 | Campbell2 | Campbell2 | Tetra | Tetra |
| 27 | 27 | 0 | Campbell3 | Campbell3 | Tetra | Tetra |
| 27 | 56 | 0 | Campbell3 | Campbell3 | Tetra | Tetra |
| 27 | 58 | 8 | Campbell3 | Campbell3 | Tetra | Tetra |
| 27 | 81 | 5 | Campbell3 | Campbell3 | Tetra | Tetra |
| 27 | 87 | 15 | Campbell3 | Campbell3 | Tetra | Tetra |
| 27 | 88 | 20 | Campbell3 | Campbell3 | Tetra | Tetra |
| 27 | 92 | 0 | Campbell3 | Campbell3 | Tetra | Tetra |
| 27 | 98 | 0 | Campbell3 | Campbell3 | Tetra | Tetra |
| 27 | 108 | 25 | Campbell3 | Campbell3 | Tetra | Tetra |
| 27 | 109 | 16 | Campbell3 | Campbell3 | Tetra | Tetra |
| 27 | 121 | 0 | Campbell3 | Campbell3 | Tetra | Tetra |
| 27 | 122 | 0 | Campbell3 | Chaplin1 | Tetra | Hexa |
| 28 | 13 | 0 | Campbell4 | Campbell4 | Tetra | Tetra |
| 28 | 14 | 3 | Campbell4 | Campbell4 | Tetra | Tetra |
| 28 | 20 | 12 | Campbell4 | Campbell4 | Tetra | Penta |
| 28 | 28 | 0 | Campbell4 | Campbell4 | Tetra | Tetra |
| 28 | 31 | 0 | Campbell4 | Campbell4 | Tetra | Tetra |
| 28 | 40 | 0 | Campbell4 | Campbell4 | Tetra | Penta |
| 28 | 42 | 2 | Campbell4 | Campbell4 | Tetra | Penta |
| 28 | 42 | 0 | Campbell4 | Campbell4 | Tetra | Penta |
| 28 | 50 | 12 | Campbell4 | Campbell4 | Tetra | Tetra |
| 28 | 61 | 12 | Campbell4 | Campbell4 | Tetra | Tetra |
| 28 | 73 | 35 | Campbell4 | Campbell4 | Tetra | Tetra |
| 28 | 79 | 0 | Campbell4 | Campbell4 | Tetra | Tetra |
| 28 | 80 | 2 | Campbell4 | Campbell4 | Tetra | Penta |
| 28 | 94 | 17 | Campbell4 | Campbell4 | Tetra | Tetra |
| 28 | 95 | 35 | Campbell4 | Campbell4 | Tetra | Hexa |
| 29 | 2 | 0 | Gilmore | Gilmore | Tetra | Penta |
| 29 | 18 | 0 | Gilmore | Campbell5 | Tetra | Penta |
| 29 | 29 | 0 | Gilmore | Gilmore | Tetra | Tetra |
| 29 | 29 | 0 | Gilmore | Gilmore | Tetra | Tetra |
| 29 | 35 | 2 | Gilmore | Campbell5 | Tetra | ? |
| 29 | 35 | 2 | Gilmore | Campbell5 | Tetra | ? |
| 29 | 39 | 12 | Gilmore | Gilmore | Tetra | Tetra |
| 29 | 45 | 0 | Gilmore | Gilmore | Tetra | Tetra |
| 29 | 46 | 25 | Gilmore | Gilmore | Tetra | Tetra |
| 29 | 51 | 34 | Gilmore | Gilmore | Tetra | Hexa |
| 29 | 52 | 50 | Gilmore | Gilmore | Tetra | Hexa |
| 29 | 83 | 8 | Gilmore | Gilmore | Tetra | Hexa |
| 29 | 97 | 46 | Gilmore | Gilmore | Tetra | Tetra |
| 29 | 103 | 29 | Gilmore | Gilmore | Tetra | Hexa |
| 29 | 105 | 31 | Gilmore | Gilmore | Tetra | Tetra |
| 29 | 114 | 12 | Gilmore | Gilmore | Tetra | Tetra |
| 29 | 114 | 25 | Gilmore | Gilmore | Tetra | Tetra |
| 30 | 4 | 15 | Campbell2 | Campbell2 | Tetra | Tetra |
| 30 | 9 | 25 | Campbell2 | Campbell2 | Tetra | Tetra |
| 30 | 21 | 0 | Campbell2 | Campbell2 | Tetra | Tetra |
| 30 | 26 | 2 | Campbell2 | Campbell2 | Tetra | Tetra |
| 30 | 30 | 1 | Campbell2 | Campbell2 | Tetra | Tetra |
| 30 | 30 | 9 | Campbell2 | Campbell2 | Tetra | Tetra |
| 30 | 36 | 2 | Campbell2 | Campbell2 | Tetra | Tetra |
| 30 | 62 | 30 | Campbell2 | Campbell5 | Tetra | Tetra |
| 30 | 93 | 0 | Campbell2 | Campbell2 | Tetra | Tetra |
| 30 | 103 | 30 | Campbell2 | Gilmore | Tetra | Hexa |
| 30 | 124 | 9 | Campbell2 | Campbell2 | Tetra | Tetra |
| 31 | 13 | 0 | Campbell4 | Campbell4 | Tetra | Tetra |
| 31 | 13 | 6 | Campbell4 | Campbell4 | Tetra | Tetra |
| 31 | 14 | 10 | Campbell4 | Campbell4 | Tetra | Tetra |
| 31 | 20 | 3 | Campbell4 | Campbell4 | Tetra | Penta |
| 31 | 28 | 2 | Campbell4 | Campbell4 | Tetra | Tetra |
| 31 | 31 | 0 | Campbell4 | Campbell4 | Tetra | Tetra |
| 31 | 31 | 2 | Campbell4 | Campbell4 | Tetra | Tetra |
| 31 | 31 | 0 | Campbell4 | Campbell4 | Tetra | Tetra |
| 31 | 31 | 12 | Campbell4 | Campbell4 | Tetra | Tetra |
| 31 | 31 | 0 | Campbell4 | Campbell4 | Tetra | Tetra |
| 31 | 31 | 4 | Campbell4 | Campbell4 | Tetra | Tetra |
| 31 | 40 | 40 | Campbell4 | Campbell4 | Tetra | Penta |
| 31 | 41 | 24 | Campbell4 | Chaplin1 | Tetra | Hexa |
| 31 | 42 | 1 | Campbell4 | Campbell4 | Tetra | Penta |
| 31 | 50 | 67 | Campbell4 | Campbell4 | Tetra | Tetra |
| 31 | 61 | 3 | Campbell4 | Campbell4 | Tetra | Tetra |
| 31 | 73 | 20 | Campbell4 | Campbell4 | Tetra | Tetra |
| 31 | 79 | 0 | Campbell4 | Campbell4 | Tetra | Tetra |
| 31 | 80 | 9 | Campbell4 | Campbell4 | Tetra | Penta |
| 31 | 94 | 9 | Campbell4 | Campbell4 | Tetra | Tetra |
| 31 | 95 | 35 | Campbell4 | Campbell4 | Tetra | Hexa |
| 32 | 19 | 1 | Chaplin2 | Chaplin2 | Hexa | Hexa |
| 32 | 22 | 6 | Chaplin2 | Little2 | Hexa | Hexa |
| 32 | 32 | 3 | Chaplin2 | Chaplin2 | Hexa | Hexa |
| 32 | 32 | 1 | Chaplin2 | Chaplin2 | Hexa | Hexa |
| 32 | 41 | 20 | Chaplin2 | Chaplin1 | Hexa | Hexa |
| 32 | 49 | 0 | Chaplin2 | Chaplin2 | Hexa | Hexa |
| 32 | 66 | 7 | Chaplin2 | Chaplin2 | Hexa | Hexa |
| 32 | 66 | 8 | Chaplin2 | Chaplin2 | Hexa | Hexa |
| 32 | 67 | 16 | Chaplin2 | Chaplin1 | Hexa | Hexa |
| 32 | 91 | 35 | Chaplin2 | Chaplin1 | Hexa | Hexa |
| 32 | 104 | 33 | Chaplin2 | Chaplin2 | Hexa | Hexa |
| 32 | 113 | 2 | Chaplin2 | Chaplin2 | Hexa | Hexa |
| 32 | 113 | 1 | Chaplin2 | Chaplin2 | Hexa | Hexa |
| 32 | 117 | 13 | Chaplin2 | Chaplin2 | Hexa | Hexa |
| 33 | 7 | 0 | Little1 | Little2 | Hexa | Hexa |
| 33 | 33 | 0 | Little1 | Little1 | Hexa | Hexa |
| 33 | 33 | 6 | Little1 | Little1 | Hexa | Hexa |
| 33 | 37 | 0 | Little1 | Little1 | Hexa | Hexa |
| 33 | 37 | 0 | Little1 | Little1 | Hexa | Hexa |
| 33 | 47 | 45 | Little1 | Little1 | Hexa | Tetra |
| 33 | 53 | 0 | Little1 | Little1 | Hexa | Hexa |
| 33 | 110 | 0 | Little1 | Little1 | Hexa | Hexa |
| 33 | 116 | 65 | Little1 | Little1 | Hexa | Hexa |
| 34 | 7 | 46 | Little2 | Little2 | Hexa | Hexa |
| 34 | 12 | 30 | Little2 | Little2 | Hexa | Hexa |
| 34 | 22 | 40 | Little2 | Little2 | Hexa | Hexa |
| 34 | 25 | 44 | Little2 | Campbell1 | Hexa | Tetra |
| 34 | 34 | 0 | Little2 | Little2 | Hexa | Hexa |
| 34 | 34 | 0 | Little2 | Little2 | Hexa | Hexa |
| 34 | 47 | 31 | Little2 | Little1 | Hexa | Tetra |
| 34 | 63 | 28 | Little2 | Little2 | Hexa | Hexa |
| 34 | 65 | 0 | Little2 | Little2 | Hexa | Hexa |
| 34 | 78 | 5 | Little2 | Campbell1 | Hexa | Tetra |
| 34 | 90 | 0 | Little2 | Little2 | Hexa | Hexa |
| 34 | 102 | 35 | Little2 | Little2 | Hexa | Hexa |
| 34 | 104 | 40 | Little2 | Chaplin2 | Hexa | Hexa |
| 34 | 110 | 1 | Little2 | Little1 | Hexa | Hexa |
| 34 | 111 | 3 | Little2 | Little2 | Hexa | Hexa |
| 34 | 115 | 45 | Little2 | Little2 | Hexa | Hexa |
| 34 | 118 | 35 | Little2 | Little2 | Hexa | Hexa |
| 35 | 6 | 30 | Campbell5 | Campbell5 | ? | Penta |
| 35 | 11 | 1 | Campbell5 | Campbell5 | ? | Penta |
| 35 | 18 | 0 | Campbell5 | Campbell5 | ? | Penta |
| 35 | 18 | 0 | Campbell5 | Campbell5 | ? | Penta |
| 35 | 29 | 25 | Campbell5 | Gilmore | ? | Tetra |
| 35 | 35 | 1 | Campbell5 | Campbell5 | ? | ? |
| 35 | 35 | 0 | Campbell5 | Campbell5 | ? | ? |
| 35 | 38 | 7 | Campbell5 | Campbell5 | ? | Penta |
| 35 | 51 | 19 | Campbell5 | Gilmore | ? | Hexa |
| 35 | 62 | 35 | Campbell5 | Campbell5 | ? | Tetra |
| 35 | 62 | 25 | Campbell5 | Campbell5 | ? | Tetra |
| 35 | 64 | 13 | Campbell5 | Campbell5 | ? | Tetra |
| 35 | 71 | 0 | Campbell5 | Campbell5 | ? | Penta |
| 35 | 71 | 4 | Campbell5 | Campbell5 | ? | Penta |
| 35 | 74 | 4 | Campbell5 | Campbell5 | ? | Penta |
| 35 | 78 | 9 | Campbell5 | Campbell1 | ? | Tetra |
| 35 | 91 | 32 | Campbell5 | Chaplin1 | ? | Hexa |
| 35 | 100 | 4 | Campbell5 | Campbell5 | ? | Penta |
| 35 | 100 | 0 | Campbell5 | Campbell5 | ? | Penta |
| 35 | 101 | 3 | Campbell5 | Campbell5 | ? | ? |
| 35 | 101 | 12 | Campbell5 | Campbell5 | ? | ? |
| 35 | 106 | 15 | Campbell5 | Campbell5 | ? | Hexa |
| 36 | 4 | 0 | Campbell2 | Campbell2 | Tetra | Tetra |
| 36 | 9 | 0 | Campbell2 | Campbell2 | Tetra | Tetra |
| 36 | 21 | 0 | Campbell2 | Campbell2 | Tetra | Tetra |
| 36 | 26 | 10 | Campbell2 | Campbell2 | Tetra | Tetra |
| 36 | 30 | 5 | Campbell2 | Campbell2 | Tetra | Tetra |
| 36 | 36 | 4 | Campbell2 | Campbell2 | Tetra | Tetra |
| 36 | 36 | 0 | Campbell2 | Campbell2 | Tetra | Tetra |
| 36 | 82 | 13 | Campbell2 | Campbell2 | Tetra | NA |
| 36 | 93 | 0 | Campbell2 | Campbell2 | Tetra | Tetra |
| 36 | 96 | 25 | Campbell2 | Campbell2 | Tetra | Tetra |
| 36 | 99 | 0 | Campbell2 | Campbell2 | Tetra | Tetra |
| 36 | 99 | 20 | Campbell2 | Campbell2 | Tetra | Tetra |
| 36 | 124 | 12 | Campbell2 | Campbell2 | Tetra | Tetra |
| 37 | 17 | 13 | Little1 | Little1 | Hexa | Tetra |
| 37 | 22 | 35 | Little1 | Little2 | Hexa | Hexa |
| 37 | 33 | 15 | Little1 | Little1 | Hexa | Hexa |
| 37 | 37 | 0 | Little1 | Little1 | Hexa | Hexa |
| 37 | 37 | 0 | Little1 | Little1 | Hexa | Hexa |
| 37 | 37 | 0 | Little1 | Little1 | Hexa | Hexa |
| 37 | 47 | 40 | Little1 | Little1 | Hexa | Tetra |
| 37 | 53 | 25 | Little1 | Little1 | Hexa | Hexa |
| 37 | 72 | 40 | Little1 | Little1 | Hexa | Tetra |
| 37 | 110 | 20 | Little1 | Little1 | Hexa | Hexa |
| 37 | 116 | 35 | Little1 | Little1 | Hexa | Hexa |
| 37 | 120 | 40 | Little1 | Little1 | Hexa | Tetra |
| 38 | 6 | 6 | Campbell5 | Campbell5 | Penta | Penta |
| 38 | 11 | 0 | Campbell5 | Campbell5 | Penta | Penta |
| 38 | 18 | 1 | Campbell5 | Campbell5 | Penta | Penta |
| 38 | 18 | 5 | Campbell5 | Campbell5 | Penta | Penta |
| 38 | 35 | 8 | Campbell5 | Campbell5 | Penta | ? |
| 38 | 38 | 0 | Campbell5 | Campbell5 | Penta | Penta |
| 38 | 38 | 1 | Campbell5 | Campbell5 | Penta | Penta |
| 38 | 38 | 0 | Campbell5 | Campbell5 | Penta | Penta |
| 38 | 38 | 0 | Campbell5 | Campbell5 | Penta | Penta |
| 38 | 62 | 40 | Campbell5 | Campbell5 | Penta | Tetra |
| 38 | 64 | 0 | Campbell5 | Campbell5 | Penta | Tetra |
| 38 | 71 | 0 | Campbell5 | Campbell5 | Penta | Penta |
| 38 | 74 | 0 | Campbell5 | Campbell5 | Penta | Penta |
| 38 | 100 | 2 | Campbell5 | Campbell5 | Penta | Penta |
| 38 | 101 | 0 | Campbell5 | Campbell5 | Penta | ? |
| 38 | 106 | 20 | Campbell5 | Campbell5 | Penta | Hexa |
| 38 | 120 | 30 | Campbell5 | Little1 | Penta | Tetra |
| 39 | 2 | 0 | Gilmore | Gilmore | Tetra | Penta |
| 39 | 29 | 12 | Gilmore | Gilmore | Tetra | Tetra |
| 39 | 39 | 1 | Gilmore | Gilmore | Tetra | Tetra |
| 39 | 45 | 11 | Gilmore | Gilmore | Tetra | Tetra |
| 39 | 46 | 0 | Gilmore | Gilmore | Tetra | Tetra |
| 39 | 51 | 0 | Gilmore | Gilmore | Tetra | Hexa |
| 39 | 52 | 2 | Gilmore | Gilmore | Tetra | Hexa |
| 39 | 57 | 30 | Gilmore | Campbell1 | Tetra | Tetra |
| 39 | 83 | 16 | Gilmore | Gilmore | Tetra | Hexa |
| 39 | 97 | 0 | Gilmore | Gilmore | Tetra | Tetra |
| 39 | 103 | 30 | Gilmore | Gilmore | Tetra | Hexa |
| 39 | 105 | 0 | Gilmore | Gilmore | Tetra | Tetra |
| 39 | 114 | 0 | Gilmore | Gilmore | Tetra | Tetra |
| 40 | 13 | 38 | Campbell4 | Campbell4 | Penta | Tetra |
| 40 | 13 | 25 | Campbell4 | Campbell4 | Penta | Tetra |
| 40 | 14 | 40 | Campbell4 | Campbell4 | Penta | Tetra |
| 40 | 18 | 15 | Campbell4 | Campbell5 | Penta | Penta |
| 40 | 20 | 2 | Campbell4 | Campbell4 | Penta | Penta |
| 40 | 28 | 32 | Campbell4 | Campbell4 | Penta | Tetra |
| 40 | 31 | 40 | Campbell4 | Campbell4 | Penta | Tetra |
| 40 | 40 | 0 | Campbell4 | Campbell4 | Penta | Penta |
| 40 | 40 | 1 | Campbell4 | Campbell4 | Penta | Penta |
| 40 | 40 | 0 | Campbell4 | Campbell4 | Penta | Penta |
| 40 | 40 | 0 | Campbell4 | Campbell4 | Penta | Penta |
| 40 | 40 | 0 | Campbell4 | Campbell4 | Penta | Penta |
| 40 | 42 | 0 | Campbell4 | Campbell4 | Penta | Penta |
| 40 | 45 | 10 | Campbell4 | Gilmore | Penta | Tetra |
| 40 | 50 | 20 | Campbell4 | Campbell4 | Penta | Tetra |
| 40 | 50 | 30 | Campbell4 | Campbell4 | Penta | Tetra |
| 40 | 61 | 50 | Campbell4 | Campbell4 | Penta | Tetra |
| 40 | 67 | 15 | Campbell4 | Chaplin1 | Penta | Hexa |
| 40 | 73 | 30 | Campbell4 | Campbell4 | Penta | Tetra |
| 40 | 73 | 40 | Campbell4 | Campbell4 | Penta | Tetra |
| 40 | 79 | 35 | Campbell4 | Campbell4 | Penta | Tetra |
| 40 | 80 | 0 | Campbell4 | Campbell4 | Penta | Penta |
| 40 | 91 | 15 | Campbell4 | Chaplin1 | Penta | Hexa |
| 40 | 94 | 30 | Campbell4 | Campbell4 | Penta | Tetra |
| 40 | 94 | 25 | Campbell4 | Campbell4 | Penta | Tetra |
| 40 | 94 | 50 | Campbell4 | Campbell4 | Penta | Tetra |
| 40 | 95 | 30 | Campbell4 | Campbell4 | Penta | Hexa |
| 40 | 121 | 40 | Campbell4 | Campbell3 | Penta | Tetra |
| 41 | 1 | 6 | Chaplin1 | Chaplin1 | Hexa | Hexa |
| 41 | 5 | 30 | Chaplin1 | Chaplin1 | Hexa | Hexa |
| 41 | 11 | 1 | Chaplin1 | Campbell5 | Hexa | Penta |
| 41 | 14 | 34 | Chaplin1 | Campbell4 | Hexa | Tetra |
| 41 | 25 | 40 | Chaplin1 | Campbell1 | Hexa | Tetra |
| 41 | 25 | 12 | Chaplin1 | Campbell1 | Hexa | Tetra |
| 41 | 31 | 41 | Chaplin1 | Campbell4 | Hexa | Tetra |
| 41 | 32 | 30 | Chaplin1 | Chaplin2 | Hexa | Hexa |
| 41 | 41 | 0 | Chaplin1 | Chaplin1 | Hexa | Hexa |
| 41 | 41 | 0 | Chaplin1 | Chaplin1 | Hexa | Hexa |
| 41 | 41 | 1 | Chaplin1 | Chaplin1 | Hexa | Hexa |
| 41 | 41 | 0 | Chaplin1 | Chaplin1 | Hexa | Hexa |
| 41 | 41 | 5 | Chaplin1 | Chaplin1 | Hexa | Hexa |
| 41 | 41 | 0 | Chaplin1 | Chaplin1 | Hexa | Hexa |
| 41 | 41 | 0 | Chaplin1 | Chaplin1 | Hexa | Hexa |
| 41 | 41 | 0 | Chaplin1 | Chaplin1 | Hexa | Hexa |
| 41 | 41 | 0 | Chaplin1 | Chaplin1 | Hexa | Hexa |
| 41 | 42 | 1 | Chaplin1 | Campbell4 | Hexa | Penta |
| 41 | 42 | 16 | Chaplin1 | Campbell4 | Hexa | Penta |
| 41 | 46 | 25 | Chaplin1 | Gilmore | Hexa | Tetra |
| 41 | 50 | 30 | Chaplin1 | Campbell4 | Hexa | Tetra |
| 41 | 53 | 34 | Chaplin1 | Little1 | Hexa | Hexa |
| 41 | 54 | 35 | Chaplin1 | Chaplin1 | Hexa | Hexa |
| 41 | 56 | 35 | Chaplin1 | Campbell3 | Hexa | Tetra |
| 41 | 67 | 36 | Chaplin1 | Chaplin1 | Hexa | Hexa |
| 41 | 67 | 30 | Chaplin1 | Chaplin1 | Hexa | Hexa |
| 41 | 67 | 30 | Chaplin1 | Chaplin1 | Hexa | Hexa |
| 41 | 71 | 1 | Chaplin1 | Campbell5 | Hexa | Penta |
| 41 | 71 | 5 | Chaplin1 | Campbell5 | Hexa | Penta |
| 41 | 71 | 2 | Chaplin1 | Campbell5 | Hexa | Penta |
| 41 | 78 | 18 | Chaplin1 | Campbell1 | Hexa | Tetra |
| 41 | 89 | 15 | Chaplin1 | Chaplin1 | Hexa | Hexa |
| 41 | 91 | 52 | Chaplin1 | Chaplin1 | Hexa | Hexa |
| 41 | 91 | 30 | Chaplin1 | Chaplin1 | Hexa | Hexa |
| 41 | 94 | 15 | Chaplin1 | Campbell4 | Hexa | Tetra |
| 41 | 95 | 1 | Chaplin1 | Campbell4 | Hexa | Hexa |
| 41 | 95 | 55 | Chaplin1 | Campbell4 | Hexa | Hexa |
| 41 | 96 | 40 | Chaplin1 | Campbell2 | Hexa | Tetra |
| 41 | 101 | 6 | Chaplin1 | Campbell5 | Hexa | ? |
| 41 | 103 | 50 | Chaplin1 | Gilmore | Hexa | Hexa |
| 41 | 105 | 41 | Chaplin1 | Gilmore | Hexa | Tetra |
| 41 | 107 | 29 | Chaplin1 | Chaplin1 | Hexa | Hexa |
| 41 | 110 | 54 | Chaplin1 | Little1 | Hexa | Hexa |
| 41 | 116 | 51 | Chaplin1 | Little1 | Hexa | Hexa |
| 41 | 122 | 30 | Chaplin1 | Chaplin1 | Hexa | Hexa |
| 42 | 13 | 35 | Campbell4 | Campbell4 | Penta | Tetra |
| 42 | 14 | 23 | Campbell4 | Campbell4 | Penta | Tetra |
| 42 | 14 | 14 | Campbell4 | Campbell4 | Penta | Tetra |
| 42 | 14 | 20 | Campbell4 | Campbell4 | Penta | Tetra |
| 42 | 14 | 15 | Campbell4 | Campbell4 | Penta | Tetra |
| 42 | 20 | 0 | Campbell4 | Campbell4 | Penta | Penta |
| 42 | 28 | 3 | Campbell4 | Campbell4 | Penta | Tetra |
| 42 | 31 | 35 | Campbell4 | Campbell4 | Penta | Tetra |
| 42 | 40 | 40 | Campbell4 | Campbell4 | Penta | Penta |
| 42 | 41 | 8 | Campbell4 | Chaplin1 | Penta | Hexa |
| 42 | 41 | 25 | Campbell4 | Chaplin1 | Penta | Hexa |
| 42 | 42 | 0 | Campbell4 | Campbell4 | Penta | Penta |
| 42 | 42 | 0 | Campbell4 | Campbell4 | Penta | Penta |
| 42 | 42 | 0 | Campbell4 | Campbell4 | Penta | Penta |
| 42 | 42 | 0 | Campbell4 | Campbell4 | Penta | Penta |
| 42 | 42 | 0 | Campbell4 | Campbell4 | Penta | Penta |
| 42 | 42 | 0 | Campbell4 | Campbell4 | Penta | Penta |
| 42 | 42 | 0 | Campbell4 | Campbell4 | Penta | Penta |
| 42 | 42 | 0 | Campbell4 | Campbell4 | Penta | Penta |
| 42 | 42 | 1 | Campbell4 | Campbell4 | Penta | Penta |
| 42 | 50 | 26 | Campbell4 | Campbell4 | Penta | Tetra |
| 42 | 50 | 25 | Campbell4 | Campbell4 | Penta | Tetra |
| 42 | 61 | 40 | Campbell4 | Campbell4 | Penta | Tetra |
| 42 | 73 | 53 | Campbell4 | Campbell4 | Penta | Tetra |
| 42 | 73 | 25 | Campbell4 | Campbell4 | Penta | Tetra |
| 42 | 73 | 10 | Campbell4 | Campbell4 | Penta | Tetra |
| 42 | 79 | 44 | Campbell4 | Campbell4 | Penta | Tetra |
| 42 | 80 | 0 | Campbell4 | Campbell4 | Penta | Penta |
| 42 | 88 | 25 | Campbell4 | Campbell3 | Penta | Tetra |
| 42 | 91 | 43 | Campbell4 | Chaplin1 | Penta | Hexa |
| 42 | 91 | 35 | Campbell4 | Chaplin1 | Penta | Hexa |
| 42 | 92 | 30 | Campbell4 | Campbell3 | Penta | Tetra |
| 42 | 94 | 0 | Campbell4 | Campbell4 | Penta | Tetra |
| 42 | 94 | 30 | Campbell4 | Campbell4 | Penta | Tetra |
| 42 | 95 | 15 | Campbell4 | Campbell4 | Penta | Hexa |
| 42 | 95 | 23 | Campbell4 | Campbell4 | Penta | Hexa |
| 42 | 107 | 34 | Campbell4 | Chaplin1 | Penta | Hexa |
| 45 | 2 | 1 | Gilmore | Gilmore | Tetra | Penta |
| 45 | 2 | 0 | Gilmore | Gilmore | Tetra | Penta |
| 45 | 10 | 20 | Gilmore | Campbell1 | Tetra | Tetra |
| 45 | 14 | 25 | Gilmore | Campbell4 | Tetra | Tetra |
| 45 | 29 | 8 | Gilmore | Gilmore | Tetra | Tetra |
| 45 | 39 | 6 | Gilmore | Gilmore | Tetra | Tetra |
| 45 | 40 | 13 | Gilmore | Campbell4 | Tetra | Penta |
| 45 | 45 | 2 | Gilmore | Gilmore | Tetra | Tetra |
| 45 | 45 | 0 | Gilmore | Gilmore | Tetra | Tetra |
| 45 | 46 | 50 | Gilmore | Gilmore | Tetra | Tetra |
| 45 | 51 | 30 | Gilmore | Gilmore | Tetra | Hexa |
| 45 | 52 | 37 | Gilmore | Gilmore | Tetra | Hexa |
| 45 | 67 | 25 | Gilmore | Chaplin1 | Tetra | Hexa |
| 45 | 83 | 9 | Gilmore | Gilmore | Tetra | Hexa |
| 45 | 97 | 5 | Gilmore | Gilmore | Tetra | Tetra |
| 45 | 97 | 0 | Gilmore | Gilmore | Tetra | Tetra |
| 45 | 102 | 30 | Gilmore | Little2 | Tetra | Hexa |
| 45 | 103 | 0 | Gilmore | Gilmore | Tetra | Hexa |
| 45 | 103 | 28 | Gilmore | Gilmore | Tetra | Hexa |
| 45 | 103 | 18 | Gilmore | Gilmore | Tetra | Hexa |
| 45 | 105 | 30 | Gilmore | Gilmore | Tetra | Tetra |
| 45 | 114 | 1 | Gilmore | Gilmore | Tetra | Tetra |
| 46 | 2 | 2 | Gilmore | Gilmore | Tetra | Penta |
| 46 | 2 | 0 | Gilmore | Gilmore | Tetra | Penta |
| 46 | 4 | 15 | Gilmore | Campbell2 | Tetra | Tetra |
| 46 | 18 | 0 | Gilmore | Campbell5 | Tetra | Penta |
| 46 | 22 | 12 | Gilmore | Little2 | Tetra | Hexa |
| 46 | 29 | 30 | Gilmore | Gilmore | Tetra | Tetra |
| 46 | 39 | 0 | Gilmore | Gilmore | Tetra | Tetra |
| 46 | 45 | 40 | Gilmore | Gilmore | Tetra | Tetra |
| 46 | 46 | 0 | Gilmore | Gilmore | Tetra | Tetra |
| 46 | 46 | 0 | Gilmore | Gilmore | Tetra | Tetra |
| 46 | 49 | 42 | Gilmore | Chaplin2 | Tetra | Hexa |
| 46 | 51 | 1 | Gilmore | Gilmore | Tetra | Hexa |
| 46 | 51 | 4 | Gilmore | Gilmore | Tetra | Hexa |
| 46 | 52 | 30 | Gilmore | Gilmore | Tetra | Hexa |
| 46 | 71 | 1 | Gilmore | Campbell5 | Tetra | Penta |
| 46 | 83 | 46 | Gilmore | Gilmore | Tetra | Hexa |
| 46 | 96 | 20 | Gilmore | Campbell2 | Tetra | Tetra |
| 46 | 97 | 40 | Gilmore | Gilmore | Tetra | Tetra |
| 46 | 103 | 40 | Gilmore | Gilmore | Tetra | Hexa |
| 46 | 105 | 0 | Gilmore | Gilmore | Tetra | Tetra |
| 46 | 110 | 9 | Gilmore | Little1 | Tetra | Hexa |
| 46 | 114 | 45 | Gilmore | Gilmore | Tetra | Tetra |
| 46 | 115 | 0 | Gilmore | Little2 | Tetra | Hexa |
| 46 | 120 | 30 | Gilmore | Little1 | Tetra | Tetra |
| 47 | 17 | 2 | Little1 | Little1 | Tetra | Tetra |
| 47 | 33 | 40 | Little1 | Little1 | Tetra | Hexa |
| 47 | 34 | 34 | Little1 | Little2 | Tetra | Hexa |
| 47 | 37 | 35 | Little1 | Little1 | Tetra | Hexa |
| 47 | 47 | 0 | Little1 | Little1 | Tetra | Tetra |
| 47 | 47 | 0 | Little1 | Little1 | Tetra | Tetra |
| 47 | 53 | 35 | Little1 | Little1 | Tetra | Hexa |
| 47 | 72 | 4 | Little1 | Little1 | Tetra | Tetra |
| 47 | 110 | 50 | Little1 | Little1 | Tetra | Hexa |
| 47 | 116 | 35 | Little1 | Little1 | Tetra | Hexa |
| 47 | 120 | 1 | Little1 | Little1 | Tetra | Tetra |
| 47 | 120 | 2 | Little1 | Little1 | Tetra | Tetra |
| 47 | 123 | 35 | Little1 | Little1 | Tetra | NA |
| 48 | 27 | 0 | Campbell3 | Campbell3 | Tetra | Tetra |
| 48 | 27 | 0 | Campbell3 | Campbell3 | Tetra | Tetra |
| 48 | 48 | 0 | Campbell3 | Campbell3 | Tetra | Tetra |
| 48 | 48 | 0 | Campbell3 | Campbell3 | Tetra | Tetra |
| 48 | 56 | 0 | Campbell3 | Campbell3 | Tetra | Tetra |
| 48 | 58 | 0 | Campbell3 | Campbell3 | Tetra | Tetra |
| 48 | 87 | 20 | Campbell3 | Campbell3 | Tetra | Tetra |
| 48 | 88 | 4 | Campbell3 | Campbell3 | Tetra | Tetra |
| 48 | 92 | 10 | Campbell3 | Campbell3 | Tetra | Tetra |
| 48 | 98 | 0 | Campbell3 | Campbell3 | Tetra | Tetra |
| 48 | 108 | 0 | Campbell3 | Campbell3 | Tetra | Tetra |
| 48 | 109 | 0 | Campbell3 | Campbell3 | Tetra | Tetra |
| 48 | 121 | 0 | Campbell3 | Campbell3 | Tetra | Tetra |
| 49 | 19 | 15 | Chaplin2 | Chaplin2 | Hexa | Hexa |
| 49 | 32 | 35 | Chaplin2 | Chaplin2 | Hexa | Hexa |
| 49 | 46 | 25 | Chaplin2 | Gilmore | Hexa | Tetra |
| 49 | 49 | 0 | Chaplin2 | Chaplin2 | Hexa | Hexa |
| 49 | 49 | 0 | Chaplin2 | Chaplin2 | Hexa | Hexa |
| 49 | 49 | 0 | Chaplin2 | Chaplin2 | Hexa | Hexa |
| 49 | 49 | 0 | Chaplin2 | Chaplin2 | Hexa | Hexa |
| 49 | 49 | 0 | Chaplin2 | Chaplin2 | Hexa | Hexa |
| 49 | 49 | 0 | Chaplin2 | Chaplin2 | Hexa | Hexa |
| 49 | 66 | 25 | Chaplin2 | Chaplin2 | Hexa | Hexa |
| 49 | 66 | 1 | Chaplin2 | Chaplin2 | Hexa | Hexa |
| 49 | 69 | 0 | Chaplin2 | Chaplin2 | Hexa | Hexa |
| 49 | 104 | 30 | Chaplin2 | Chaplin2 | Hexa | Hexa |
| 49 | 113 | 30 | Chaplin2 | Chaplin2 | Hexa | Hexa |
| 49 | 117 | 30 | Chaplin2 | Chaplin2 | Hexa | Hexa |
| 50 | 13 | 0 | Campbell4 | Campbell4 | Tetra | Tetra |
| 50 | 14 | 27 | Campbell4 | Campbell4 | Tetra | Tetra |
| 50 | 14 | 25 | Campbell4 | Campbell4 | Tetra | Tetra |
| 50 | 14 | 6 | Campbell4 | Campbell4 | Tetra | Tetra |
| 50 | 14 | 11 | Campbell4 | Campbell4 | Tetra | Tetra |
| 50 | 18 | 0 | Campbell4 | Campbell5 | Tetra | Penta |
| 50 | 20 | 8 | Campbell4 | Campbell4 | Tetra | Penta |
| 50 | 28 | 30 | Campbell4 | Campbell4 | Tetra | Tetra |
| 50 | 31 | 40 | Campbell4 | Campbell4 | Tetra | Tetra |
| 50 | 40 | 7 | Campbell4 | Campbell4 | Tetra | Penta |
| 50 | 40 | 0 | Campbell4 | Campbell4 | Tetra | Penta |
| 50 | 41 | 4 | Campbell4 | Chaplin1 | Tetra | Hexa |
| 50 | 42 | 0 | Campbell4 | Campbell4 | Tetra | Penta |
| 50 | 42 | 0 | Campbell4 | Campbell4 | Tetra | Penta |
| 50 | 50 | 0 | Campbell4 | Campbell4 | Tetra | Tetra |
| 50 | 50 | 0 | Campbell4 | Campbell4 | Tetra | Tetra |
| 50 | 50 | 0 | Campbell4 | Campbell4 | Tetra | Tetra |
| 50 | 50 | 0 | Campbell4 | Campbell4 | Tetra | Tetra |
| 50 | 58 | 1 | Campbell4 | Campbell3 | Tetra | Tetra |
| 50 | 61 | 40 | Campbell4 | Campbell4 | Tetra | Tetra |
| 50 | 61 | 0 | Campbell4 | Campbell4 | Tetra | Tetra |
| 50 | 67 | 12 | Campbell4 | Chaplin1 | Tetra | Hexa |
| 50 | 73 | 0 | Campbell4 | Campbell4 | Tetra | Tetra |
| 50 | 73 | 0 | Campbell4 | Campbell4 | Tetra | Tetra |
| 50 | 78 | 2 | Campbell4 | Campbell1 | Tetra | Tetra |
| 50 | 79 | 18 | Campbell4 | Campbell4 | Tetra | Tetra |
| 50 | 80 | 1 | Campbell4 | Campbell4 | Tetra | Penta |
| 50 | 88 | 15 | Campbell4 | Campbell3 | Tetra | Tetra |
| 50 | 91 | 30 | Campbell4 | Chaplin1 | Tetra | Hexa |
| 50 | 94 | 25 | Campbell4 | Campbell4 | Tetra | Tetra |
| 50 | 94 | 2 | Campbell4 | Campbell4 | Tetra | Tetra |
| 50 | 95 | 20 | Campbell4 | Campbell4 | Tetra | Hexa |
| 50 | 98 | 30 | Campbell4 | Campbell3 | Tetra | Tetra |
| 50 | 105 | 18 | Campbell4 | Gilmore | Tetra | Tetra |
| 50 | 119 | 4 | Campbell4 | Campbell1 | Tetra | Tetra |
| 51 | 2 | 1 | Gilmore | Gilmore | Hexa | Penta |
| 51 | 6 | 6 | Gilmore | Campbell5 | Hexa | Penta |
| 51 | 11 | 5 | Gilmore | Campbell5 | Hexa | Penta |
| 51 | 25 | 20 | Gilmore | Campbell1 | Hexa | Tetra |
| 51 | 29 | 45 | Gilmore | Gilmore | Hexa | Tetra |
| 51 | 35 | 3 | Gilmore | Campbell5 | Hexa | ? |
| 51 | 39 | 30 | Gilmore | Gilmore | Hexa | Tetra |
| 51 | 45 | 25 | Gilmore | Gilmore | Hexa | Tetra |
| 51 | 46 | 1 | Gilmore | Gilmore | Hexa | Tetra |
| 51 | 46 | 2 | Gilmore | Gilmore | Hexa | Tetra |
| 51 | 51 | 9 | Gilmore | Gilmore | Hexa | Hexa |
| 51 | 51 | 0 | Gilmore | Gilmore | Hexa | Hexa |
| 51 | 52 | 35 | Gilmore | Gilmore | Hexa | Hexa |
| 51 | 69 | 20 | Gilmore | Chaplin2 | Hexa | Hexa |
| 51 | 83 | 11 | Gilmore | Gilmore | Hexa | Hexa |
| 51 | 91 | 30 | Gilmore | Chaplin1 | Hexa | Hexa |
| 51 | 97 | 40 | Gilmore | Gilmore | Hexa | Tetra |
| 51 | 103 | 35 | Gilmore | Gilmore | Hexa | Hexa |
| 51 | 103 | 40 | Gilmore | Gilmore | Hexa | Hexa |
| 51 | 105 | 0 | Gilmore | Gilmore | Hexa | Tetra |
| 51 | 114 | 40 | Gilmore | Gilmore | Hexa | Tetra |
| 51 | 114 | 30 | Gilmore | Gilmore | Hexa | Tetra |
| 51 | 116 | 30 | Gilmore | Little1 | Hexa | Hexa |
| 52 | 17 | 20 | Gilmore | Little1 | Hexa | Tetra |
| 52 | 29 | 37 | Gilmore | Gilmore | Hexa | Tetra |
| 52 | 39 | 0 | Gilmore | Gilmore | Hexa | Tetra |
| 52 | 45 | 23 | Gilmore | Gilmore | Hexa | Tetra |
| 52 | 46 | 38 | Gilmore | Gilmore | Hexa | Tetra |
| 52 | 51 | 20 | Gilmore | Gilmore | Hexa | Hexa |
| 52 | 52 | 0 | Gilmore | Gilmore | Hexa | Hexa |
| 52 | 52 | 1 | Gilmore | Gilmore | Hexa | Hexa |
| 52 | 83 | 30 | Gilmore | Gilmore | Hexa | Hexa |
| 52 | 95 | 38 | Gilmore | Campbell4 | Hexa | Hexa |
| 52 | 97 | 0 | Gilmore | Gilmore | Hexa | Tetra |
| 52 | 103 | 20 | Gilmore | Gilmore | Hexa | Hexa |
| 52 | 105 | 40 | Gilmore | Gilmore | Hexa | Tetra |
| 52 | 114 | 9 | Gilmore | Gilmore | Hexa | Tetra |
| 52 | 116 | 30 | Gilmore | Little1 | Hexa | Hexa |
| 53 | 2 | 0 | Little1 | Gilmore | Hexa | Penta |
| 53 | 17 | 35 | Little1 | Little1 | Hexa | Tetra |
| 53 | 22 | 33 | Little1 | Little2 | Hexa | Hexa |
| 53 | 33 | 0 | Little1 | Little1 | Hexa | Hexa |
| 53 | 37 | 6 | Little1 | Little1 | Hexa | Hexa |
| 53 | 41 | 30 | Little1 | Chaplin1 | Hexa | Hexa |
| 53 | 47 | 35 | Little1 | Little1 | Hexa | Tetra |
| 53 | 53 | 0 | Little1 | Little1 | Hexa | Hexa |
| 53 | 53 | 4 | Little1 | Little1 | Hexa | Hexa |
| 53 | 72 | 3 | Little1 | Little1 | Hexa | Tetra |
| 53 | 72 | 18 | Little1 | Little1 | Hexa | Tetra |
| 53 | 81 | 25 | Little1 | Campbell3 | Hexa | Tetra |
| 53 | 96 | 35 | Little1 | Campbell2 | Hexa | Tetra |
| 53 | 103 | 25 | Little1 | Gilmore | Hexa | Hexa |
| 53 | 110 | 30 | Little1 | Little1 | Hexa | Hexa |
| 53 | 116 | 30 | Little1 | Little1 | Hexa | Hexa |
| 53 | 120 | 15 | Little1 | Little1 | Hexa | Tetra |
| 53 | 123 | 13 | Little1 | Little1 | Hexa | NA |
| 53 | 123 | 3 | Little1 | Little1 | Hexa | NA |
| 54 | 1 | 6 | Chaplin1 | Chaplin1 | Hexa | Hexa |
| 54 | 5 | 22 | Chaplin1 | Chaplin1 | Hexa | Hexa |
| 54 | 41 | 5 | Chaplin1 | Chaplin1 | Hexa | Hexa |
| 54 | 54 | 0 | Chaplin1 | Chaplin1 | Hexa | Hexa |
| 54 | 54 | 0 | Chaplin1 | Chaplin1 | Hexa | Hexa |
| 54 | 54 | 0 | Chaplin1 | Chaplin1 | Hexa | Hexa |
| 54 | 54 | 0 | Chaplin1 | Chaplin1 | Hexa | Hexa |
| 54 | 67 | 23 | Chaplin1 | Chaplin1 | Hexa | Hexa |
| 54 | 89 | 6 | Chaplin1 | Chaplin1 | Hexa | Hexa |
| 54 | 91 | 25 | Chaplin1 | Chaplin1 | Hexa | Hexa |
| 54 | 107 | 20 | Chaplin1 | Chaplin1 | Hexa | Hexa |
| 54 | 122 | 6 | Chaplin1 | Chaplin1 | Hexa | Hexa |
| 56 | 27 | 0 | Campbell3 | Campbell3 | Tetra | Tetra |
| 56 | 41 | 30 | Campbell3 | Chaplin1 | Tetra | Hexa |
| 56 | 48 | 5 | Campbell3 | Campbell3 | Tetra | Tetra |
| 56 | 56 | 0 | Campbell3 | Campbell3 | Tetra | Tetra |
| 56 | 56 | 2 | Campbell3 | Campbell3 | Tetra | Tetra |
| 56 | 58 | 0 | Campbell3 | Campbell3 | Tetra | Tetra |
| 56 | 87 | 5 | Campbell3 | Campbell3 | Tetra | Tetra |
| 56 | 88 | 1 | Campbell3 | Campbell3 | Tetra | Tetra |
| 56 | 92 | 20 | Campbell3 | Campbell3 | Tetra | Tetra |
| 56 | 92 | 20 | Campbell3 | Campbell3 | Tetra | Tetra |
| 56 | 98 | 6 | Campbell3 | Campbell3 | Tetra | Tetra |
| 56 | 108 | 0 | Campbell3 | Campbell3 | Tetra | Tetra |
| 56 | 109 | 0 | Campbell3 | Campbell3 | Tetra | Tetra |
| 56 | 121 | 30 | Campbell3 | Campbell3 | Tetra | Tetra |
| 57 | 10 | 20 | Campbell1 | Campbell1 | Tetra | Tetra |
| 57 | 14 | 25 | Campbell1 | Campbell4 | Tetra | Tetra |
| 57 | 14 | 25 | Campbell1 | Campbell4 | Tetra | Tetra |
| 57 | 25 | 42 | Campbell1 | Campbell1 | Tetra | Tetra |
| 57 | 57 | 0 | Campbell1 | Campbell1 | Tetra | Tetra |
| 57 | 57 | 0 | Campbell1 | Campbell1 | Tetra | Tetra |
| 57 | 58 | 30 | Campbell1 | Campbell3 | Tetra | Tetra |
| 57 | 77 | 30 | Campbell1 | Campbell1 | Tetra | Tetra |
| 57 | 78 | 20 | Campbell1 | Campbell1 | Tetra | Tetra |
| 57 | 86 | 20 | Campbell1 | Campbell1 | Tetra | Tetra |
| 57 | 96 | 35 | Campbell1 | Campbell2 | Tetra | Tetra |
| 57 | 119 | 30 | Campbell1 | Campbell1 | Tetra | Tetra |
| 58 | 27 | 15 | Campbell3 | Campbell3 | Tetra | Tetra |
| 58 | 34 | 0 | Campbell3 | Little2 | Tetra | Hexa |
| 58 | 48 | 0 | Campbell3 | Campbell3 | Tetra | Tetra |
| 58 | 56 | 20 | Campbell3 | Campbell3 | Tetra | Tetra |
| 58 | 56 | 0 | Campbell3 | Campbell3 | Tetra | Tetra |
| 58 | 57 | 30 | Campbell3 | Campbell1 | Tetra | Tetra |
| 58 | 58 | 0 | Campbell3 | Campbell3 | Tetra | Tetra |
| 58 | 58 | 0 | Campbell3 | Campbell3 | Tetra | Tetra |
| 58 | 81 | 1 | Campbell3 | Campbell3 | Tetra | Tetra |
| 58 | 87 | 3 | Campbell3 | Campbell3 | Tetra | Tetra |
| 58 | 88 | 20 | Campbell3 | Campbell3 | Tetra | Tetra |
| 58 | 88 | 15 | Campbell3 | Campbell3 | Tetra | Tetra |
| 58 | 92 | 15 | Campbell3 | Campbell3 | Tetra | Tetra |
| 58 | 98 | 5 | Campbell3 | Campbell3 | Tetra | Tetra |
| 58 | 108 | 0 | Campbell3 | Campbell3 | Tetra | Tetra |
| 58 | 109 | 15 | Campbell3 | Campbell3 | Tetra | Tetra |
| 58 | 121 | 10 | Campbell3 | Campbell3 | Tetra | Tetra |
| 61 | 13 | 0 | Campbell4 | Campbell4 | Tetra | Tetra |
| 61 | 14 | 2 | Campbell4 | Campbell4 | Tetra | Tetra |
| 61 | 20 | 5 | Campbell4 | Campbell4 | Tetra | Penta |
| 61 | 28 | 3 | Campbell4 | Campbell4 | Tetra | Tetra |
| 61 | 31 | 0 | Campbell4 | Campbell4 | Tetra | Tetra |
| 61 | 40 | 12 | Campbell4 | Campbell4 | Tetra | Penta |
| 61 | 42 | 0 | Campbell4 | Campbell4 | Tetra | Penta |
| 61 | 50 | 35 | Campbell4 | Campbell4 | Tetra | Tetra |
| 61 | 50 | 40 | Campbell4 | Campbell4 | Tetra | Tetra |
| 61 | 61 | 0 | Campbell4 | Campbell4 | Tetra | Tetra |
| 61 | 61 | 0 | Campbell4 | Campbell4 | Tetra | Tetra |
| 61 | 73 | 20 | Campbell4 | Campbell4 | Tetra | Tetra |
| 61 | 79 | 1 | Campbell4 | Campbell4 | Tetra | Tetra |
| 61 | 80 | 2 | Campbell4 | Campbell4 | Tetra | Penta |
| 61 | 94 | 30 | Campbell4 | Campbell4 | Tetra | Tetra |
| 61 | 95 | 59 | Campbell4 | Campbell4 | Tetra | Hexa |
| 62 | 2 | 8 | Campbell5 | Gilmore | Tetra | Penta |
| 62 | 6 | 15 | Campbell5 | Campbell5 | Tetra | Penta |
| 62 | 11 | 0 | Campbell5 | Campbell5 | Tetra | Penta |
| 62 | 18 | 0 | Campbell5 | Campbell5 | Tetra | Penta |
| 62 | 35 | 40 | Campbell5 | Campbell5 | Tetra | ? |
| 62 | 35 | 40 | Campbell5 | Campbell5 | Tetra | ? |
| 62 | 38 | 20 | Campbell5 | Campbell5 | Tetra | Penta |
| 62 | 62 | 0 | Campbell5 | Campbell5 | Tetra | Tetra |
| 62 | 62 | 0 | Campbell5 | Campbell5 | Tetra | Tetra |
| 62 | 62 | 0 | Campbell5 | Campbell5 | Tetra | Tetra |
| 62 | 62 | 0 | Campbell5 | Campbell5 | Tetra | Tetra |
| 62 | 64 | 7 | Campbell5 | Campbell5 | Tetra | Tetra |
| 62 | 71 | 0 | Campbell5 | Campbell5 | Tetra | Penta |
| 62 | 71 | 1 | Campbell5 | Campbell5 | Tetra | Penta |
| 62 | 74 | 0 | Campbell5 | Campbell5 | Tetra | Penta |
| 62 | 100 | 0 | Campbell5 | Campbell5 | Tetra | Penta |
| 62 | 101 | 0 | Campbell5 | Campbell5 | Tetra | ? |
| 62 | 106 | 2 | Campbell5 | Campbell5 | Tetra | Hexa |
| 63 | 7 | 0 | Little2 | Little2 | Hexa | Hexa |
| 63 | 7 | 20 | Little2 | Little2 | Hexa | Hexa |
| 63 | 12 | 7 | Little2 | Little2 | Hexa | Hexa |
| 63 | 21 | 30 | Little2 | Campbell2 | Hexa | Tetra |
| 63 | 22 | 2 | Little2 | Little2 | Hexa | Hexa |
| 63 | 34 | 30 | Little2 | Little2 | Hexa | Hexa |
| 63 | 50 | 40 | Little2 | Campbell4 | Hexa | Tetra |
| 63 | 58 | 5 | Little2 | Campbell3 | Hexa | Tetra |
| 63 | 63 | 0 | Little2 | Little2 | Hexa | Hexa |
| 63 | 63 | 0 | Little2 | Little2 | Hexa | Hexa |
| 63 | 65 | 40 | Little2 | Little2 | Hexa | Hexa |
| 63 | 67 | 30 | Little2 | Chaplin1 | Hexa | Hexa |
| 63 | 74 | 4 | Little2 | Campbell5 | Hexa | Penta |
| 63 | 83 | 0 | Little2 | Gilmore | Hexa | Hexa |
| 63 | 90 | 41 | Little2 | Little2 | Hexa | Hexa |
| 63 | 91 | 17 | Little2 | Chaplin1 | Hexa | Hexa |
| 63 | 95 | 12 | Little2 | Campbell4 | Hexa | Hexa |
| 63 | 102 | 34 | Little2 | Little2 | Hexa | Hexa |
| 63 | 111 | 1 | Little2 | Little2 | Hexa | Hexa |
| 63 | 115 | 0 | Little2 | Little2 | Hexa | Hexa |
| 63 | 118 | 30 | Little2 | Little2 | Hexa | Hexa |
| 63 | 120 | 40 | Little2 | Little1 | Hexa | Tetra |
| 63 | 123 | 12 | Little2 | Little1 | Hexa | NA |
| 64 | 6 | 30 | Campbell5 | Campbell5 | Tetra | Penta |
| 64 | 11 | 1 | Campbell5 | Campbell5 | Tetra | Penta |
| 64 | 18 | 0 | Campbell5 | Campbell5 | Tetra | Penta |
| 64 | 35 | 12 | Campbell5 | Campbell5 | Tetra | ? |
| 64 | 38 | 0 | Campbell5 | Campbell5 | Tetra | Penta |
| 64 | 62 | 30 | Campbell5 | Campbell5 | Tetra | Tetra |
| 64 | 64 | 0 | Campbell5 | Campbell5 | Tetra | Tetra |
| 64 | 71 | 0 | Campbell5 | Campbell5 | Tetra | Penta |
| 64 | 74 | 0 | Campbell5 | Campbell5 | Tetra | Penta |
| 64 | 100 | 0 | Campbell5 | Campbell5 | Tetra | Penta |
| 64 | 101 | 2 | Campbell5 | Campbell5 | Tetra | ? |
| 64 | 106 | 6 | Campbell5 | Campbell5 | Tetra | Hexa |
| 65 | 7 | 40 | Little2 | Little2 | Hexa | Hexa |
| 65 | 12 | 25 | Little2 | Little2 | Hexa | Hexa |
| 65 | 12 | 0 | Little2 | Little2 | Hexa | Hexa |
| 65 | 22 | 25 | Little2 | Little2 | Hexa | Hexa |
| 65 | 34 | 2 | Little2 | Little2 | Hexa | Hexa |
| 65 | 63 | 25 | Little2 | Little2 | Hexa | Hexa |
| 65 | 65 | 0 | Little2 | Little2 | Hexa | Hexa |
| 65 | 65 | 0 | Little2 | Little2 | Hexa | Hexa |
| 65 | 65 | 0 | Little2 | Little2 | Hexa | Hexa |
| 65 | 65 | 0 | Little2 | Little2 | Hexa | Hexa |
| 65 | 102 | 0 | Little2 | Little2 | Hexa | Hexa |
| 65 | 110 | 15 | Little2 | Little1 | Hexa | Hexa |
| 65 | 111 | 0 | Little2 | Little2 | Hexa | Hexa |
| 65 | 115 | 20 | Little2 | Little2 | Hexa | Hexa |
| 65 | 118 | 6 | Little2 | Little2 | Hexa | Hexa |
| 66 | 2 | 7 | Chaplin2 | Gilmore | Hexa | Penta |
| 66 | 16 | 10 | Chaplin2 | Chaplin2 | Hexa | Hexa |
| 66 | 19 | 15 | Chaplin2 | Chaplin2 | Hexa | Hexa |
| 66 | 32 | 15 | Chaplin2 | Chaplin2 | Hexa | Hexa |
| 66 | 32 | 25 | Chaplin2 | Chaplin2 | Hexa | Hexa |
| 66 | 49 | 25 | Chaplin2 | Chaplin2 | Hexa | Hexa |
| 66 | 49 | 2 | Chaplin2 | Chaplin2 | Hexa | Hexa |
| 66 | 66 | 4 | Chaplin2 | Chaplin2 | Hexa | Hexa |
| 66 | 66 | 1 | Chaplin2 | Chaplin2 | Hexa | Hexa |
| 66 | 69 | 8 | Chaplin2 | Chaplin2 | Hexa | Hexa |
| 66 | 95 | 10 | Chaplin2 | Campbell4 | Hexa | Hexa |
| 66 | 104 | 40 | Chaplin2 | Chaplin2 | Hexa | Hexa |
| 66 | 104 | 0 | Chaplin2 | Chaplin2 | Hexa | Hexa |
| 66 | 113 | 12 | Chaplin2 | Chaplin2 | Hexa | Hexa |
| 66 | 117 | 0 | Chaplin2 | Chaplin2 | Hexa | Hexa |
| 66 | 118 | 7 | Chaplin2 | Little2 | Hexa | Hexa |
| 67 | 1 | 45 | Chaplin1 | Chaplin1 | Hexa | Hexa |
| 67 | 5 | 35 | Chaplin1 | Chaplin1 | Hexa | Hexa |
| 67 | 13 | 35 | Chaplin1 | Campbell4 | Hexa | Tetra |
| 67 | 22 | 7 | Chaplin1 | Little2 | Hexa | Hexa |
| 67 | 32 | 30 | Chaplin1 | Chaplin2 | Hexa | Hexa |
| 67 | 40 | 25 | Chaplin1 | Campbell4 | Hexa | Penta |
| 67 | 41 | 32 | Chaplin1 | Chaplin1 | Hexa | Hexa |
| 67 | 41 | 45 | Chaplin1 | Chaplin1 | Hexa | Hexa |
| 67 | 41 | 30 | Chaplin1 | Chaplin1 | Hexa | Hexa |
| 67 | 45 | 30 | Chaplin1 | Gilmore | Hexa | Tetra |
| 67 | 50 | 40 | Chaplin1 | Campbell4 | Hexa | Tetra |
| 67 | 54 | 41 | Chaplin1 | Chaplin1 | Hexa | Hexa |
| 67 | 63 | 20 | Chaplin1 | Little2 | Hexa | Hexa |
| 67 | 67 | 7 | Chaplin1 | Chaplin1 | Hexa | Hexa |
| 67 | 67 | 0 | Chaplin1 | Chaplin1 | Hexa | Hexa |
| 67 | 67 | 1 | Chaplin1 | Chaplin1 | Hexa | Hexa |
| 67 | 89 | 20 | Chaplin1 | Chaplin1 | Hexa | Hexa |
| 67 | 91 | 54 | Chaplin1 | Chaplin1 | Hexa | Hexa |
| 67 | 91 | 20 | Chaplin1 | Chaplin1 | Hexa | Hexa |
| 67 | 104 | 40 | Chaplin1 | Chaplin2 | Hexa | Hexa |
| 67 | 104 | 30 | Chaplin1 | Chaplin2 | Hexa | Hexa |
| 67 | 104 | 30 | Chaplin1 | Chaplin2 | Hexa | Hexa |
| 67 | 107 | 4 | Chaplin1 | Chaplin1 | Hexa | Hexa |
| 67 | 116 | 44 | Chaplin1 | Little1 | Hexa | Hexa |
| 67 | 119 | 44 | Chaplin1 | Campbell1 | Hexa | Tetra |
| 67 | 122 | 40 | Chaplin1 | Chaplin1 | Hexa | Hexa |
| 69 | 19 | 0 | Chaplin2 | Chaplin2 | Hexa | Hexa |
| 69 | 49 | 0 | Chaplin2 | Chaplin2 | Hexa | Hexa |
| 69 | 51 | 0 | Chaplin2 | Gilmore | Hexa | Hexa |
| 69 | 66 | 8 | Chaplin2 | Chaplin2 | Hexa | Hexa |
| 69 | 66 | 0 | Chaplin2 | Chaplin2 | Hexa | Hexa |
| 69 | 69 | 2 | Chaplin2 | Chaplin2 | Hexa | Hexa |
| 69 | 104 | 25 | Chaplin2 | Chaplin2 | Hexa | Hexa |
| 69 | 117 | 15 | Chaplin2 | Chaplin2 | Hexa | Hexa |
| 71 | 6 | 7 | Campbell5 | Campbell5 | Penta | Penta |
| 71 | 6 | 25 | Campbell5 | Campbell5 | Penta | Penta |
| 71 | 11 | 0 | Campbell5 | Campbell5 | Penta | Penta |
| 71 | 11 | 0 | Campbell5 | Campbell5 | Penta | Penta |
| 71 | 18 | 0 | Campbell5 | Campbell5 | Penta | Penta |
| 71 | 35 | 35 | Campbell5 | Campbell5 | Penta | ? |
| 71 | 35 | 35 | Campbell5 | Campbell5 | Penta | ? |
| 71 | 38 | 9 | Campbell5 | Campbell5 | Penta | Penta |
| 71 | 41 | 15 | Campbell5 | Chaplin1 | Penta | Hexa |
| 71 | 41 | 30 | Campbell5 | Chaplin1 | Penta | Hexa |
| 71 | 41 | 41 | Campbell5 | Chaplin1 | Penta | Hexa |
| 71 | 46 | 30 | Campbell5 | Gilmore | Penta | Tetra |
| 71 | 62 | 35 | Campbell5 | Campbell5 | Penta | Tetra |
| 71 | 62 | 20 | Campbell5 | Campbell5 | Penta | Tetra |
| 71 | 64 | 12 | Campbell5 | Campbell5 | Penta | Tetra |
| 71 | 71 | 1 | Campbell5 | Campbell5 | Penta | Penta |
| 71 | 71 | 0 | Campbell5 | Campbell5 | Penta | Penta |
| 71 | 71 | 0 | Campbell5 | Campbell5 | Penta | Penta |
| 71 | 71 | 0 | Campbell5 | Campbell5 | Penta | Penta |
| 71 | 71 | 0 | Campbell5 | Campbell5 | Penta | Penta |
| 71 | 71 | 0 | Campbell5 | Campbell5 | Penta | Penta |
| 71 | 71 | 0 | Campbell5 | Campbell5 | Penta | Penta |
| 71 | 73 | 30 | Campbell5 | Campbell4 | Penta | Tetra |
| 71 | 74 | 12 | Campbell5 | Campbell5 | Penta | Penta |
| 71 | 100 | 0 | Campbell5 | Campbell5 | Penta | Penta |
| 71 | 101 | 32 | Campbell5 | Campbell5 | Penta | ? |
| 71 | 102 | 15 | Campbell5 | Little2 | Penta | Hexa |
| 71 | 106 | 20 | Campbell5 | Campbell5 | Penta | Hexa |
| 72 | 17 | 30 | Little1 | Little1 | Tetra | Tetra |
| 72 | 17 | 20 | Little1 | Little1 | Tetra | Tetra |
| 72 | 17 | 9 | Little1 | Little1 | Tetra | Tetra |
| 72 | 37 | 40 | Little1 | Little1 | Tetra | Hexa |
| 72 | 47 | 10 | Little1 | Little1 | Tetra | Tetra |
| 72 | 72 | 0 | Little1 | Little1 | Tetra | Tetra |
| 72 | 110 | 10 | Little1 | Little1 | Tetra | Hexa |
| 72 | 116 | 30 | Little1 | Little1 | Tetra | Hexa |
| 72 | 116 | 20 | Little1 | Little1 | Tetra | Hexa |
| 72 | 120 | 40 | Little1 | Little1 | Tetra | Tetra |
| 72 | 123 | 0 | Little1 | Little1 | Tetra | NA |
| 73 | 9 | 30 | Campbell4 | Campbell2 | Tetra | Tetra |
| 73 | 13 | 20 | Campbell4 | Campbell4 | Tetra | Tetra |
| 73 | 14 | 20 | Campbell4 | Campbell4 | Tetra | Tetra |
| 73 | 20 | 15 | Campbell4 | Campbell4 | Tetra | Penta |
| 73 | 28 | 0 | Campbell4 | Campbell4 | Tetra | Tetra |
| 73 | 31 | 30 | Campbell4 | Campbell4 | Tetra | Tetra |
| 73 | 40 | 5 | Campbell4 | Campbell4 | Tetra | Penta |
| 73 | 40 | 6 | Campbell4 | Campbell4 | Tetra | Penta |
| 73 | 42 | 0 | Campbell4 | Campbell4 | Tetra | Penta |
| 73 | 42 | 0 | Campbell4 | Campbell4 | Tetra | Penta |
| 73 | 42 | 0 | Campbell4 | Campbell4 | Tetra | Penta |
| 73 | 50 | 0 | Campbell4 | Campbell4 | Tetra | Tetra |
| 73 | 50 | 0 | Campbell4 | Campbell4 | Tetra | Tetra |
| 73 | 61 | 5 | Campbell4 | Campbell4 | Tetra | Tetra |
| 73 | 71 | 0 | Campbell4 | Campbell5 | Tetra | Penta |
| 73 | 73 | 0 | Campbell4 | Campbell4 | Tetra | Tetra |
| 73 | 73 | 0 | Campbell4 | Campbell4 | Tetra | Tetra |
| 73 | 73 | 1 | Campbell4 | Campbell4 | Tetra | Tetra |
| 73 | 73 | 9 | Campbell4 | Campbell4 | Tetra | Tetra |
| 73 | 79 | 10 | Campbell4 | Campbell4 | Tetra | Tetra |
| 73 | 80 | 1 | Campbell4 | Campbell4 | Tetra | Penta |
| 73 | 91 | 12 | Campbell4 | Chaplin1 | Tetra | Hexa |
| 73 | 92 | 24 | Campbell4 | Campbell3 | Tetra | Tetra |
| 73 | 94 | 30 | Campbell4 | Campbell4 | Tetra | Tetra |
| 73 | 95 | 30 | Campbell4 | Campbell4 | Tetra | Hexa |
| 73 | 104 | 9 | Campbell4 | Chaplin2 | Tetra | Hexa |
| 73 | 105 | 2 | Campbell4 | Gilmore | Tetra | Tetra |
| 73 | 118 | 15 | Campbell4 | Little2 | Tetra | Hexa |
| 73 | 124 | 36 | Campbell4 | Campbell2 | Tetra | Tetra |
| 74 | 6 | 10 | Campbell5 | Campbell5 | Penta | Penta |
| 74 | 7 | 15 | Campbell5 | Little2 | Penta | Hexa |
| 74 | 11 | 0 | Campbell5 | Campbell5 | Penta | Penta |
| 74 | 11 | 15 | Campbell5 | Campbell5 | Penta | Penta |
| 74 | 18 | 0 | Campbell5 | Campbell5 | Penta | Penta |
| 74 | 22 | 5 | Campbell5 | Little2 | Penta | Hexa |
| 74 | 35 | 25 | Campbell5 | Campbell5 | Penta | ? |
| 74 | 38 | 4 | Campbell5 | Campbell5 | Penta | Penta |
| 74 | 62 | 30 | Campbell5 | Campbell5 | Penta | Tetra |
| 74 | 63 | 15 | Campbell5 | Little2 | Penta | Hexa |
| 74 | 64 | 0 | Campbell5 | Campbell5 | Penta | Tetra |
| 74 | 71 | 0 | Campbell5 | Campbell5 | Penta | Penta |
| 74 | 74 | 0 | Campbell5 | Campbell5 | Penta | Penta |
| 74 | 74 | 1 | Campbell5 | Campbell5 | Penta | Penta |
| 74 | 100 | 5 | Campbell5 | Campbell5 | Penta | Penta |
| 74 | 101 | 4 | Campbell5 | Campbell5 | Penta | ? |
| 74 | 106 | 20 | Campbell5 | Campbell5 | Penta | Hexa |
| 74 | 110 | 25 | Campbell5 | Little1 | Penta | Hexa |
| 77 | 57 | 20 | Campbell1 | Campbell1 | Tetra | Tetra |
| 77 | 77 | 0 | Campbell1 | Campbell1 | Tetra | Tetra |
| 77 | 77 | 0 | Campbell1 | Campbell1 | Tetra | Tetra |
| 77 | 78 | 0 | Campbell1 | Campbell1 | Tetra | Tetra |
| 77 | 86 | 0 | Campbell1 | Campbell1 | Tetra | Tetra |
| 78 | 10 | 30 | Campbell1 | Campbell1 | Tetra | Tetra |
| 78 | 25 | 20 | Campbell1 | Campbell1 | Tetra | Tetra |
| 78 | 35 | 31 | Campbell1 | Campbell5 | Tetra | ? |
| 78 | 41 | 2 | Campbell1 | Chaplin1 | Tetra | Hexa |
| 78 | 50 | 43 | Campbell1 | Campbell4 | Tetra | Tetra |
| 78 | 57 | 15 | Campbell1 | Campbell1 | Tetra | Tetra |
| 78 | 77 | 30 | Campbell1 | Campbell1 | Tetra | Tetra |
| 78 | 78 | 0 | Campbell1 | Campbell1 | Tetra | Tetra |
| 78 | 78 | 5 | Campbell1 | Campbell1 | Tetra | Tetra |
| 78 | 86 | 12 | Campbell1 | Campbell1 | Tetra | Tetra |
| 78 | 86 | 15 | Campbell1 | Campbell1 | Tetra | Tetra |
| 78 | 95 | 25 | Campbell1 | Campbell4 | Tetra | Hexa |
| 78 | 96 | 25 | Campbell1 | Campbell2 | Tetra | Tetra |
| 78 | 102 | 25 | Campbell1 | Little2 | Tetra | Hexa |
| 78 | 105 | 15 | Campbell1 | Gilmore | Tetra | Tetra |
| 78 | 107 | 38 | Campbell1 | Chaplin1 | Tetra | Hexa |
| 78 | 119 | 20 | Campbell1 | Campbell1 | Tetra | Tetra |
| 79 | 13 | 0 | Campbell4 | Campbell4 | Tetra | Tetra |
| 79 | 14 | 0 | Campbell4 | Campbell4 | Tetra | Tetra |
| 79 | 20 | 8 | Campbell4 | Campbell4 | Tetra | Penta |
| 79 | 28 | 0 | Campbell4 | Campbell4 | Tetra | Tetra |
| 79 | 31 | 1 | Campbell4 | Campbell4 | Tetra | Tetra |
| 79 | 40 | 20 | Campbell4 | Campbell4 | Tetra | Penta |
| 79 | 42 | 1 | Campbell4 | Campbell4 | Tetra | Penta |
| 79 | 50 | 50 | Campbell4 | Campbell4 | Tetra | Tetra |
| 79 | 61 | 0 | Campbell4 | Campbell4 | Tetra | Tetra |
| 79 | 73 | 30 | Campbell4 | Campbell4 | Tetra | Tetra |
| 79 | 79 | 3 | Campbell4 | Campbell4 | Tetra | Tetra |
| 79 | 79 | 0 | Campbell4 | Campbell4 | Tetra | Tetra |
| 79 | 80 | 2 | Campbell4 | Campbell4 | Tetra | Penta |
| 79 | 94 | 20 | Campbell4 | Campbell4 | Tetra | Tetra |
| 79 | 94 | 10 | Campbell4 | Campbell4 | Tetra | Tetra |
| 79 | 95 | 15 | Campbell4 | Campbell4 | Tetra | Hexa |
| 80 | 4 | 20 | Campbell4 | Campbell2 | Penta | Tetra |
| 80 | 13 | 30 | Campbell4 | Campbell4 | Penta | Tetra |
| 80 | 14 | 0 | Campbell4 | Campbell4 | Penta | Tetra |
| 80 | 20 | 1 | Campbell4 | Campbell4 | Penta | Penta |
| 80 | 28 | 15 | Campbell4 | Campbell4 | Penta | Tetra |
| 80 | 31 | 30 | Campbell4 | Campbell4 | Penta | Tetra |
| 80 | 40 | 0 | Campbell4 | Campbell4 | Penta | Penta |
| 80 | 42 | 0 | Campbell4 | Campbell4 | Penta | Penta |
| 80 | 50 | 35 | Campbell4 | Campbell4 | Penta | Tetra |
| 80 | 61 | 0 | Campbell4 | Campbell4 | Penta | Tetra |
| 80 | 73 | 35 | Campbell4 | Campbell4 | Penta | Tetra |
| 80 | 79 | 7 | Campbell4 | Campbell4 | Penta | Tetra |
| 80 | 80 | 0 | Campbell4 | Campbell4 | Penta | Penta |
| 80 | 80 | 2 | Campbell4 | Campbell4 | Penta | Penta |
| 80 | 94 | 0 | Campbell4 | Campbell4 | Penta | Tetra |
| 80 | 95 | 30 | Campbell4 | Campbell4 | Penta | Hexa |
| 81 | 27 | 5 | Campbell3 | Campbell3 | Tetra | Tetra |
| 81 | 48 | 0 | Campbell3 | Campbell3 | Tetra | Tetra |
| 81 | 53 | 25 | Campbell3 | Little1 | Tetra | Hexa |
| 81 | 56 | 0 | Campbell3 | Campbell3 | Tetra | Tetra |
| 81 | 58 | 0 | Campbell3 | Campbell3 | Tetra | Tetra |
| 81 | 81 | 0 | Campbell3 | Campbell3 | Tetra | Tetra |
| 81 | 81 | 0 | Campbell3 | Campbell3 | Tetra | Tetra |
| 81 | 87 | 20 | Campbell3 | Campbell3 | Tetra | Tetra |
| 81 | 88 | 0 | Campbell3 | Campbell3 | Tetra | Tetra |
| 81 | 92 | 10 | Campbell3 | Campbell3 | Tetra | Tetra |
| 81 | 98 | 10 | Campbell3 | Campbell3 | Tetra | Tetra |
| 81 | 108 | 0 | Campbell3 | Campbell3 | Tetra | Tetra |
| 81 | 109 | 2 | Campbell3 | Campbell3 | Tetra | Tetra |
| 81 | 120 | 25 | Campbell3 | Little1 | Tetra | Tetra |
| 81 | 121 | 2 | Campbell3 | Campbell3 | Tetra | Tetra |
| 82 | 9 | 0 | Campbell2 | Campbell2 | NA | Tetra |
| 82 | 17 | 3 | Campbell2 | Little1 | NA | Tetra |
| 82 | 21 | 0 | Campbell2 | Campbell2 | NA | Tetra |
| 82 | 26 | 0 | Campbell2 | Campbell2 | NA | Tetra |
| 82 | 30 | 2 | Campbell2 | Campbell2 | NA | Tetra |
| 82 | 82 | 0 | Campbell2 | Campbell2 | NA | NA |
| 82 | 93 | 0 | Campbell2 | Campbell2 | NA | Tetra |
| 82 | 96 | 1 | Campbell2 | Campbell2 | NA | Tetra |
| 82 | 99 | 12 | Campbell2 | Campbell2 | NA | Tetra |
| 82 | 118 | 5 | Campbell2 | Little2 | NA | Hexa |
| 82 | 124 | 12 | Campbell2 | Campbell2 | NA | Tetra |
| 83 | 2 | 2 | Gilmore | Gilmore | Hexa | Penta |
| 83 | 29 | 16 | Gilmore | Gilmore | Hexa | Tetra |
| 83 | 39 | 24 | Gilmore | Gilmore | Hexa | Tetra |
| 83 | 45 | 1 | Gilmore | Gilmore | Hexa | Tetra |
| 83 | 46 | 25 | Gilmore | Gilmore | Hexa | Tetra |
| 83 | 51 | 0 | Gilmore | Gilmore | Hexa | Hexa |
| 83 | 52 | 24 | Gilmore | Gilmore | Hexa | Hexa |
| 83 | 83 | 1 | Gilmore | Gilmore | Hexa | Hexa |
| 83 | 83 | 0 | Gilmore | Gilmore | Hexa | Hexa |
| 83 | 97 | 20 | Gilmore | Gilmore | Hexa | Tetra |
| 83 | 103 | 40 | Gilmore | Gilmore | Hexa | Hexa |
| 83 | 105 | 20 | Gilmore | Gilmore | Hexa | Tetra |
| 83 | 114 | 30 | Gilmore | Gilmore | Hexa | Tetra |
| 86 | 10 | 10 | Campbell1 | Campbell1 | Tetra | Tetra |
| 86 | 25 | 20 | Campbell1 | Campbell1 | Tetra | Tetra |
| 86 | 57 | 10 | Campbell1 | Campbell1 | Tetra | Tetra |
| 86 | 78 | 4 | Campbell1 | Campbell1 | Tetra | Tetra |
| 86 | 78 | 13 | Campbell1 | Campbell1 | Tetra | Tetra |
| 86 | 86 | 1 | Campbell1 | Campbell1 | Tetra | Tetra |
| 86 | 86 | 0 | Campbell1 | Campbell1 | Tetra | Tetra |
| 86 | 119 | 26 | Campbell1 | Campbell1 | Tetra | Tetra |
| 87 | 7 | 4 | Campbell3 | Little2 | Tetra | Hexa |
| 87 | 27 | 25 | Campbell3 | Campbell3 | Tetra | Tetra |
| 87 | 48 | 13 | Campbell3 | Campbell3 | Tetra | Tetra |
| 87 | 56 | 7 | Campbell3 | Campbell3 | Tetra | Tetra |
| 87 | 58 | 25 | Campbell3 | Campbell3 | Tetra | Tetra |
| 87 | 81 | 20 | Campbell3 | Campbell3 | Tetra | Tetra |
| 87 | 87 | 0 | Campbell3 | Campbell3 | Tetra | Tetra |
| 87 | 87 | 0 | Campbell3 | Campbell3 | Tetra | Tetra |
| 87 | 88 | 1 | Campbell3 | Campbell3 | Tetra | Tetra |
| 87 | 92 | 30 | Campbell3 | Campbell3 | Tetra | Tetra |
| 87 | 92 | 7 | Campbell3 | Campbell3 | Tetra | Tetra |
| 87 | 98 | 22 | Campbell3 | Campbell3 | Tetra | Tetra |
| 87 | 98 | 20 | Campbell3 | Campbell3 | Tetra | Tetra |
| 87 | 106 | 0 | Campbell3 | Campbell5 | Tetra | Hexa |
| 87 | 108 | 40 | Campbell3 | Campbell3 | Tetra | Tetra |
| 87 | 109 | 0 | Campbell3 | Campbell3 | Tetra | Tetra |
| 87 | 121 | 30 | Campbell3 | Campbell3 | Tetra | Tetra |
| 88 | 7 | 0 | Campbell3 | Little2 | Tetra | Hexa |
| 88 | 10 | 0 | Campbell3 | Campbell1 | Tetra | Tetra |
| 88 | 14 | 10 | Campbell3 | Campbell4 | Tetra | Tetra |
| 88 | 25 | 15 | Campbell3 | Campbell1 | Tetra | Tetra |
| 88 | 27 | 30 | Campbell3 | Campbell3 | Tetra | Tetra |
| 88 | 42 | 20 | Campbell3 | Campbell4 | Tetra | Penta |
| 88 | 48 | 20 | Campbell3 | Campbell3 | Tetra | Tetra |
| 88 | 50 | 15 | Campbell3 | Campbell4 | Tetra | Tetra |
| 88 | 56 | 6 | Campbell3 | Campbell3 | Tetra | Tetra |
| 88 | 58 | 10 | Campbell3 | Campbell3 | Tetra | Tetra |
| 88 | 81 | 25 | Campbell3 | Campbell3 | Tetra | Tetra |
| 88 | 87 | 12 | Campbell3 | Campbell3 | Tetra | Tetra |
| 88 | 88 | 0 | Campbell3 | Campbell3 | Tetra | Tetra |
| 88 | 88 | 0 | Campbell3 | Campbell3 | Tetra | Tetra |
| 88 | 88 | 2 | Campbell3 | Campbell3 | Tetra | Tetra |
| 88 | 88 | 0 | Campbell3 | Campbell3 | Tetra | Tetra |
| 88 | 92 | 20 | Campbell3 | Campbell3 | Tetra | Tetra |
| 88 | 92 | 15 | Campbell3 | Campbell3 | Tetra | Tetra |
| 88 | 95 | 35 | Campbell3 | Campbell4 | Tetra | Hexa |
| 88 | 98 | 45 | Campbell3 | Campbell3 | Tetra | Tetra |
| 88 | 105 | 0 | Campbell3 | Gilmore | Tetra | Tetra |
| 88 | 108 | 24 | Campbell3 | Campbell3 | Tetra | Tetra |
| 88 | 109 | 30 | Campbell3 | Campbell3 | Tetra | Tetra |
| 88 | 121 | 5 | Campbell3 | Campbell3 | Tetra | Tetra |
| 89 | 1 | 2 | Chaplin1 | Chaplin1 | Hexa | Hexa |
| 89 | 5 | 3 | Chaplin1 | Chaplin1 | Hexa | Hexa |
| 89 | 41 | 20 | Chaplin1 | Chaplin1 | Hexa | Hexa |
| 89 | 54 | 14 | Chaplin1 | Chaplin1 | Hexa | Hexa |
| 89 | 67 | 6 | Chaplin1 | Chaplin1 | Hexa | Hexa |
| 89 | 89 | 0 | Chaplin1 | Chaplin1 | Hexa | Hexa |
| 89 | 89 | 0 | Chaplin1 | Chaplin1 | Hexa | Hexa |
| 89 | 91 | 12 | Chaplin1 | Chaplin1 | Hexa | Hexa |
| 89 | 107 | 0 | Chaplin1 | Chaplin1 | Hexa | Hexa |
| 89 | 122 | 25 | Chaplin1 | Chaplin1 | Hexa | Hexa |
| 90 | 12 | 13 | Little2 | Little2 | Hexa | Hexa |
| 90 | 22 | 35 | Little2 | Little2 | Hexa | Hexa |
| 90 | 34 | 8 | Little2 | Little2 | Hexa | Hexa |
| 90 | 63 | 44 | Little2 | Little2 | Hexa | Hexa |
| 90 | 65 | 0 | Little2 | Little2 | Hexa | Hexa |
| 90 | 90 | 0 | Little2 | Little2 | Hexa | Hexa |
| 90 | 102 | 20 | Little2 | Little2 | Hexa | Hexa |
| 90 | 111 | 0 | Little2 | Little2 | Hexa | Hexa |
| 90 | 115 | 30 | Little2 | Little2 | Hexa | Hexa |
| 90 | 118 | 5 | Little2 | Little2 | Hexa | Hexa |
| 90 | 118 | 5 | Little2 | Little2 | Hexa | Hexa |
| 91 | 1 | 30 | Chaplin1 | Chaplin1 | Hexa | Hexa |
| 91 | 2 | 3 | Chaplin1 | Gilmore | Hexa | Penta |
| 91 | 5 | 14 | Chaplin1 | Chaplin1 | Hexa | Hexa |
| 91 | 22 | 31 | Chaplin1 | Little2 | Hexa | Hexa |
| 91 | 25 | 20 | Chaplin1 | Campbell1 | Hexa | Tetra |
| 91 | 32 | 40 | Chaplin1 | Chaplin2 | Hexa | Hexa |
| 91 | 35 | 0 | Chaplin1 | Campbell5 | Hexa | ? |
| 91 | 40 | 10 | Chaplin1 | Campbell4 | Hexa | Penta |
| 91 | 41 | 37 | Chaplin1 | Chaplin1 | Hexa | Hexa |
| 91 | 41 | 8 | Chaplin1 | Chaplin1 | Hexa | Hexa |
| 91 | 42 | 2 | Chaplin1 | Campbell4 | Hexa | Penta |
| 91 | 50 | 35 | Chaplin1 | Campbell4 | Hexa | Tetra |
| 91 | 51 | 40 | Chaplin1 | Gilmore | Hexa | Hexa |
| 91 | 54 | 25 | Chaplin1 | Chaplin1 | Hexa | Hexa |
| 91 | 63 | 20 | Chaplin1 | Little2 | Hexa | Hexa |
| 91 | 67 | 44 | Chaplin1 | Chaplin1 | Hexa | Hexa |
| 91 | 67 | 20 | Chaplin1 | Chaplin1 | Hexa | Hexa |
| 91 | 73 | 25 | Chaplin1 | Campbell4 | Hexa | Tetra |
| 91 | 89 | 11 | Chaplin1 | Chaplin1 | Hexa | Hexa |
| 91 | 91 | 0 | Chaplin1 | Chaplin1 | Hexa | Hexa |
| 91 | 91 | 12 | Chaplin1 | Chaplin1 | Hexa | Hexa |
| 91 | 91 | 5 | Chaplin1 | Chaplin1 | Hexa | Hexa |
| 91 | 91 | 0 | Chaplin1 | Chaplin1 | Hexa | Hexa |
| 91 | 91 | 1 | Chaplin1 | Chaplin1 | Hexa | Hexa |
| 91 | 91 | 0 | Chaplin1 | Chaplin1 | Hexa | Hexa |
| 91 | 91 | 0 | Chaplin1 | Chaplin1 | Hexa | Hexa |
| 91 | 102 | 15 | Chaplin1 | Little2 | Hexa | Hexa |
| 91 | 104 | 20 | Chaplin1 | Chaplin2 | Hexa | Hexa |
| 91 | 107 | 42 | Chaplin1 | Chaplin1 | Hexa | Hexa |
| 91 | 107 | 10 | Chaplin1 | Chaplin1 | Hexa | Hexa |
| 91 | 120 | 38 | Chaplin1 | Little1 | Hexa | Tetra |
| 91 | 122 | 35 | Chaplin1 | Chaplin1 | Hexa | Hexa |
| 92 | 25 | 13 | Campbell3 | Campbell1 | Tetra | Tetra |
| 92 | 27 | 4 | Campbell3 | Campbell3 | Tetra | Tetra |
| 92 | 42 | 0 | Campbell3 | Campbell4 | Tetra | Penta |
| 92 | 48 | 18 | Campbell3 | Campbell3 | Tetra | Tetra |
| 92 | 56 | 35 | Campbell3 | Campbell3 | Tetra | Tetra |
| 92 | 58 | 18 | Campbell3 | Campbell3 | Tetra | Tetra |
| 92 | 73 | 35 | Campbell3 | Campbell4 | Tetra | Tetra |
| 92 | 81 | 22 | Campbell3 | Campbell3 | Tetra | Tetra |
| 92 | 87 | 30 | Campbell3 | Campbell3 | Tetra | Tetra |
| 92 | 87 | 20 | Campbell3 | Campbell3 | Tetra | Tetra |
| 92 | 88 | 40 | Campbell3 | Campbell3 | Tetra | Tetra |
| 92 | 88 | 25 | Campbell3 | Campbell3 | Tetra | Tetra |
| 92 | 91 | 30 | Campbell3 | Chaplin1 | Tetra | Hexa |
| 92 | 92 | 0 | Campbell3 | Campbell3 | Tetra | Tetra |
| 92 | 92 | 0 | Campbell3 | Campbell3 | Tetra | Tetra |
| 92 | 98 | 15 | Campbell3 | Campbell3 | Tetra | Tetra |
| 92 | 108 | 25 | Campbell3 | Campbell3 | Tetra | Tetra |
| 92 | 109 | 30 | Campbell3 | Campbell3 | Tetra | Tetra |
| 92 | 121 | 13 | Campbell3 | Campbell3 | Tetra | Tetra |
| 93 | 4 | 20 | Campbell2 | Campbell2 | Tetra | Tetra |
| 93 | 9 | 25 | Campbell2 | Campbell2 | Tetra | Tetra |
| 93 | 26 | 6 | Campbell2 | Campbell2 | Tetra | Tetra |
| 93 | 30 | 0 | Campbell2 | Campbell2 | Tetra | Tetra |
| 93 | 36 | 4 | Campbell2 | Campbell2 | Tetra | Tetra |
| 93 | 82 | 25 | Campbell2 | Campbell2 | Tetra | NA |
| 93 | 93 | 0 | Campbell2 | Campbell2 | Tetra | Tetra |
| 93 | 96 | 0 | Campbell2 | Campbell2 | Tetra | Tetra |
| 93 | 96 | 0 | Campbell2 | Campbell2 | Tetra | Tetra |
| 93 | 99 | 0 | Campbell2 | Campbell2 | Tetra | Tetra |
| 94 | 13 | 0 | Campbell4 | Campbell4 | Tetra | Tetra |
| 94 | 14 | 35 | Campbell4 | Campbell4 | Tetra | Tetra |
| 94 | 20 | 0 | Campbell4 | Campbell4 | Tetra | Penta |
| 94 | 25 | 20 | Campbell4 | Campbell1 | Tetra | Tetra |
| 94 | 25 | 15 | Campbell4 | Campbell1 | Tetra | Tetra |
| 94 | 28 | 4 | Campbell4 | Campbell4 | Tetra | Tetra |
| 94 | 31 | 25 | Campbell4 | Campbell4 | Tetra | Tetra |
| 94 | 40 | 20 | Campbell4 | Campbell4 | Tetra | Penta |
| 94 | 40 | 25 | Campbell4 | Campbell4 | Tetra | Penta |
| 94 | 40 | 11 | Campbell4 | Campbell4 | Tetra | Penta |
| 94 | 41 | 40 | Campbell4 | Chaplin1 | Tetra | Hexa |
| 94 | 42 | 1 | Campbell4 | Campbell4 | Tetra | Penta |
| 94 | 50 | 30 | Campbell4 | Campbell4 | Tetra | Tetra |
| 94 | 50 | 12 | Campbell4 | Campbell4 | Tetra | Tetra |
| 94 | 61 | 6 | Campbell4 | Campbell4 | Tetra | Tetra |
| 94 | 73 | 30 | Campbell4 | Campbell4 | Tetra | Tetra |
| 94 | 79 | 25 | Campbell4 | Campbell4 | Tetra | Tetra |
| 94 | 79 | 10 | Campbell4 | Campbell4 | Tetra | Tetra |
| 94 | 80 | 0 | Campbell4 | Campbell4 | Tetra | Penta |
| 94 | 94 | 2 | Campbell4 | Campbell4 | Tetra | Tetra |
| 94 | 94 | 0 | Campbell4 | Campbell4 | Tetra | Tetra |
| 94 | 94 | 6 | Campbell4 | Campbell4 | Tetra | Tetra |
| 94 | 94 | 0 | Campbell4 | Campbell4 | Tetra | Tetra |
| 94 | 94 | 0 | Campbell4 | Campbell4 | Tetra | Tetra |
| 94 | 94 | 0 | Campbell4 | Campbell4 | Tetra | Tetra |
| 94 | 94 | 10 | Campbell4 | Campbell4 | Tetra | Tetra |
| 94 | 95 | 40 | Campbell4 | Campbell4 | Tetra | Hexa |
| 95 | 13 | 30 | Campbell4 | Campbell4 | Hexa | Tetra |
| 95 | 13 | 25 | Campbell4 | Campbell4 | Hexa | Tetra |
| 95 | 14 | 15 | Campbell4 | Campbell4 | Hexa | Tetra |
| 95 | 19 | 30 | Campbell4 | Chaplin2 | Hexa | Hexa |
| 95 | 20 | 0 | Campbell4 | Campbell4 | Hexa | Penta |
| 95 | 28 | 20 | Campbell4 | Campbell4 | Hexa | Tetra |
| 95 | 31 | 20 | Campbell4 | Campbell4 | Hexa | Tetra |
| 95 | 40 | 10 | Campbell4 | Campbell4 | Hexa | Penta |
| 95 | 41 | 0 | Campbell4 | Chaplin1 | Hexa | Hexa |
| 95 | 41 | 26 | Campbell4 | Chaplin1 | Hexa | Hexa |
| 95 | 42 | 0 | Campbell4 | Campbell4 | Hexa | Penta |
| 95 | 50 | 25 | Campbell4 | Campbell4 | Hexa | Tetra |
| 95 | 61 | 28 | Campbell4 | Campbell4 | Hexa | Tetra |
| 95 | 63 | 12 | Campbell4 | Little2 | Hexa | Hexa |
| 95 | 66 | 2 | Campbell4 | Chaplin2 | Hexa | Hexa |
| 95 | 73 | 35 | Campbell4 | Campbell4 | Hexa | Tetra |
| 95 | 78 | 30 | Campbell4 | Campbell1 | Hexa | Tetra |
| 95 | 79 | 20 | Campbell4 | Campbell4 | Hexa | Tetra |
| 95 | 80 | 1 | Campbell4 | Campbell4 | Hexa | Penta |
| 95 | 88 | 15 | Campbell4 | Campbell3 | Hexa | Tetra |
| 95 | 94 | 23 | Campbell4 | Campbell4 | Hexa | Tetra |
| 95 | 95 | 0 | Campbell4 | Campbell4 | Hexa | Hexa |
| 95 | 95 | 1 | Campbell4 | Campbell4 | Hexa | Hexa |
| 95 | 109 | 2 | Campbell4 | Campbell3 | Hexa | Tetra |
| 95 | 116 | 9 | Campbell4 | Little1 | Hexa | Hexa |
| 96 | 4 | 20 | Campbell2 | Campbell2 | Tetra | Tetra |
| 96 | 4 | 35 | Campbell2 | Campbell2 | Tetra | Tetra |
| 96 | 9 | 8 | Campbell2 | Campbell2 | Tetra | Tetra |
| 96 | 11 | 7 | Campbell2 | Campbell5 | Tetra | Penta |
| 96 | 21 | 20 | Campbell2 | Campbell2 | Tetra | Tetra |
| 96 | 25 | 10 | Campbell2 | Campbell1 | Tetra | Tetra |
| 96 | 26 | 3 | Campbell2 | Campbell2 | Tetra | Tetra |
| 96 | 30 | 25 | Campbell2 | Campbell2 | Tetra | Tetra |
| 96 | 40 | 7 | Campbell2 | Campbell4 | Tetra | Penta |
| 96 | 41 | 42 | Campbell2 | Chaplin1 | Tetra | Hexa |
| 96 | 46 | 20 | Campbell2 | Gilmore | Tetra | Tetra |
| 96 | 53 | 4 | Campbell2 | Little1 | Tetra | Hexa |
| 96 | 57 | 30 | Campbell2 | Campbell1 | Tetra | Tetra |
| 96 | 78 | 11 | Campbell2 | Campbell1 | Tetra | Tetra |
| 96 | 82 | 14 | Campbell2 | Campbell2 | Tetra | NA |
| 96 | 93 | 0 | Campbell2 | Campbell2 | Tetra | Tetra |
| 96 | 96 | 0 | Campbell2 | Campbell2 | Tetra | Tetra |
| 96 | 96 | 0 | Campbell2 | Campbell2 | Tetra | Tetra |
| 96 | 99 | 8 | Campbell2 | Campbell2 | Tetra | Tetra |
| 96 | 107 | 49 | Campbell2 | Chaplin1 | Tetra | Hexa |
| 96 | 124 | 25 | Campbell2 | Campbell2 | Tetra | Tetra |
| 97 | 2 | 20 | Gilmore | Gilmore | Tetra | Penta |
| 97 | 29 | 15 | Gilmore | Gilmore | Tetra | Tetra |
| 97 | 39 | 3 | Gilmore | Gilmore | Tetra | Tetra |
| 97 | 45 | 10 | Gilmore | Gilmore | Tetra | Tetra |
| 97 | 45 | 3 | Gilmore | Gilmore | Tetra | Tetra |
| 97 | 46 | 40 | Gilmore | Gilmore | Tetra | Tetra |
| 97 | 51 | 40 | Gilmore | Gilmore | Tetra | Hexa |
| 97 | 52 | 12 | Gilmore | Gilmore | Tetra | Hexa |
| 97 | 83 | 25 | Gilmore | Gilmore | Tetra | Hexa |
| 97 | 97 | 0 | Gilmore | Gilmore | Tetra | Tetra |
| 97 | 103 | 16 | Gilmore | Gilmore | Tetra | Hexa |
| 97 | 105 | 13 | Gilmore | Gilmore | Tetra | Tetra |
| 97 | 114 | 6 | Gilmore | Gilmore | Tetra | Tetra |
| 98 | 27 | 4 | Campbell3 | Campbell3 | Tetra | Tetra |
| 98 | 48 | 0 | Campbell3 | Campbell3 | Tetra | Tetra |
| 98 | 50 | 30 | Campbell3 | Campbell4 | Tetra | Tetra |
| 98 | 56 | 0 | Campbell3 | Campbell3 | Tetra | Tetra |
| 98 | 58 | 0 | Campbell3 | Campbell3 | Tetra | Tetra |
| 98 | 87 | 10 | Campbell3 | Campbell3 | Tetra | Tetra |
| 98 | 88 | 50 | Campbell3 | Campbell3 | Tetra | Tetra |
| 98 | 92 | 6 | Campbell3 | Campbell3 | Tetra | Tetra |
| 98 | 98 | 0 | Campbell3 | Campbell3 | Tetra | Tetra |
| 98 | 98 | 0 | Campbell3 | Campbell3 | Tetra | Tetra |
| 98 | 108 | 4 | Campbell3 | Campbell3 | Tetra | Tetra |
| 98 | 109 | 20 | Campbell3 | Campbell3 | Tetra | Tetra |
| 98 | 109 | 40 | Campbell3 | Campbell3 | Tetra | Tetra |
| 98 | 118 | 25 | Campbell3 | Little2 | Tetra | Hexa |
| 98 | 121 | 20 | Campbell3 | Campbell3 | Tetra | Tetra |
| 99 | 4 | 35 | Campbell2 | Campbell2 | Tetra | Tetra |
| 99 | 9 | 0 | Campbell2 | Campbell2 | Tetra | Tetra |
| 99 | 21 | 25 | Campbell2 | Campbell2 | Tetra | Tetra |
| 99 | 22 | 13 | Campbell2 | Little2 | Tetra | Hexa |
| 99 | 25 | 0 | Campbell2 | Campbell1 | Tetra | Tetra |
| 99 | 26 | 35 | Campbell2 | Campbell2 | Tetra | Tetra |
| 99 | 30 | 1 | Campbell2 | Campbell2 | Tetra | Tetra |
| 99 | 36 | 3 | Campbell2 | Campbell2 | Tetra | Tetra |
| 99 | 36 | 11 | Campbell2 | Campbell2 | Tetra | Tetra |
| 99 | 82 | 6 | Campbell2 | Campbell2 | Tetra | NA |
| 99 | 93 | 0 | Campbell2 | Campbell2 | Tetra | Tetra |
| 99 | 95 | 0 | Campbell2 | Campbell4 | Tetra | Hexa |
| 99 | 96 | 0 | Campbell2 | Campbell2 | Tetra | Tetra |
| 99 | 99 | 0 | Campbell2 | Campbell2 | Tetra | Tetra |
| 99 | 99 | 0 | Campbell2 | Campbell2 | Tetra | Tetra |
| 99 | 110 | 0 | Campbell2 | Little1 | Tetra | Hexa |
| 99 | 124 | 0 | Campbell2 | Campbell2 | Tetra | Tetra |
| 100 | 6 | 13 | Campbell5 | Campbell5 | Penta | Penta |
| 100 | 6 | 15 | Campbell5 | Campbell5 | Penta | Penta |
| 100 | 11 | 0 | Campbell5 | Campbell5 | Penta | Penta |
| 100 | 18 | 0 | Campbell5 | Campbell5 | Penta | Penta |
| 100 | 35 | 38 | Campbell5 | Campbell5 | Penta | ? |
| 100 | 35 | 10 | Campbell5 | Campbell5 | Penta | ? |
| 100 | 38 | 10 | Campbell5 | Campbell5 | Penta | Penta |
| 100 | 62 | 30 | Campbell5 | Campbell5 | Penta | Tetra |
| 100 | 64 | 30 | Campbell5 | Campbell5 | Penta | Tetra |
| 100 | 71 | 0 | Campbell5 | Campbell5 | Penta | Penta |
| 100 | 74 | 0 | Campbell5 | Campbell5 | Penta | Penta |
| 100 | 100 | 0 | Campbell5 | Campbell5 | Penta | Penta |
| 100 | 100 | 0 | Campbell5 | Campbell5 | Penta | Penta |
| 100 | 100 | 0 | Campbell5 | Campbell5 | Penta | Penta |
| 100 | 100 | 0 | Campbell5 | Campbell5 | Penta | Penta |
| 100 | 101 | 30 | Campbell5 | Campbell5 | Penta | ? |
| 100 | 106 | 22 | Campbell5 | Campbell5 | Penta | Hexa |
| 101 | 6 | 0 | Campbell5 | Campbell5 | ? | Penta |
| 101 | 11 | 3 | Campbell5 | Campbell5 | ? | Penta |
| 101 | 11 | 0 | Campbell5 | Campbell5 | ? | Penta |
| 101 | 18 | 0 | Campbell5 | Campbell5 | ? | Penta |
| 101 | 35 | 32 | Campbell5 | Campbell5 | ? | ? |
| 101 | 35 | 35 | Campbell5 | Campbell5 | ? | ? |
| 101 | 38 | 0 | Campbell5 | Campbell5 | ? | Penta |
| 101 | 41 | 20 | Campbell5 | Chaplin1 | ? | Hexa |
| 101 | 62 | 39 | Campbell5 | Campbell5 | ? | Tetra |
| 101 | 64 | 0 | Campbell5 | Campbell5 | ? | Tetra |
| 101 | 71 | 0 | Campbell5 | Campbell5 | ? | Penta |
| 101 | 74 | 0 | Campbell5 | Campbell5 | ? | Penta |
| 101 | 100 | 13 | Campbell5 | Campbell5 | ? | Penta |
| 101 | 101 | 1 | Campbell5 | Campbell5 | ? | ? |
| 101 | 101 | 0 | Campbell5 | Campbell5 | ? | ? |
| 101 | 106 | 40 | Campbell5 | Campbell5 | ? | Hexa |
| 102 | 7 | 40 | Little2 | Little2 | Hexa | Hexa |
| 102 | 12 | 1 | Little2 | Little2 | Hexa | Hexa |
| 102 | 17 | 25 | Little2 | Little1 | Hexa | Tetra |
| 102 | 22 | 46 | Little2 | Little2 | Hexa | Hexa |
| 102 | 34 | 35 | Little2 | Little2 | Hexa | Hexa |
| 102 | 45 | 5 | Little2 | Gilmore | Hexa | Tetra |
| 102 | 63 | 25 | Little2 | Little2 | Hexa | Hexa |
| 102 | 65 | 1 | Little2 | Little2 | Hexa | Hexa |
| 102 | 71 | 0 | Little2 | Campbell5 | Hexa | Penta |
| 102 | 78 | 5 | Little2 | Campbell1 | Hexa | Tetra |
| 102 | 87 | 10 | Little2 | Campbell3 | Hexa | Tetra |
| 102 | 90 | 15 | Little2 | Little2 | Hexa | Hexa |
| 102 | 91 | 15 | Little2 | Chaplin1 | Hexa | Hexa |
| 102 | 102 | 2 | Little2 | Little2 | Hexa | Hexa |
| 102 | 102 | 2 | Little2 | Little2 | Hexa | Hexa |
| 102 | 103 | 61 | Little2 | Gilmore | Hexa | Hexa |
| 102 | 111 | 1 | Little2 | Little2 | Hexa | Hexa |
| 102 | 111 | 25 | Little2 | Little2 | Hexa | Hexa |
| 102 | 115 | 25 | Little2 | Little2 | Hexa | Hexa |
| 102 | 118 | 12 | Little2 | Little2 | Hexa | Hexa |
| 103 | 2 | 0 | Gilmore | Gilmore | Hexa | Penta |
| 103 | 29 | 33 | Gilmore | Gilmore | Hexa | Tetra |
| 103 | 30 | 3 | Gilmore | Campbell2 | Hexa | Tetra |
| 103 | 39 | 45 | Gilmore | Gilmore | Hexa | Tetra |
| 103 | 41 | 42 | Gilmore | Chaplin1 | Hexa | Hexa |
| 103 | 45 | 12 | Gilmore | Gilmore | Hexa | Tetra |
| 103 | 46 | 3 | Gilmore | Gilmore | Hexa | Tetra |
| 103 | 51 | 30 | Gilmore | Gilmore | Hexa | Hexa |
| 103 | 51 | 30 | Gilmore | Gilmore | Hexa | Hexa |
| 103 | 52 | 40 | Gilmore | Gilmore | Hexa | Hexa |
| 103 | 53 | 28 | Gilmore | Little1 | Hexa | Hexa |
| 103 | 83 | 50 | Gilmore | Gilmore | Hexa | Hexa |
| 103 | 97 | 19 | Gilmore | Gilmore | Hexa | Tetra |
| 103 | 102 | 25 | Gilmore | Little2 | Hexa | Hexa |
| 103 | 103 | 1 | Gilmore | Gilmore | Hexa | Hexa |
| 103 | 103 | 4 | Gilmore | Gilmore | Hexa | Hexa |
| 103 | 105 | 34 | Gilmore | Gilmore | Hexa | Tetra |
| 103 | 114 | 15 | Gilmore | Gilmore | Hexa | Tetra |
| 103 | 117 | 23 | Gilmore | Chaplin2 | Hexa | Hexa |
| 103 | 118 | 2 | Gilmore | Little2 | Hexa | Hexa |
| 103 | 123 | 5 | Gilmore | Little1 | Hexa | NA |
| 104 | 14 | 40 | Chaplin2 | Campbell4 | Hexa | Tetra |
| 104 | 16 | 20 | Chaplin2 | Chaplin2 | Hexa | Hexa |
| 104 | 19 | 30 | Chaplin2 | Chaplin2 | Hexa | Hexa |
| 104 | 32 | 36 | Chaplin2 | Chaplin2 | Hexa | Hexa |
| 104 | 34 | 30 | Chaplin2 | Little2 | Hexa | Hexa |
| 104 | 41 | 35 | Chaplin2 | Chaplin1 | Hexa | Hexa |
| 104 | 49 | 30 | Chaplin2 | Chaplin2 | Hexa | Hexa |
| 104 | 49 | 9 | Chaplin2 | Chaplin2 | Hexa | Hexa |
| 104 | 66 | 14 | Chaplin2 | Chaplin2 | Hexa | Hexa |
| 104 | 67 | 30 | Chaplin2 | Chaplin1 | Hexa | Hexa |
| 104 | 67 | 25 | Chaplin2 | Chaplin1 | Hexa | Hexa |
| 104 | 67 | 20 | Chaplin2 | Chaplin1 | Hexa | Hexa |
| 104 | 69 | 22 | Chaplin2 | Chaplin2 | Hexa | Hexa |
| 104 | 73 | 25 | Chaplin2 | Campbell4 | Hexa | Tetra |
| 104 | 91 | 30 | Chaplin2 | Chaplin1 | Hexa | Hexa |
| 104 | 104 | 1 | Chaplin2 | Chaplin2 | Hexa | Hexa |
| 104 | 104 | 3 | Chaplin2 | Chaplin2 | Hexa | Hexa |
| 104 | 104 | 0 | Chaplin2 | Chaplin2 | Hexa | Hexa |
| 104 | 104 | 0 | Chaplin2 | Chaplin2 | Hexa | Hexa |
| 104 | 104 | 0 | Chaplin2 | Chaplin2 | Hexa | Hexa |
| 104 | 104 | 0 | Chaplin2 | Chaplin2 | Hexa | Hexa |
| 104 | 104 | 0 | Chaplin2 | Chaplin2 | Hexa | Hexa |
| 104 | 104 | 0 | Chaplin2 | Chaplin2 | Hexa | Hexa |
| 104 | 105 | 23 | Chaplin2 | Gilmore | Hexa | Tetra |
| 104 | 107 | 37 | Chaplin2 | Chaplin1 | Hexa | Hexa |
| 104 | 107 | 35 | Chaplin2 | Chaplin1 | Hexa | Hexa |
| 104 | 110 | 20 | Chaplin2 | Little1 | Hexa | Hexa |
| 104 | 113 | 35 | Chaplin2 | Chaplin2 | Hexa | Hexa |
| 104 | 117 | 3 | Chaplin2 | Chaplin2 | Hexa | Hexa |
| 104 | 117 | 1 | Chaplin2 | Chaplin2 | Hexa | Hexa |
| 104 | 119 | 29 | Chaplin2 | Campbell1 | Hexa | Tetra |
| 105 | 2 | 10 | Gilmore | Gilmore | Tetra | Penta |
| 105 | 6 | 4 | Gilmore | Campbell5 | Tetra | Penta |
| 105 | 18 | 0 | Gilmore | Campbell5 | Tetra | Penta |
| 105 | 21 | 25 | Gilmore | Campbell2 | Tetra | Tetra |
| 105 | 29 | 19 | Gilmore | Gilmore | Tetra | Tetra |
| 105 | 39 | 0 | Gilmore | Gilmore | Tetra | Tetra |
| 105 | 41 | 30 | Gilmore | Chaplin1 | Tetra | Hexa |
| 105 | 45 | 35 | Gilmore | Gilmore | Tetra | Tetra |
| 105 | 46 | 2 | Gilmore | Gilmore | Tetra | Tetra |
| 105 | 50 | 10 | Gilmore | Campbell4 | Tetra | Tetra |
| 105 | 51 | 0 | Gilmore | Gilmore | Tetra | Hexa |
| 105 | 52 | 40 | Gilmore | Gilmore | Tetra | Hexa |
| 105 | 73 | 20 | Gilmore | Campbell4 | Tetra | Tetra |
| 105 | 78 | 12 | Gilmore | Campbell1 | Tetra | Tetra |
| 105 | 83 | 0 | Gilmore | Gilmore | Tetra | Hexa |
| 105 | 88 | 0 | Gilmore | Campbell3 | Tetra | Tetra |
| 105 | 97 | 19 | Gilmore | Gilmore | Tetra | Tetra |
| 105 | 103 | 29 | Gilmore | Gilmore | Tetra | Hexa |
| 105 | 104 | 33 | Gilmore | Chaplin2 | Tetra | Hexa |
| 105 | 105 | 40 | Gilmore | Gilmore | Tetra | Tetra |
| 105 | 105 | 30 | Gilmore | Gilmore | Tetra | Tetra |
| 105 | 114 | 40 | Gilmore | Gilmore | Tetra | Tetra |
| 106 | 11 | 7 | Campbell5 | Campbell5 | Hexa | Penta |
| 106 | 18 | 0 | Campbell5 | Campbell5 | Hexa | Penta |
| 106 | 35 | 25 | Campbell5 | Campbell5 | Hexa | ? |
| 106 | 38 | 20 | Campbell5 | Campbell5 | Hexa | Penta |
| 106 | 62 | 10 | Campbell5 | Campbell5 | Hexa | Tetra |
| 106 | 64 | 10 | Campbell5 | Campbell5 | Hexa | Tetra |
| 106 | 71 | 0 | Campbell5 | Campbell5 | Hexa | Penta |
| 106 | 74 | 5 | Campbell5 | Campbell5 | Hexa | Penta |
| 106 | 100 | 25 | Campbell5 | Campbell5 | Hexa | Penta |
| 106 | 101 | 11 | Campbell5 | Campbell5 | Hexa | ? |
| 106 | 106 | 0 | Campbell5 | Campbell5 | Hexa | Hexa |
| 107 | 1 | 30 | Chaplin1 | Chaplin1 | Hexa | Hexa |
| 107 | 5 | 20 | Chaplin1 | Chaplin1 | Hexa | Hexa |
| 107 | 7 | 40 | Chaplin1 | Little2 | Hexa | Hexa |
| 107 | 41 | 22 | Chaplin1 | Chaplin1 | Hexa | Hexa |
| 107 | 42 | 0 | Chaplin1 | Campbell4 | Hexa | Penta |
| 107 | 54 | 33 | Chaplin1 | Chaplin1 | Hexa | Hexa |
| 107 | 67 | 6 | Chaplin1 | Chaplin1 | Hexa | Hexa |
| 107 | 78 | 24 | Chaplin1 | Campbell1 | Hexa | Tetra |
| 107 | 89 | 26 | Chaplin1 | Chaplin1 | Hexa | Hexa |
| 107 | 91 | 40 | Chaplin1 | Chaplin1 | Hexa | Hexa |
| 107 | 91 | 30 | Chaplin1 | Chaplin1 | Hexa | Hexa |
| 107 | 96 | 42 | Chaplin1 | Campbell2 | Hexa | Tetra |
| 107 | 104 | 39 | Chaplin1 | Chaplin2 | Hexa | Hexa |
| 107 | 104 | 35 | Chaplin1 | Chaplin2 | Hexa | Hexa |
| 107 | 107 | 0 | Chaplin1 | Chaplin1 | Hexa | Hexa |
| 107 | 107 | 5 | Chaplin1 | Chaplin1 | Hexa | Hexa |
| 107 | 122 | 25 | Chaplin1 | Chaplin1 | Hexa | Hexa |
| 108 | 14 | 30 | Campbell3 | Campbell4 | Tetra | Tetra |
| 108 | 17 | 15 | Campbell3 | Little1 | Tetra | Tetra |
| 108 | 27 | 15 | Campbell3 | Campbell3 | Tetra | Tetra |
| 108 | 48 | 0 | Campbell3 | Campbell3 | Tetra | Tetra |
| 108 | 56 | 25 | Campbell3 | Campbell3 | Tetra | Tetra |
| 108 | 58 | 1 | Campbell3 | Campbell3 | Tetra | Tetra |
| 108 | 81 | 0 | Campbell3 | Campbell3 | Tetra | Tetra |
| 108 | 87 | 70 | Campbell3 | Campbell3 | Tetra | Tetra |
| 108 | 88 | 7 | Campbell3 | Campbell3 | Tetra | Tetra |
| 108 | 92 | 20 | Campbell3 | Campbell3 | Tetra | Tetra |
| 108 | 98 | 0 | Campbell3 | Campbell3 | Tetra | Tetra |
| 108 | 108 | 0 | Campbell3 | Campbell3 | Tetra | Tetra |
| 108 | 108 | 7 | Campbell3 | Campbell3 | Tetra | Tetra |
| 108 | 109 | 6 | Campbell3 | Campbell3 | Tetra | Tetra |
| 108 | 121 | 20 | Campbell3 | Campbell3 | Tetra | Tetra |
| 109 | 2 | 16 | Campbell3 | Gilmore | Tetra | Penta |
| 109 | 2 | 0 | Campbell3 | Gilmore | Tetra | Penta |
| 109 | 27 | 0 | Campbell3 | Campbell3 | Tetra | Tetra |
| 109 | 48 | 10 | Campbell3 | Campbell3 | Tetra | Tetra |
| 109 | 56 | 7 | Campbell3 | Campbell3 | Tetra | Tetra |
| 109 | 58 | 20 | Campbell3 | Campbell3 | Tetra | Tetra |
| 109 | 87 | 0 | Campbell3 | Campbell3 | Tetra | Tetra |
| 109 | 88 | 40 | Campbell3 | Campbell3 | Tetra | Tetra |
| 109 | 92 | 20 | Campbell3 | Campbell3 | Tetra | Tetra |
| 109 | 95 | 2 | Campbell3 | Campbell4 | Tetra | Hexa |
| 109 | 98 | 20 | Campbell3 | Campbell3 | Tetra | Tetra |
| 109 | 98 | 25 | Campbell3 | Campbell3 | Tetra | Tetra |
| 109 | 108 | 20 | Campbell3 | Campbell3 | Tetra | Tetra |
| 109 | 109 | 3 | Campbell3 | Campbell3 | Tetra | Tetra |
| 109 | 109 | 0 | Campbell3 | Campbell3 | Tetra | Tetra |
| 109 | 121 | 20 | Campbell3 | Campbell3 | Tetra | Tetra |
| 110 | 7 | 25 | Little1 | Little2 | Hexa | Hexa |
| 110 | 17 | 45 | Little1 | Little1 | Hexa | Tetra |
| 110 | 18 | 0 | Little1 | Campbell5 | Hexa | Penta |
| 110 | 33 | 1 | Little1 | Little1 | Hexa | Hexa |
| 110 | 37 | 14 | Little1 | Little1 | Hexa | Hexa |
| 110 | 41 | 36 | Little1 | Chaplin1 | Hexa | Hexa |
| 110 | 46 | 15 | Little1 | Gilmore | Hexa | Tetra |
| 110 | 47 | 20 | Little1 | Little1 | Hexa | Tetra |
| 110 | 53 | 25 | Little1 | Little1 | Hexa | Hexa |
| 110 | 65 | 30 | Little1 | Little2 | Hexa | Hexa |
| 110 | 72 | 10 | Little1 | Little1 | Hexa | Tetra |
| 110 | 74 | 2 | Little1 | Campbell5 | Hexa | Penta |
| 110 | 99 | 25 | Little1 | Campbell2 | Hexa | Tetra |
| 110 | 99 | 25 | Little1 | Campbell2 | Hexa | Tetra |
| 110 | 104 | 20 | Little1 | Chaplin2 | Hexa | Hexa |
| 110 | 110 | 1 | Little1 | Little1 | Hexa | Hexa |
| 110 | 110 | 0 | Little1 | Little1 | Hexa | Hexa |
| 110 | 116 | 50 | Little1 | Little1 | Hexa | Hexa |
| 110 | 119 | 40 | Little1 | Campbell1 | Hexa | Tetra |
| 110 | 119 | 25 | Little1 | Campbell1 | Hexa | Tetra |
| 110 | 120 | 7 | Little1 | Little1 | Hexa | Tetra |
| 110 | 123 | 50 | Little1 | Little1 | Hexa | NA |
| 111 | 7 | 30 | Little2 | Little2 | Hexa | Hexa |
| 111 | 12 | 30 | Little2 | Little2 | Hexa | Hexa |
| 111 | 22 | 21 | Little2 | Little2 | Hexa | Hexa |
| 111 | 34 | 24 | Little2 | Little2 | Hexa | Hexa |
| 111 | 63 | 30 | Little2 | Little2 | Hexa | Hexa |
| 111 | 65 | 0 | Little2 | Little2 | Hexa | Hexa |
| 111 | 90 | 0 | Little2 | Little2 | Hexa | Hexa |
| 111 | 102 | 2 | Little2 | Little2 | Hexa | Hexa |
| 111 | 102 | 30 | Little2 | Little2 | Hexa | Hexa |
| 111 | 111 | 2 | Little2 | Little2 | Hexa | Hexa |
| 111 | 111 | 2 | Little2 | Little2 | Hexa | Hexa |
| 111 | 115 | 20 | Little2 | Little2 | Hexa | Hexa |
| 111 | 118 | 0 | Little2 | Little2 | Hexa | Hexa |
| 113 | 7 | 0 | Chaplin2 | Little2 | Hexa | Hexa |
| 113 | 16 | 5 | Chaplin2 | Chaplin2 | Hexa | Hexa |
| 113 | 19 | 0 | Chaplin2 | Chaplin2 | Hexa | Hexa |
| 113 | 32 | 15 | Chaplin2 | Chaplin2 | Hexa | Hexa |
| 113 | 32 | 16 | Chaplin2 | Chaplin2 | Hexa | Hexa |
| 113 | 49 | 40 | Chaplin2 | Chaplin2 | Hexa | Hexa |
| 113 | 66 | 15 | Chaplin2 | Chaplin2 | Hexa | Hexa |
| 113 | 69 | 12 | Chaplin2 | Chaplin2 | Hexa | Hexa |
| 113 | 104 | 40 | Chaplin2 | Chaplin2 | Hexa | Hexa |
| 113 | 113 | 3 | Chaplin2 | Chaplin2 | Hexa | Hexa |
| 113 | 113 | 0 | Chaplin2 | Chaplin2 | Hexa | Hexa |
| 113 | 114 | 15 | Chaplin2 | Gilmore | Hexa | Tetra |
| 113 | 117 | 10 | Chaplin2 | Chaplin2 | Hexa | Hexa |
| 114 | 2 | 12 | Gilmore | Gilmore | Tetra | Penta |
| 114 | 29 | 0 | Gilmore | Gilmore | Tetra | Tetra |
| 114 | 39 | 1 | Gilmore | Gilmore | Tetra | Tetra |
| 114 | 45 | 6 | Gilmore | Gilmore | Tetra | Tetra |
| 114 | 46 | 30 | Gilmore | Gilmore | Tetra | Tetra |
| 114 | 51 | 30 | Gilmore | Gilmore | Tetra | Hexa |
| 114 | 51 | 30 | Gilmore | Gilmore | Tetra | Hexa |
| 114 | 52 | 6 | Gilmore | Gilmore | Tetra | Hexa |
| 114 | 83 | 25 | Gilmore | Gilmore | Tetra | Hexa |
| 114 | 97 | 0 | Gilmore | Gilmore | Tetra | Tetra |
| 114 | 103 | 30 | Gilmore | Gilmore | Tetra | Hexa |
| 114 | 105 | 20 | Gilmore | Gilmore | Tetra | Tetra |
| 114 | 114 | 1 | Gilmore | Gilmore | Tetra | Tetra |
| 114 | 114 | 1 | Gilmore | Gilmore | Tetra | Tetra |
| 114 | 114 | 0 | Gilmore | Gilmore | Tetra | Tetra |
| 114 | 114 | 0 | Gilmore | Gilmore | Tetra | Tetra |
| 114 | 122 | 8 | Gilmore | Chaplin1 | Tetra | Hexa |
| 115 | 7 | 0 | Little2 | Little2 | Hexa | Hexa |
| 115 | 12 | 0 | Little2 | Little2 | Hexa | Hexa |
| 115 | 22 | 2 | Little2 | Little2 | Hexa | Hexa |
| 115 | 22 | 0 | Little2 | Little2 | Hexa | Hexa |
| 115 | 34 | 35 | Little2 | Little2 | Hexa | Hexa |
| 115 | 63 | 2 | Little2 | Little2 | Hexa | Hexa |
| 115 | 65 | 0 | Little2 | Little2 | Hexa | Hexa |
| 115 | 90 | 30 | Little2 | Little2 | Hexa | Hexa |
| 115 | 102 | 35 | Little2 | Little2 | Hexa | Hexa |
| 115 | 111 | 5 | Little2 | Little2 | Hexa | Hexa |
| 115 | 115 | 0 | Little2 | Little2 | Hexa | Hexa |
| 115 | 115 | 0 | Little2 | Little2 | Hexa | Hexa |
| 115 | 118 | 7 | Little2 | Little2 | Hexa | Hexa |
| 115 | 118 | 0 | Little2 | Little2 | Hexa | Hexa |
| 116 | 11 | 3 | Little1 | Campbell5 | Hexa | Penta |
| 116 | 17 | 30 | Little1 | Little1 | Hexa | Tetra |
| 116 | 22 | 40 | Little1 | Little2 | Hexa | Hexa |
| 116 | 33 | 40 | Little1 | Little1 | Hexa | Hexa |
| 116 | 37 | 30 | Little1 | Little1 | Hexa | Hexa |
| 116 | 41 | 37 | Little1 | Chaplin1 | Hexa | Hexa |
| 116 | 47 | 30 | Little1 | Little1 | Hexa | Tetra |
| 116 | 51 | 36 | Little1 | Gilmore | Hexa | Hexa |
| 116 | 53 | 40 | Little1 | Little1 | Hexa | Hexa |
| 116 | 67 | 45 | Little1 | Chaplin1 | Hexa | Hexa |
| 116 | 72 | 30 | Little1 | Little1 | Hexa | Tetra |
| 116 | 72 | 20 | Little1 | Little1 | Hexa | Tetra |
| 116 | 95 | 23 | Little1 | Campbell4 | Hexa | Hexa |
| 116 | 110 | 30 | Little1 | Little1 | Hexa | Hexa |
| 116 | 116 | 1 | Little1 | Little1 | Hexa | Hexa |
| 116 | 116 | 0 | Little1 | Little1 | Hexa | Hexa |
| 116 | 116 | 0 | Little1 | Little1 | Hexa | Hexa |
| 116 | 116 | 0 | Little1 | Little1 | Hexa | Hexa |
| 116 | 120 | 15 | Little1 | Little1 | Hexa | Tetra |
| 116 | 123 | 0 | Little1 | Little1 | Hexa | NA |
| 117 | 16 | 17 | Chaplin2 | Chaplin2 | Hexa | Hexa |
| 117 | 19 | 12 | Chaplin2 | Chaplin2 | Hexa | Hexa |
| 117 | 32 | 17 | Chaplin2 | Chaplin2 | Hexa | Hexa |
| 117 | 49 | 12 | Chaplin2 | Chaplin2 | Hexa | Hexa |
| 117 | 63 | 25 | Chaplin2 | Little2 | Hexa | Hexa |
| 117 | 66 | 4 | Chaplin2 | Chaplin2 | Hexa | Hexa |
| 117 | 69 | 30 | Chaplin2 | Chaplin2 | Hexa | Hexa |
| 117 | 104 | 0 | Chaplin2 | Chaplin2 | Hexa | Hexa |
| 117 | 104 | 0 | Chaplin2 | Chaplin2 | Hexa | Hexa |
| 117 | 113 | 25 | Chaplin2 | Chaplin2 | Hexa | Hexa |
| 117 | 117 | 0 | Chaplin2 | Chaplin2 | Hexa | Hexa |
| 117 | 117 | 3 | Chaplin2 | Chaplin2 | Hexa | Hexa |
| 117 | 119 | 25 | Chaplin2 | Campbell1 | Hexa | Tetra |
| 117 | 122 | 0 | Chaplin2 | Chaplin1 | Hexa | Hexa |
| 118 | 7 | 0 | Little2 | Little2 | Hexa | Hexa |
| 118 | 11 | 3 | Little2 | Campbell5 | Hexa | Penta |
| 118 | 12 | 3 | Little2 | Little2 | Hexa | Hexa |
| 118 | 22 | 18 | Little2 | Little2 | Hexa | Hexa |
| 118 | 34 | 40 | Little2 | Little2 | Hexa | Hexa |
| 118 | 63 | 55 | Little2 | Little2 | Hexa | Hexa |
| 118 | 65 | 17 | Little2 | Little2 | Hexa | Hexa |
| 118 | 66 | 15 | Little2 | Chaplin2 | Hexa | Hexa |
| 118 | 73 | 12 | Little2 | Campbell4 | Hexa | Tetra |
| 118 | 82 | 20 | Little2 | Campbell2 | Hexa | NA |
| 118 | 90 | 11 | Little2 | Little2 | Hexa | Hexa |
| 118 | 98 | 25 | Little2 | Campbell3 | Hexa | Tetra |
| 118 | 102 | 35 | Little2 | Little2 | Hexa | Hexa |
| 118 | 103 | 2 | Little2 | Gilmore | Hexa | Hexa |
| 118 | 111 | 30 | Little2 | Little2 | Hexa | Hexa |
| 118 | 115 | 8 | Little2 | Little2 | Hexa | Hexa |
| 118 | 115 | 8 | Little2 | Little2 | Hexa | Hexa |
| 118 | 118 | 0 | Little2 | Little2 | Hexa | Hexa |
| 118 | 118 | 0 | Little2 | Little2 | Hexa | Hexa |
| 118 | 120 | 40 | Little2 | Little1 | Hexa | Tetra |
| 118 | 120 | 15 | Little2 | Little1 | Hexa | Tetra |
| 119 | 10 | 25 | Campbell1 | Campbell1 | Tetra | Tetra |
| 119 | 17 | 15 | Campbell1 | Little1 | Tetra | Tetra |
| 119 | 22 | 1 | Campbell1 | Little2 | Tetra | Hexa |
| 119 | 22 | 20 | Campbell1 | Little2 | Tetra | Hexa |
| 119 | 25 | 25 | Campbell1 | Campbell1 | Tetra | Tetra |
| 119 | 50 | 3 | Campbell1 | Campbell4 | Tetra | Tetra |
| 119 | 57 | 30 | Campbell1 | Campbell1 | Tetra | Tetra |
| 119 | 77 | 0 | Campbell1 | Campbell1 | Tetra | Tetra |
| 119 | 78 | 35 | Campbell1 | Campbell1 | Tetra | Tetra |
| 119 | 78 | 35 | Campbell1 | Campbell1 | Tetra | Tetra |
| 119 | 86 | 30 | Campbell1 | Campbell1 | Tetra | Tetra |
| 119 | 104 | 31 | Campbell1 | Chaplin2 | Tetra | Hexa |
| 119 | 119 | 0 | Campbell1 | Campbell1 | Tetra | Tetra |
| 119 | 119 | 0 | Campbell1 | Campbell1 | Tetra | Tetra |
| 120 | 7 | 51 | Little1 | Little2 | Tetra | Hexa |
| 120 | 17 | 7 | Little1 | Little1 | Tetra | Tetra |
| 120 | 33 | 3 | Little1 | Little1 | Tetra | Hexa |
| 120 | 37 | 60 | Little1 | Little1 | Tetra | Hexa |
| 120 | 38 | 20 | Little1 | Campbell5 | Tetra | Penta |
| 120 | 45 | 0 | Little1 | Gilmore | Tetra | Tetra |
| 120 | 46 | 0 | Little1 | Gilmore | Tetra | Tetra |
| 120 | 47 | 0 | Little1 | Little1 | Tetra | Tetra |
| 120 | 53 | 0 | Little1 | Little1 | Tetra | Hexa |
| 120 | 63 | 50 | Little1 | Little2 | Tetra | Hexa |
| 120 | 72 | 46 | Little1 | Little1 | Tetra | Tetra |
| 120 | 81 | 30 | Little1 | Campbell3 | Tetra | Tetra |
| 120 | 91 | 46 | Little1 | Chaplin1 | Tetra | Hexa |
| 120 | 110 | 30 | Little1 | Little1 | Tetra | Hexa |
| 120 | 116 | 30 | Little1 | Little1 | Tetra | Hexa |
| 120 | 118 | 40 | Little1 | Little2 | Tetra | Hexa |
| 120 | 118 | 7 | Little1 | Little2 | Tetra | Hexa |
| 120 | 120 | 0 | Little1 | Little1 | Tetra | Tetra |
| 120 | 120 | 0 | Little1 | Little1 | Tetra | Tetra |
| 120 | 123 | 40 | Little1 | Little1 | Tetra | NA |
| 120 | 124 | 20 | Little1 | Campbell2 | Tetra | Tetra |
| 121 | 27 | 10 | Campbell3 | Campbell3 | Tetra | Tetra |
| 121 | 40 | 20 | Campbell3 | Campbell4 | Tetra | Penta |
| 121 | 48 | 0 | Campbell3 | Campbell3 | Tetra | Tetra |
| 121 | 56 | 30 | Campbell3 | Campbell3 | Tetra | Tetra |
| 121 | 58 | 40 | Campbell3 | Campbell3 | Tetra | Tetra |
| 121 | 73 | 10 | Campbell3 | Campbell4 | Tetra | Tetra |
| 121 | 81 | 0 | Campbell3 | Campbell3 | Tetra | Tetra |
| 121 | 87 | 30 | Campbell3 | Campbell3 | Tetra | Tetra |
| 121 | 88 | 20 | Campbell3 | Campbell3 | Tetra | Tetra |
| 121 | 92 | 0 | Campbell3 | Campbell3 | Tetra | Tetra |
| 121 | 98 | 30 | Campbell3 | Campbell3 | Tetra | Tetra |
| 121 | 108 | 15 | Campbell3 | Campbell3 | Tetra | Tetra |
| 121 | 109 | 35 | Campbell3 | Campbell3 | Tetra | Tetra |
| 121 | 121 | 0 | Campbell3 | Campbell3 | Tetra | Tetra |
| 121 | 121 | 2 | Campbell3 | Campbell3 | Tetra | Tetra |
| 122 | 1 | 12 | Chaplin1 | Chaplin1 | Hexa | Hexa |
| 122 | 5 | 0 | Chaplin1 | Chaplin1 | Hexa | Hexa |
| 122 | 12 | 25 | Chaplin1 | Little2 | Hexa | Hexa |
| 122 | 14 | 30 | Chaplin1 | Campbell4 | Hexa | Tetra |
| 122 | 27 | 10 | Chaplin1 | Campbell3 | Hexa | Tetra |
| 122 | 41 | 20 | Chaplin1 | Chaplin1 | Hexa | Hexa |
| 122 | 54 | 30 | Chaplin1 | Chaplin1 | Hexa | Hexa |
| 122 | 67 | 20 | Chaplin1 | Chaplin1 | Hexa | Hexa |
| 122 | 77 | 1 | Chaplin1 | Campbell1 | Hexa | Tetra |
| 122 | 89 | 20 | Chaplin1 | Chaplin1 | Hexa | Hexa |
| 122 | 91 | 20 | Chaplin1 | Chaplin1 | Hexa | Hexa |
| 122 | 107 | 8 | Chaplin1 | Chaplin1 | Hexa | Hexa |
| 122 | 117 | 2 | Chaplin1 | Chaplin2 | Hexa | Hexa |
| 122 | 122 | 0 | Chaplin1 | Chaplin1 | Hexa | Hexa |
| 122 | 122 | 0 | Chaplin1 | Chaplin1 | Hexa | Hexa |
| 123 | 7 | 15 | Little1 | Little2 | NA | Hexa |
| 123 | 33 | 22 | Little1 | Little1 | NA | Hexa |
| 123 | 37 | 0 | Little1 | Little1 | NA | Hexa |
| 123 | 53 | 25 | Little1 | Little1 | NA | Hexa |
| 123 | 53 | 10 | Little1 | Little1 | NA | Hexa |
| 123 | 63 | 5 | Little1 | Little2 | NA | Hexa |
| 123 | 103 | 0 | Little1 | Gilmore | NA | Hexa |
| 123 | 110 | 55 | Little1 | Little1 | NA | Hexa |
| 123 | 116 | 1 | Little1 | Little1 | NA | Hexa |
| 123 | 120 | 30 | Little1 | Little1 | NA | Tetra |
| 124 | 2 | 4 | Campbell2 | Gilmore | Tetra | Penta |
| 124 | 4 | 15 | Campbell2 | Campbell2 | Tetra | Tetra |
| 124 | 9 | 2 | Campbell2 | Campbell2 | Tetra | Tetra |
| 124 | 21 | 20 | Campbell2 | Campbell2 | Tetra | Tetra |
| 124 | 30 | 3 | Campbell2 | Campbell2 | Tetra | Tetra |
| 124 | 36 | 7 | Campbell2 | Campbell2 | Tetra | Tetra |
| 124 | 73 | 15 | Campbell2 | Campbell4 | Tetra | Tetra |
| 124 | 82 | 12 | Campbell2 | Campbell2 | Tetra | NA |
| 124 | 93 | 0 | Campbell2 | Campbell2 | Tetra | Tetra |
| 124 | 96 | 9 | Campbell2 | Campbell2 | Tetra | Tetra |
| 124 | 120 | 12 | Campbell2 | Little1 | Tetra | Tetra |
| 124 | 120 | 12 | Campbell2 | Little1 | Tetra | Tetra |
| 124 | 124 | 0 | Campbell2 | Campbell2 | Tetra | Tetra |
| 401 | 28 | 30 | NA | Campbell4 | NA | Tetra |
