# Supplementary material for: Genetic rescue in a plant polyploid complex: Case study on the importance of genetic and trait data for conservation management
Source: Ecol Evol. 2018 Apr 25;8(10):5153–63. doi: 10.1002/ece3.4039 (PMC5980434; doi:10.1002/ece3.4039)
Supplement: Supplementary file 3 [file ECE3-8-5153-s003.docx]

**Supplementary data 3.** Crossing diallels for ten populations of the rare Australian daisy *Rutidosis lanata*. Plus indicates crossing success (7 or more fertile seeds), minus indicates crossing failure, empty cells indicate missing data or ambiguous results. Mothers are in rows, fathers in columns.

Campbell 1, tetraploid

10 25 57 77 78 86 119

**-** **-** **+** **-** **+** **-** **+**

**-** **-** **+** **-** **+** **-** **+**

**+** **+** **-** **+** **+** **+** **+**

**+** **-** **-** **-**

**+** **+** **+** **+** **-** **+** **+**

**+** **+** **+** **-** **+**

**+** **+** **+** **-** **+** **+** **-**

Campbell 2, tetraploid

4 9 21 26 30 36 93 96 99 124

**-** **+** **+** **+** **+** **-** **-** **+**

**+** **-** **+** **+** **+** **-** **+** **+** **+** **+**

**+** **+** **-** **-** **+** **-** **+** **+** **-**

**-** **-** **+** **-** **-** **-** **-** **+**

**+** **+** **-** **-** **-** **-** **+**

**-** **-** **-** **+** **-** **-** **-** **+** **+**

**+** **+** **-** **-** **-** **-** **-** **-**

**+** **+** **+** **-** **+** **-** **-** **+** **+**

**+** **-** **+** **+** **-** **-** **-** **-** **-**

**+** **-** **+** **-** **+** **-** **+** **-**

Campbell 3, tetraploid

27 48 56 58 81 87 88 92 98 108 109 121

**-** **-** **+** **-** **+** **+** **-** **-** **+** **+** **-**

**-** **-** **-** **-** **+** **-** **+** **-** **-** **-** **-**

**-** **-** **-** **-** **-** **-** **+** **-** **-** **-** **+**

**+** **-** **-** **-** **-** **+** **+** **-** **-** **+** **+**

**-** **-** **-** **-** **-** **+** **-** **+** **+** **-** **-** **-**

**+** **+** **+** **+** **+** **-** **-** **+** **+** **+** **-** **+**

**+** **+** **-** **+** **+** **+** **-** **+** **+** **+** **+** **-**

**-** **+** **+** **+** **+** **+** **+** **-** **+** **+** **+** **+**

**-** **-** **-** **-** **+** **+** **-** **-** **-** **+** **+**

**+** **-** **+** **-** **-** **+** **+** **+** **-** **-** **+**

**-** **+** **+** **+** **-** **+** **+** **+** **+** **-** **+**

**+** **-** **+** **+** **-** **+** **+** **-** **+** **+** **+** **-**

Campbell 4, tetraploid (minority cytotypes excluded)

13 14 28 31 50 61 73 79 94

**-** **-** **-** **-** **-** **+** **+** **+**

**-** **-** **-** **-** **+** **-** **+** **-** **+**

**-** **-** **-** **-** **+** **+** **+** **-** **+**

**-** **+** **-** **-** **+** **-** **+** **-** **+**

**-** **+** **+** **+** **-** **-** **+**

**-** **-** **-** **-** **+** **-** **+** **-** **+**

**+** **+** **-** **+** **-** **-** **-** **+** **+**

**-** **-** **-** **-** **+** **-** **+** **-** **+**

**-** **+** **-** **+** **+** **-** **+** **+** **-**

Chaplin 1, hexaploid

1 5 41 54 67 89 91 107 122

**-** **+** **-** **-** **+** **-** **+** **+** **-**

**+** **+** **+** **+** **-** **+** **+** **+**

**-** **+** **-** **+** **+** **+** **+** **+** **+**

**-** **+** **-** **-** **+** **-** **+** **+** **-**

**+** **+** **+** **+** **-** **+** **+** **-** **+**

**-** **-** **+** **+** **-** **-** **+** **-** **+**

**+** **+** **+** **+** **+** **+** **-** **+** **+**

**+** **+** **+** **+** **-** **+** **+** **-** **+**

**+** **-** **+** **+** **+** **+** **+** **+** **-**

Chaplin 2, hexaploid

16 19 32 49 66 69 104 113 117

**-** **-** **-** **-** **+** **-** **-** **+**

**+** **-** **+** **+** **+** **+** **+** **-** **+**

**-** **-** **-** **+** **+** **-** **+**

**+** **+** **-** **-** **+** **+** **+**

**+** **+** **+** **-** **+** **+** **-**

**-** **-** **-** **+** **+**

**+** **+** **+** **+** **+** **+** **-** **+** **-**

**-** **-** **+** **+** **+** **+** **+** **-** **+**

**+** **+** **+** **+** **-** **+** **-** **+** **-**

Gilmore, tetraploid (minority cytotypes excluded)

29 39 45 46 97 105 114

**-** **+** **-** **+** **+** **+** **+**

**+** **-** **+** **-** **-** **-** **-**

**+** **-** **-** **+** **-** **+** **-**

**+** **-** **+** **-** **+** **-** **+**

**+** **-** **+** **-** **+** **-**

**+** **-** **+** **-** **+** **+** **+**

**-** **-** **-** **+** **-** **+** **-**

Little 1, hexaploid (minority cytotypes excluded)

33 37 53 110 116

**-** **-** **-** **-** **+**

**+** **-** **+** **+** **+**

**-** **-** **-** **+** **+**

**-** **+** **+** **-** **+**

**+** **+** **+** **+** **-**

Little 2, hexaploid

7 12 22 34 63 65 90 102 111 115 118

**-** **+** **-** **+** **+** **+** **+** **+** **-** **-**

**+** **-** **+** **+** **+** **+** **-** **+** **-** **+**

**-** **+** **-** **+** **-** **+** **-** **+** **+** **-** **-**

**+** **+** **+** **-** **+** **-** **-** **+** **-** **+** **+**

**+** **-** **+** **-** **+** **+** **+** **-** **-** **+**

**+** **+** **-** **+** **-** **-** **-** **+** **-**

**+** **+** **+** **+** **-** **-** **+** **-** **+** **-**

**+** **-** **+** **+** **+** **-** **+** **-** **+** **+**

**+** **+** **+** **+** **+** **-** **-** **-** **+** **-**

**-** **-** **-** **+** **-** **-** **+** **+** **-** **-**

**-** **-** **+** **+** **+** **+** **+** **+** **+** **+** **-**
